# PandaGUT provides new insights into bacterial diversity, function, and resistome landscapes with implications for conservation

**DOI:** 10.1186/s40168-023-01657-0

**Published:** 2023-10-07

**Authors:** Guangping Huang, Wenyu Shi, Le Wang, Qingyue Qu, Zhenqiang Zuo, Jinfeng Wang, Fangqing Zhao, Fuwen Wei

**Affiliations:** 1 CAS Key Laboratory of Animal Ecology and Conservation Biology, Institute of Zoology, Chinese Academy of Sciences, Beijing, 100101 China; 2 Microbial Resource and Big Data Center, Institute of Microbiology, Chinese Academy of Sciences, Beijing, 100101 China; 3 Laboratory for Computational Genomics, Beijing Institutes of Life Science, Chinese Academy of Sciences, Beijing, 100101 China; 4 College of Forestry, Jiangxi Agricultural University, Nanchang, 330045 China; 5 University of Chinese Academy of Sciences, Beijing, 100049 China

**Keywords:** Wild mammal, Gut microbiome, Giant panda, Diversity, Adaptive evolution, Conservation

## Abstract

**Background:**

The gut microbiota play important roles in host adaptation and evolution, but are understudied in natural population of wild mammals. To address host adaptive evolution and improve conservation efforts of threatened mammals from a metagenomic perspective, we established a high-quality gut microbiome catalog of the giant panda (pandaGUT) to resolve the microbiome diversity, functional, and resistome landscapes using approximately 7 Tbp of long- and short-read sequencing data from 439 stool samples.

**Results:**

The pandaGUT catalog comprises 820 metagenome-assembled genomes, including 40 complete closed genomes, and 64.5% of which belong to species that have not been previously reported, greatly expanding the coverage of most prokaryotic lineages. The catalog contains 2.37 million unique genes, with 74.8% possessing complete open read frames, facilitating future mining of microbial functional potential. We identified three microbial enterotypes across wild and captive panda populations characterized by *Clostridium*, *Pseudomonas*, and *Escherichia*, respectively. We found that wild pandas exhibited host genetic-specific microbial structures and functions, suggesting host-gut microbiota phylosymbiosis, while the captive cohorts encoded more multi-drug resistance genes.

**Conclusions:**

Our study provides largely untapped resources for biochemical and biotechnological applications as well as potential intervention avenues via the rational manipulation of microbial diversity and reducing antibiotic usage for future conservation management of wildlife.

Video Abstract

**Supplementary Information:**

The online version contains supplementary material available at 10.1186/s40168-023-01657-0.

## Introduction

Gut microbiomes play critical roles in host ecological adaptation, disease resistance, and physiological fitness, with important conservation implications for threatened species [1–7]. Furthermore, wildlife gut microbiota are increasingly recognized as harboring vast biochemical and metabolic potential that can contribute to improving host fitness [8–12]. A comprehensive understanding of the diversity and functional repertoire of gut microbiomes is important for understanding their role in host ecological adaptation, thereby facilitating the guidance of conservation decisions and management policies. However, wildlife gut microbiomes, especially those of threatened species, are very incompletely understood. The lack of high-quality reference genomes has become an obstacle for science-informed biodiversity conservation.

The giant panda (*Ailuropoda melanoleuca*) is a flagship species for global biodiversity conservation and is categorized as “vulnerable” within the International Union for Conservation of Nature (IUCN) Red List [13]. Protecting giant pandas also protects other sympatric species in addition to the entire ecosystem. Giant pandas belong to the order Carnivora and have evolved as exclusive bamboo specialists during their nearly eight million years of evolutionary history [14]. Only 1864 wild giant pandas are extant and are distributed in six mountain ranges including the Qinling (QIN), Minshan (MIN), Qionglai (QIO), Daxiangling (DXL), Xiaoxiangling (XXL), and Liangshan (LSH) mountains that can be classified into three genetic populations including the QIN, MIN, and QIO-DXL-XXL-LSH (QXL) populations [15]. Considerable efforts have been made to understand the roles of gut microbiomes in dietary adaptations via 16S ribosomal RNA (rRNA) gene sequencing and shotgun metagenomic sequencing [16–20], revealing the great potential to degrade fiber, detoxify cyanide, and degrade bamboo flavonoids that are known for their health benefits. Nevertheless, lacking a unified and comprehensive gut microbiome catalog for the giant panda hampers the exploration for host-microbiota coevolution and conservation.

Here, we present a comprehensive panda gut microbiome catalog (termed pandaGUT), established with Nanopore, Pacbio, and Illumina sequencing data across highly diverse samples to address host-microbe coevolution and improve conservation efforts. These highly comprehensive gut microbiome resources for the threatened species will not only substantially expand prokaryotic genomic representation, but also has far-reaching implications for future research into the adaptive evolution and conservation of wildlife.

## Results

### The recovery of over 800 metagenome-assembled genomes with 40 being complete closed bacterial genomes in pandaGUT

**Fig. 1:**
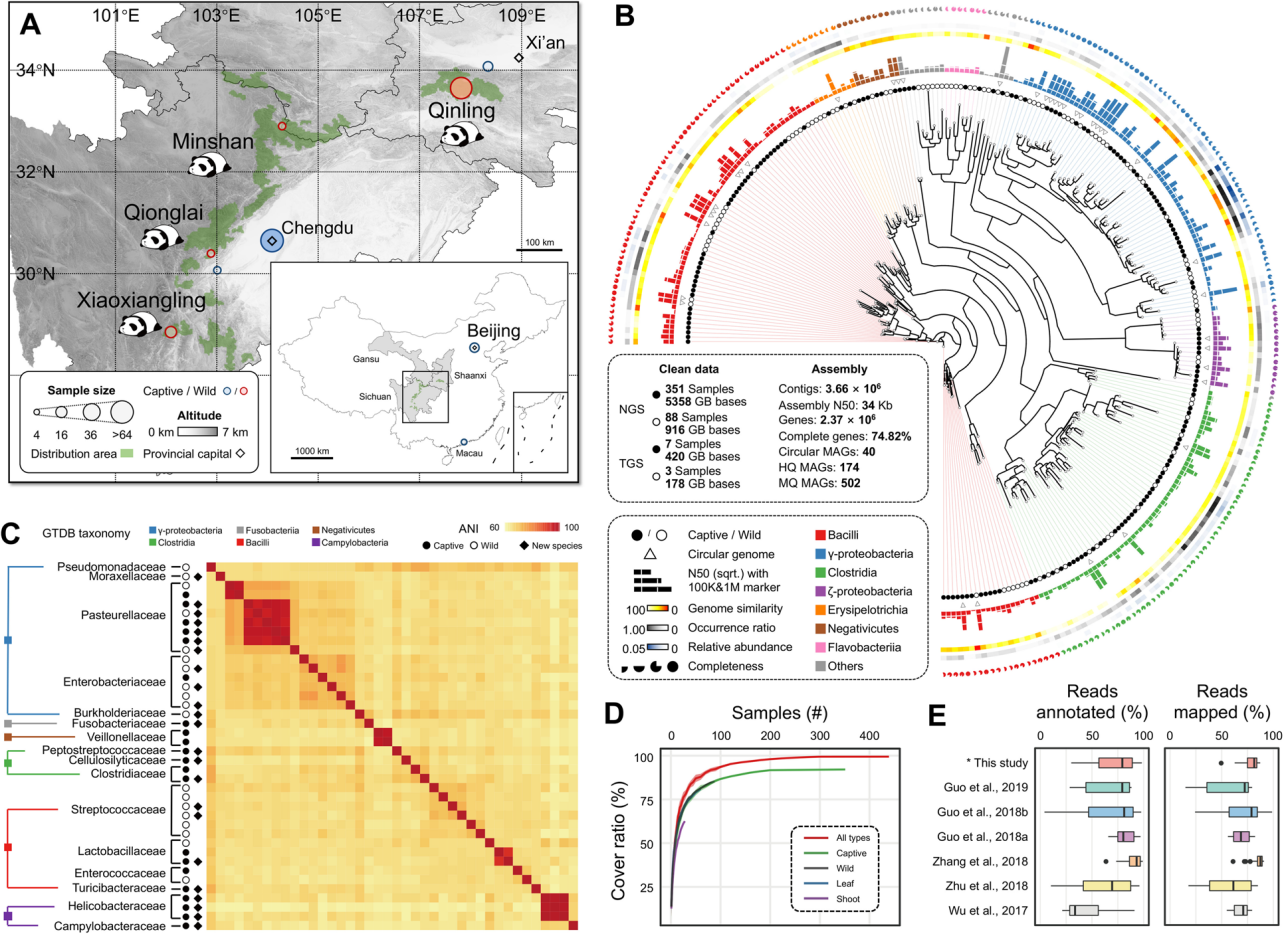
Construction and assessment of the pandaGUT database quality and representation. **A** Geographic distribution of the 439 giant panda stool samples collected from across all three wild genetic populations and most captive populations, with individuals spanning different dietary regimes, ages, sexes, and geographic distributions. **B** Maximum-likelihood tree constructed using at least 25 concatenated protein sequences from 469 medium- and high-quality metagenome-assembled genomes (MAGs). Clades are colored according to taxonomic class. From the inner circle to the outer circle, genome characteristics are indicated successively including sample source, circular genomes, N50 of contigs, genome similarity, genome occurrence ratio, and genome completeness. The solid and empty circles correspond to samples from captive and wild giant pandas, respectively. The 40 circular MAGs are indicated by triangles. In the outer layers, genome information (contig N50, genome similarity, and completeness), the occurrence ratio, and average relative abundance in all samples for each MAG are presented, respectively. **C** Average nucleotide identity (ANI) heatmaps for the 40 circularized MAGs. Taxonomic information is shown on the left. The solid and empty circles correspond to samples of captive and wild giant pandas, respectively. Diamonds represent 17 new species based on a < 95% nucleotide identity threshold. **D** Rarefaction curves depicting the coverage ratios of sequencing reads in the indicated samples against all sequence reads in all investigated samples. The number of unique identified genes finally reached a saturated state with increasing sample numbers, suggesting complete coverage of the gene catalog. **E** Percentage of mapping rates (left) and annotation rates (right) of de novo gene predictions generated in this study (as indicated by *) and for previously published giant panda gut metagenome datasets in comparison against the reference catalog pandaGUT

To obtain a comprehensive reference gene catalog and genome collection for panda gut microbiomes, Nanopore, PacBio, and Illumina sequencing data for 439 faecal samples from 131 individuals were integrated together (Fig. 1A, Fig. S1, Table S1). A total of 6.27 Tbp of short reads and 598 Gbp of long reads were used for the overall metagenome assemblies (Fig. S2), yielding a total of 3.66 × 10^6^ contigs with an overall assembly N50 of 34 kbp. After contig binning, refining, and decontamination, 820 metagenome-assembled-genome bins (MAGs) with summed length > 150 kbp were obtained (Table S2). Genome quality assessment with CheckM revealed that 502 MAGs met the medium-quality (MQ) criteria of ≥ 50% genome completeness and < 10% contamination, with 174 MAGs reaching the high-quality (HQ) criteria of > 90% genome completeness and < 5% contamination (Fig. 1B, Fig. S3A, B). Of the 174 HQ MAGs, 69 genomes possessed the 5S, 16S, and 23S rRNA genes together with at least 18 of the typical tRNAs (Table S2), further satisfying the “high-quality” criteria for MAGs set by the Genomic Standards Consortium [21]. Over 64.5% of the MQ MAGs were not assigned to species level against the Genome Taxonomy Database (GTDB), indicating a substantial abundance of potentially novel species in the pandaGUT dataset (Table S2).

The genome collection was dominated by large numbers of genomes from Firmicutes (*n* = 277) and Proteobacteria (*n* = 152), with Bacilli, γ-Proteobacteria, and Clostridia being the dominant classes (Fig. 1B, Table S2). The genome collection also contained many genomes from the Campylobacterota (*n* = 29), Bacteroidetes (*n* = 28), Actinobacteria (*n* = 12), and Fusobacteria (*n* = 4), reflecting the diversity of reference genomes in the catalog. Of the 502 MQ MAGs, 501 were classified into 47 families, 477 were classified into 88 genera, 179 were classified into 86 known species, and over 64% were not classified at the species level (Table S2). Of the genomes classified at the species level, *Escherichia coli* and *Clostridium cuniculi* were the most prevalent among samples (in 67.5% and 66.7% of the samples, respectively) and exhibited higher relative abundances (Fig. 1B). The bacterial genus most represented in the collection was *Clostridium*, with 59 MAGs (Table S2). Seven genomes were obtained that belonged to a previously unknown species and shared 87.35–94.4% ANI with *Clostridium sartagoforme*, a bacterium that can directly convert cellulosic biomass to biohydrogen in cows [22], potentially pointing to a gut bacterium that could provide alternative mechanisms for cellulose degradation. All high-quality MAGs possessed genes that largely were involved in metabolic pathways, including glycoside hydrolases (GHs) and glycosyl transferases (GTs), such as GH23, GT2, and GT4 (Fig. S4).

Notably, 40 MAGs in our dataset were circularized (Fig. 1B, Table S3 and Supplementary Dataset 1), with 23 of these not having been previously described (Fig. 1C). Using a pairwise ANI estimation cutoff of 95%, these 23 MAGs were classified into 17 new species belonging to five classes including the γ-Proteobacteria (*n* = 7), Bacilli (*n* = 4), Clostridia (*n* = 3), Campylobacteria (*n* = 2), and Fusobacteriia (*n* = 1) (Fig. 1C). The 40 circularized MAGs were annotated against the CAZy database and comprised a total of 1833 proteins predicted to be involved in carbohydrate metabolism, primarily comprising GHs (*n* = 816) and GTs (*n* = 757) (Table S4). However, relatively few proteins were classified as being involved in auxiliary activities (AA, 5) and polysaccharide lyase (PL, 24) classes. *Buttiauxella* sp. (MAG0005) contributed the largest number of proteins (*n* = 125), especially in the GH (*n* = 65) and GT (*n* = 43) classes, suggesting these populations are involved in cellulolytic activity. We also identified one strain of *Lactococcus lactis* (MAG0012) that is a well-known lactic acid producing bacteria [23].

One of the 40 circular genomes was classified as a novel species of the *Morganella* genus (MAG0010), with its 16S rRNA gene sharing ~ 96.4% nucleotide similarity with the closest relative, *Morganella morganii*. To improve taxonomic annotation, we cultured and purified a *Morganella* bacterial isolate from fecal samples and conducted de novo sequencing to obtain its complete genome. Genome sequence comparison between the MAG and the isolate genome revealed highly conserved synteny, with 98.07% ANI shared between the two genomes and over 99% shared nucleotide identity of 16S rRNA genes (Fig. S3C). Both short and long sequencing reads were then aligned against the genome of *M. morganii*, revealing that minimum coverage was > 10 × , and no gaps were present in the MAG (Fig. S3D). These results provide further evidence of the generally high quality and accuracy of the assembled genomes.

Five circular MAGs of the *Basfia* genus were obtained from different samples of wild and captive pandas and these MAGs shared 94.6–97.6% ANI along with highly conserved genomic synteny with each other (Fig. S5), suggesting similar taxonomic origins. We speculated that the strain-level genome variants might be attributed to different geographically distributed populations. In contrast, profound genome variation was observed between the five *Basfia* MAGs and the only known species of this genus, *Basfia succiniciproducens*, which is a succinic acid-producing bacterium that was isolated from cow rumen that can hydrolyse cellulose [24]. ANI comparisons were only < 76%, suggesting the presence of novel *Basfia* species involved in the degradation of fibre-rich bamboo that is part of the giant panda diet. These results highlight the potential for this catalog to expand our understanding of microbiome diversity of understudied taxa present within the panda microbiome.

### Integrated gene catalog of the gut microbiome and the representativeness assessment

The comprehensive pandaGUT gene catalog not only contributes to a more precise taxonomic identification of microbiome populations, but also helps predict functional profiles among hosts. A non-redundant microbial gene catalog was generated containing 2.37 × 10^6^ genes with an average length of 831.8 bp (Fig. S6), 74.82% of which possessed complete open reading frames (ORFs). To evaluate the gene catalog completeness, sequencing reads were mapped to it. Rarefaction curves reached saturated states with accumulated sequencing data, suggesting complete coverage of the gene catalog via the sequence effort used here (Fig. 1D). The classification rates of the gene catalog were also compared against those of several public datasets, including our previously published dataset [17], data from 16 XXL wild panda samples [25], data from seven QIO wild panda samples [26], data from 57 CDB captive panda samples [18], data from six CRC captive panda samples [27], and data from four captive panda individuals [28]. The classification rate ranged from 33.68 to 93.22% and increased with the read mapping rate (Fig. 1E, Fig. S6C). Although the captive panda dataset exhibited a marginal increase in classification, the rates of classification and mapping of each dataset were far from saturated (Fig. 1D). Thus, the integrated gene catalog outperformed all previous studies with respect to dataset coverage and substantially improved the detection of microbial genes.

To obtain an overview of functions in pandaGUT, genes were annotated against the COG database. Nearly half (1.08 × 10^6^) of the genes exhibited significant matches to the COG database (Table S5), indicating that a marked proportion of genes in the giant panda gut microbiome are still not functionally characterized. In terms of COG function distributions, 44.1% of genes were assigned to metabolic functions, with the most represented categories related to amino acid transport and metabolism, carbohydrate transport and metabolism, and transcription (Table S5). Thus, the integrated gene catalog from this study will serve as an important reference resource for the unified analysis of giant panda gut microbiomes.

### Host genetic-specific gut microbial diversity and functions in wild giant panda indicates host-gut microbiota phylosymbiosis

**Fig. 2:**
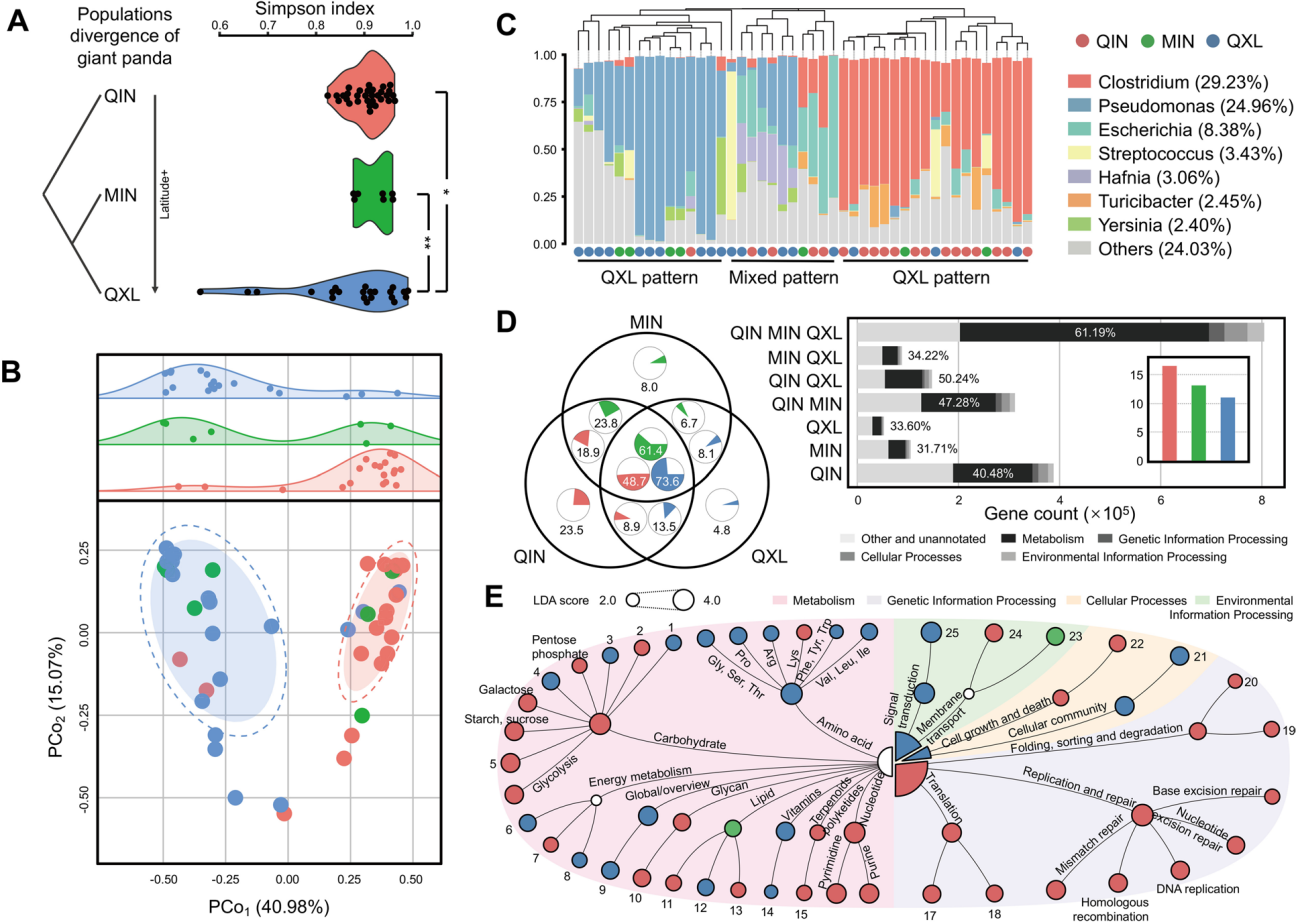
Host genetic-specific associated with the compositions and functions of wild giant panda gut microbiomes. **A** Alpha diversity of gut microbiomes among the three wild genetic populations (*n* = 45). The FDR-corrected Wilcoxon rank sum test was used to determine significance. **p < 0.01, *p < 0.05. The following group colors are the same as in **A**. **B** Principal coordinates analysis revealed two distinct clusters of microbial communities along the first principal component belonging to the QIN and QXL populations, respectively, with MIN communities exhibiting mixed compositions. The solid and empty ellipses were constructed based on multivariate normal distributions at 50% and 70% confidence levels, respectively. **C** Bar plot showing the assemblage patterns of giant panda gut microbiota at the genus level. Patterns were determined based on hierarchical clustering. **D** Characterization of host genetic-specific genes and KEGG pathways of gut microbiomes. Venn diagrams showing shared and unique genes in the gut microbiomes of each genetic population. The bar plot shows the gene counts at the second KEGG pathway level for each pairwise comparison. The inset bar graph shows the total numbers of microbial genes in each population annotated to KEGG orthologs. **E** Hierarchy of KEGG pathways showing the functional differentiation of gut microbiomes among the three genetic populations based on LEfSe analysis. Pathways with LDA > 2 and *p* < 0.05 are shown. The numbers refer to pathways designated in Fig. S8A

To identify the effects of host genetics on the diversity and functional repertoire of wild giant panda gut microbiomes and exclude the influences of diet, 45 representative samples across the three wild genetic populations were collected during foraging of similar food resources and then compared. Alpha diversity analysis based on the Simpson index revealed that the gut microbiomes of QXL populations possessed the highest diversity, while the QIN population microbiomes were the most divergent and their microbiomes exhibited the lowest alpha diversity (*p*_*FDR*_ < 0.05 and *p*_*FDR*_ < 0.01, respectively; Fig. 2A), consistent with the host genetic diversity patterns [15]. The Shannon index also exhibited similar trends among the three populations (Fig. S7A). We further determined intra- and inter-group variation in gut microbiomes based on Bray–Curtis dissimilarity values. The QIN and QXL populations exhibited significantly greater dissimilarity values (*p*_*FDR*_ < 0.001, Fig. S7B). Principal coordinates analysis (PCoA) further confirmed the presence of two distinct clusters corresponding to the QIN and QXL populations, respectively, with the MIN populations reflecting a mixed pattern along the first principal component that explained 40.98% of all variance (Adonis analysis, *R*^2^ = 0.18, *p* < 0.01; Fig. 2B). Hierarchical clustering analysis also revealed a clear separation between both populations, while also accounting for the horseshoe effect, indicating the presence of niche differentiation along environmental gradients [29]. Moreover, the QIN populations exhibited significantly lower intragroup variance than the MIN and QXL groups (*p*_*FDR*_ < 0.001; Fig. S7B). Thus, the diversity of the gut microbiomes across the three genetic populations exhibited two distinct patterns overall that were associated with host genetic structures.

Two distinct compositional profiles were observed across the three genetic populations, with *Clostridium* (over 60% average relative abundance) and *Pseudomonas* (over 70% average relative abundance) representing the most dominant taxa (Fig. 2C). *Clostridium* was the dominant taxon in most of the QIN population pandas, while *Pseudomonas* was overrepresented in the QXL cohort, and the MIN cohort exhibited a mixture of *Clostridium* and *Pseudomonas*. Functional annotation of microbial genes within the three genetic populations revealed that QIN microbiomes possessed more annotated KOs (*n* = 1.65 × 10^6^), with 23.5% population-specific annotations that were three- to four-fold higher than in the MIN (8.0%) and QXL (4.8%) cohorts, respectively (Fig. 2D). Notably, genes related to genetic information processing were significantly enriched in the QIN cohort (LDA > 2, *p* < 0.05), including those associated with replication and repair in addition to nucleotide metabolism, including DNA replication, purine metabolism, pyrimidine metabolism, and the upstream pentose phosphate pathway (Fig. 2E, Fig. S8A).

We further reconstructed the DNA biosynthesis pathways of the gut microbiome based on significantly differentially abundant genes (*p*_*FDR*_ < 0.05) (Fig. S8B). The QIN populations exhibited significant enrichment of the enzymes EC3.2.1.122 and EC3.2.1.21 compared to the QXL cohorts, and these enzymes are involved in starch and sucrose metabolism pathways for hydrolysing polysaccharide compounds to glucose. Glucose is then phosphorylated to glucose-6-phosphate by EC2.7.1.2, while EC2.7.1.199 participates in the pentose phosphate pathway for the formation of phosphoribosyl pyrophosphate (PRPP) by the enzymes EC2.2.1.1, EC2.7.1.11, EC5.1.3.1, and EC5.3.1.6. Finally, PRPP is transformed to inosine monophosphate (IMP) by the enzymes EC 2.7.1.133, EC1.17.4.2, EC2.7.1.76, EC2.7.1.74, and EC2.7.1.21 (Fig. S8B). IMP is an important precursor for the biosynthesis of purine and pyrimidine nucleotides. The enrichment of DNA repair- and replication-related pathways in the QIN gut microbiome suggest that gene plasticity could confer the host with a plastic response of degradation functions based on seasonal dietary shifts. Taken together, these results reveal host genetic-specific gut microbial structures and functions, providing robust evidence to support the co-evolution between giant pandas and their gut symbionts.

### Strain-level variation in *Clostridium* drives functional changes in wild giant panda gut microbiome for adaptation to seasonal dietary changes

**Fig. 3:**
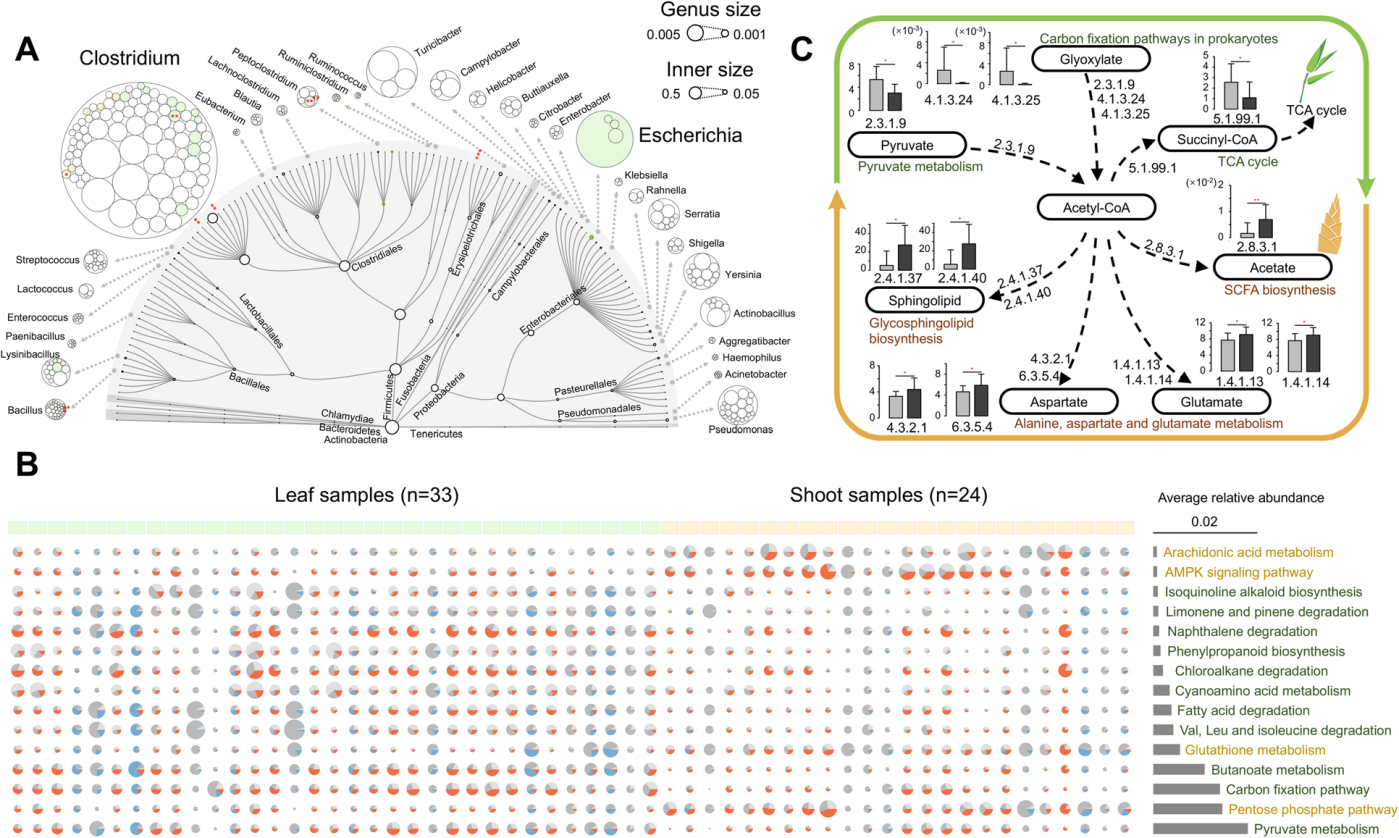
Strain-level analysis of variation in gut microbial composition and functional profiles of QIN giant pandas between leaf and shoot eating seasons. **A** The variation in composition and abundance in the gut microbiota of giant pandas (*n* = 57) at the genus and species levels. Significantly differentially abundant taxa between seasons are indicated by asterisks. The size of the circle indicates the abundances and colors indicate the abundances of taxa that significantly differed between leaf- (green) and shoot-eating (orange) seasons. **B** The 15 most differentially abundant KEGG pathways and quantitative contributions to functional profiles that belonged to *Clostridium* (orange), *Escherichia* (blue), and other taxa (grey). The size of the circle indicates KEGG pathway relative abundances and the area of the sector indicates the relative contributions of each taxon. The color outlines indicate that the KEGG pathways significantly differed between the leaf- (green) and shoot-eating (orange) seasons. **C** Reconstruction of acyl chain-associated pathways that were differentially enriched between samples from different seasons. Histogram colors indicate genes that significantly differed in communities collected between leaf- (gray) and shoot-eating (black) seasons. FDR-corrected Wilcoxon rank sum tests were used to determine significance. **p < 0.01, *p < 0.05

How gut microbial composition and function adapt to host food seasonality in natural environment has rarely been investigated, particularly at single-nucleotide polymorphisms (SNPs) level. The QIN cohort of pandas exhibited an apparently seasonal diet of bamboo leaves and shoots, resulting in seasonal variation in microbial functional potentials [30]. However, the SNPs within functional genes that varied between seasons remain poorly classified. Six species exhibited significantly different relative abundances between seasons (Fig. 3A), including *Clostridium* sp. K25 (*p*_*FDR*_ = 0.034), *Clostridium algidicarnis* (*p*_*FDR*_ = 0.045), *Clostridium hiranonis* (*p*_*FDR*_ = 0.045), and *Clostridium cellulosi* (*p*_*FDR*_ = 0.045). The SNP profiles of cellulose-degrading genes like cellulase (*cel*), beta-glucosidase (*bglX*), and xylan 1,4-beta-xylosidase (*xynB*) were then evaluated in the genomes of six *Clostridium* species. Interestingly, seasonally distinct characters were observed for SNP abundances and read mapping rates, since the genes during the leaf eating season exhibited significantly higher polymorphisms and abundances, including especially *bglX* (*p*_*FDR*_ < 0.05; Fig. S9), suggesting that strain-level variation in *Clostridium* may contribute to cellulose degradation.

Differences in KEGG pathway enrichment was then compared between seasons. Among the most overrepresented pathways with relative abundances higher than 0.05%, 15 exhibited significantly different enrichments between seasons (*p* < 0.05, FDR < 0.1; Fig. 3B). Four pathways were notably enriched in the shoot eating season, including arachidonic acid metabolism and the AMPK signaling pathway, which are both involved in fatty acid metabolism. In contrast, pyruvate metabolism and carbon fixation pathways of prokaryotes were enriched in the leaf eating season microbiomes and may contribute to providing acyl chains for the de novo synthesis of essential nutraceuticals (Fig. 3C). To further determine the contributions of the overrepresented taxa *Clostridium* and *Escherichia,* in addition to other taxa, towards differentially abundant pathways, metabolic genes were taxonomically classified. An overwhelming contribution of *Clostridium* to metabolic activities was observed in the microbiomes from either the leaf or shoot eating seasons, thereby providing further evidence that genetic polymorphisms of *Clostridium* drive functional plasticity in response to seasonal dietary changes.

### Enterotype and resistome landscapes of the wild and captive giant panda gut microbiomes inform conservation implications

**Fig. 4:**
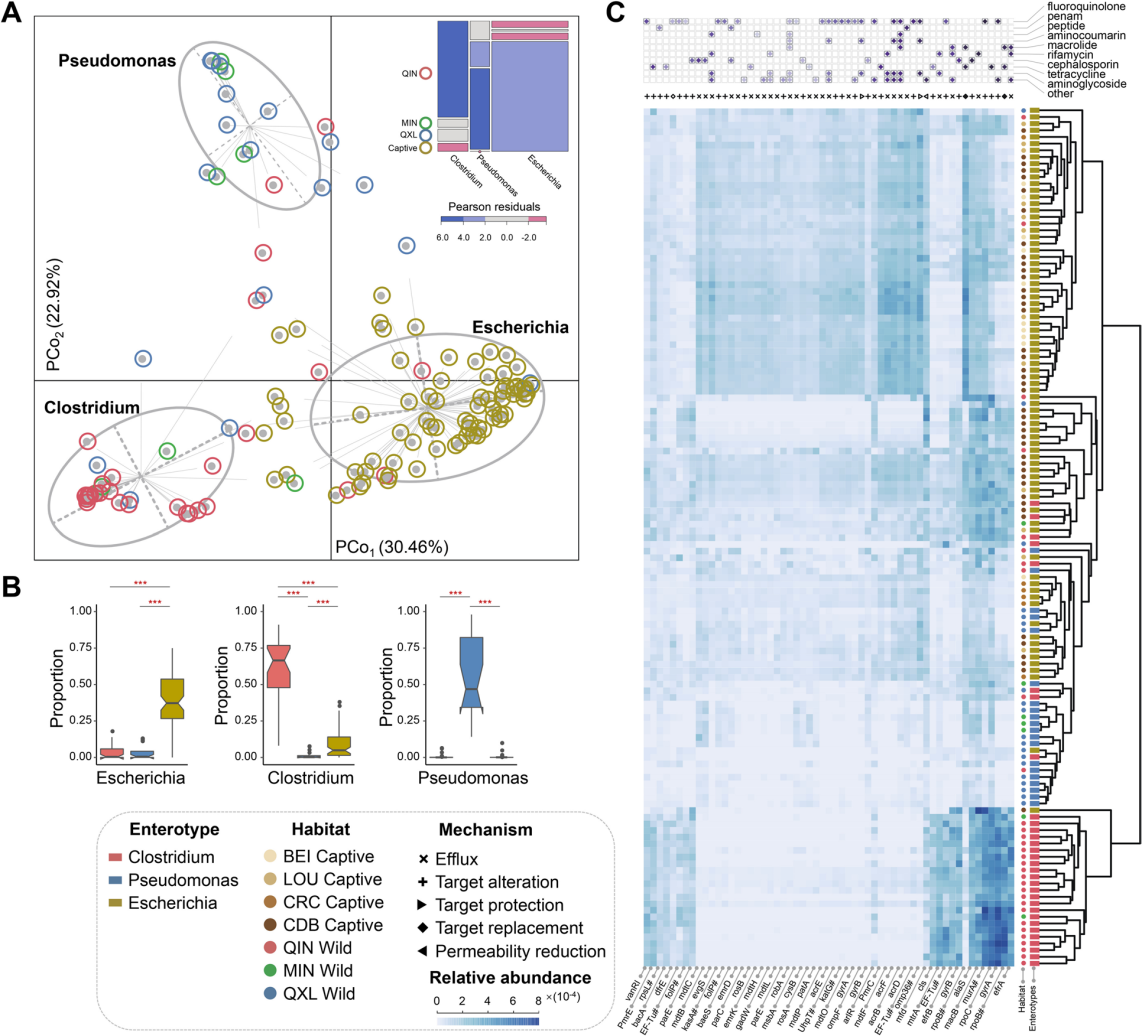
Characteristics of gut microbiome enterotypes and resistomes across wild and captive giant panda populations. **A** PCoA plot revealing three clusters in the gut microbiome, with each being significantly identifiable by variation in the relative abundances of *Clostridium*, *Pseudomonas*, and *Escherichia*. The lines connected to the center of each ellipse correspond to the affiliation. The inset mosaic plot shows the association between giant panda cohorts and enterotypes. The Pearson residuals were used to assess the individual contribution to the Pearson statistic. The blue, red, and grey colours correspond to positive, negative, and lack of associations, respectively. The area of each plot represents the sample size of each group. **B** The box plots show the relative abundances of major bacterial contributors of each enterotype. FDR-corrected Wilcoxon rank sum tests were used to determine statistical significance. ***p < 0.001, **p < 0.01, *p < 0.05. **C** Heatmap of antibiotic resistance genes (ARGs) identified among the three enterotypes. Information regarding resistance mechanisms and antibiotics is highlighted at the top. Color intensity indicates ARG abundances and darker colors indicate higher abundances. The sources and corresponding enterotypes of the sample in each line are marked in the tree (right)

To characterize the differences in gut microbiome compositions between wild and captive giant pandas, the samples encompassed all the three wild genetic populations and four different captive cohorts, with complete sample information were used for downstream analyses. Microbial profiling resulted in the identification of three clusters (termed enterotypes) present in the wild (*n* = 59) and captive (*n* = 73) pandas (Fig. S10A), with each being significantly identifiable by variation in the relative abundances of *Clostridium*, *Pseudomonas*, and *Escherichia* (*p*_*FDR*_ < 0.001; Fig. 4A, B). Correlation analysis of the giant panda cohorts and the enterotypes indicated that the QIN wild pandas belonged to the *Clostridium* enterotype and the QXL wild pandas belonged to the *Pseudomonas* enterotype, whereas all the captive cohorts from different geographic distributions belonged to the *Escherichia* enterotype (Fig. 4A), indicating that captivity significantly alters natural microbial profiles. In addition, the *Escherichia* enterotype also exhibited more abundant *Streptococcus* than the other two enterotypes (Fig. S10B). These results suggest that enterotype provides potential implications for future conservation translocation.

To further monitor the prevalence of antibiotic resistance genes (ARGs) in panda gut microbiomes that may lead to alterations in microbial compositions, a Comprehensive Antibiotic Resistance Database (CARD) annotation scheme was used to capture the full breadth of the resistomes. A total of 752 ARGs associated with five antibiotic resistance mechanisms were identified in gut microbiomes across all enterotypes. ARG distributions exhibited high heterogeneity among the three enterotypes (Fig. 4C). The *Escherichia* enterotype exhibited the highest abundances of ARGs, with efflux pump genes (e.g., *acrB*, *acrF*, *robA*, and *evgS*) being particularly prominent and conferring resistance to at least three antibiotics, suggesting a potential risk of multidrug resistance. The *Pseudomonas* enterotype exhibited the lowest abundances of ARGs, while the *Clostridium* enterotype exhibited moderate abundances. Analysis of the 502 microbial genomes also confirmed the enterotype-related resistome profiles (Fig. S10C). Correlational analysis indicated that enterotype displayed higher correlation with resistomes than captivity status (i.e., wild or captive), geographic distributions, and diets (Fig. S10D, E), suggesting that resistomes significantly altered the enterotypes of the captive cohort, in contrast to their wild counterparts.

Integrative and conjugative elements (ICEs) broadly refer to a collection of mobile genetic elements that participate in the important process of horizontal transfer of antibiotic resistance among various bacterial species [31]. A novel ICE containing a tetracycline resistance gene *tet*(M) was identified in four MAGs that were assembled from the four different captive panda samples and these MAGs were classified as *Veillonella parvula* and *Streptococcus* sp. 002300045 (Fig. S10F). The genetic locus of the *tet*(M) gene, in addition to its flanking regions were analyzed to investigate their mobility potential. Genes encoding enzymes involved in bacterial conjugation were found flanking the *tet*(M) gene, including *nick* (relaxase), *YddH* (hydrolase), and *ydcQ* (encoding a membrane vesicle protein). In addition, genes encoding a putative genome engineering toolkit were also detected in the flanking sequences, including *recX* that encodes a single-stranded DNA-binding protein that can catalyze homologous recombination, in addition to *xis* that encodes excisionase, and *int* that encodes an integrase. In addition, accessory proteins commonly associated with mobile elements, like *tnpA* and *tnpR* that encoded transposons, were also found flanking the *tet*(M) gene. These results suggest that the *tet*(M)-containing ICE may mediate horizontal gene transfer (HGT) among members of the gut microbial communities of captive pandas. These results provide important baselines of ARGs in captive pandas as well as the natural populations.

## Discussion

Mapping wildlife microbiomes can guide conservation efforts. In this study, we constructed a comprehensive catalog of giant panda gut microbiomes, resulting in the pandaGUT database that comprises 2.37 million genes and 502 better-quality MAGs, 64.5% of which have not been previously described. The 40 complete bacterial genomes obtained in this study expand the coverage of several lineages and phylogenetic diversity within the panda gut microbiome, also expanding prokaryotic genomic representation. Three gut microbiome enterotypes were identified using the data that spans different genetic populations, dietary regimes, ages, sexes, and geographic distributions. We found that wild pandas exhibited host genetic-specific microbial structures and functions, whereas the captive counterparts exhibited much higher abundances of multi-antibiotic resistance genes. Our findings have an importance significance for science-informed conservation.

### Comprehensive landscapes of gut microbiome diversity and functions for the threatened mammal exhibit several advances over previous studies of pandas and other wildlife

Reference gene catalogs and metagenome-assembled genome collections of microbiomes from several model organisms, including humans [32–40], non-human primates [41], mice [42–44], pigs [45, 46], dogs [47], chickens [48], and ruminants [49–54]; however, it still remains lacking for the threatened mammal. This study exhibits several unique advances over previous gut microbiome studies of pandas and other wildlife. First, this study encompasses the varied sample sources of giant panda and an unprecedented level of large-scale metagenomic data that was obtained by combined Nanopore and PacBio long-read and Illumina short-read sequencing. These efforts enabled recovery of more complete microbial genomes and increased catalog representation. Second, contig assembly qualities were markedly higher in this study, relative to previous studies, resulting in an overall contig N50 of 34 kbp, representing a 4.5–27-fold increase over values obtained in previous studies [26]. Consequently, 74.82% of the microbial genes were identified as complete, representing a much higher number than values previously obtained for ruminants (32.2% [52]) and humans (57.74% [34]). Third, rRNA genes have previously been shown to be problematic for inclusion in MAG assemblies with short-read metagenomic data [55]. The combination of short-read and long-read sequencing technologies that were used here, in addition to assemblies from different samples resulted in overcoming this issue to a significant extent. In particular, this study recovered a much higher number of high-quality MAGs encoding 5S, 16S, and 23S rRNA genes in addition to tRNAs. Fourth, the genome catalog covers many low-abundance species that were neglected in previous studies [20, 30]. Low-abundance bacteria like C. sartagoforme and Basfia strains that were represented in this dataset may exhibit important biochemical roles in fibre degradation, representing promising resources for future biotechnological applications.

### Unique signatures of QIN panda gut microbiome with host genetic-specific and seasonal variations indicate adaptive evolution

A strong phylosymbiosis signal of host–microbiome coevolution has been previously shown across many animal clades [56]. Giant pandas belong to the order Carnivora and have evolved to exclusively feed on bamboos that are rich in cellulose and hemicellulose. Host genetic-specific gut microbiome structures and functions were observed here for pandas. The QIN pandas exhibited lower genetic diversity [15], but also lower gut microbial community diversity. Nevertheless, the QIN panda communities harbored the most abundant metabolic genes that may then serve as a significant reservoir of functional diversity. We speculate that such functional diversity may be related to seasonal variation of food sources in the Qinling Mountains. Strain-level analysis of SNPs within the cellulose-degrading genes of *Clostridium* further indicated a plastic microbiome response to variation in dietary nutrients.

The population density of QIN panda is higher than those of other populations, which may result in overlap in home range. However, considering the solitary nature of giant panda, the social interaction may have less effect on the gut microbiome composition, which may be distinct to that of non-human primates [57, 58]. This effect is needed to be examined in the future. Considering the distinctiveness of the QIN cohort via lower genetic diversity, but also lower gut microbial diversity and unique microbiome enterotypes, we suggest that a national park should be specifically established for QIN pandas. In addition, key gut bacterial populations within the QIN microbiomes that exhibit important metabolic activities should be cultured and functionally characterized. Such efforts would represent an important step towards manipulating gut microbiomes for improving giant panda fitness in the future.

### Gut microbiome structure divergence and reducing antibiotic usage should be highlighted in future conservation translocation

Antibiotic exposure can strongly induce the enrichment of ARG-containing pathogens [59, 60], and this represents a significant threat to the overall health of wildlife and the public, while also resulting in giant panda enterotype variation. In this study, captive giant panda gut microbiomes consistently harboured diverse ARGs that were dominated by bacterial populations conferring specific resistance to multiple antibiotics. ARGs were also notably identified in the gut microbiomes of wild populations, most likely due to the selection pressures from natural antibiotic secretion by gut bacteria or environmental bacteria over the long evolutionary timescales. Moreover, the presence of ICEs containing tetracycline resistance genes suggests potential dissemination of antibiotic resistance through HGT that would further adversely affect conservation management and public health [61]. We consequently recommend reducing antibiotic usage for wildlife, while regularly monitoring the ARG content of their gut microbiomes. Considering that the divergence in the enterotypes between wild and captive pandas, we further suggest that rational manipulation of captive gut microbiomes to better reflect those of their wild counterparts to improve the capacity for ecological adaptation of wildlife. The recently proposed enterosignature concept can not only confirm key ecological characteristics of gut microbiome, but also enable to detect the gradual shifts in community structures [62], which should be introduced into wildlife conservation reintroduction. More longitudinal data should be collected and assessed in the future to further test the variation of the microbial community structures in giant panda.

In summary, this study offers a comprehensive and unprecedented catalog of the gut microbiome of a threatened mammal and reveals three enterotypes that are associated with host genetics and captivity. These findings have far-reaching implications for future metagenome-based evolutionary studies and conservation of giant pandas and other wildlife.

## Methods

### Sample collection and metagenomic data retrieval

To comprehensively obtain faecal samples from wild giant panda populations, teams were assembled in different nature reserves and sample collection was performed by trained laboratory staff with assistance of reserve staff. Line transects used for sample collection had been widely used in wild giant panda population census surveys, ensuring the probability of obtaining fresh faces. De novo generation of fecal metagenomic data was conducted from 111 fresh stool samples that were collected from 76 individuals during the leaf and shoot eating seasons (Table S1). Thirty-eight out of the 111 stool samples were collected from the wild cohort in the QIN (*n* = 31 stool samples) and MIN (*n* = 7) mountains. The remaining samples were collected from captive cohorts in the Beijing Zoo (BEI, *n* = 10), the Chengdu Research Base of Giant Panda Breeding (CDB, *n* = 51), and the Shaanxi Rare Wildlife Rescue and Feeding Research Centre (SXC, *n* = 12). The surfaces of fresh faeces were removed. All stool samples were then immediately flash-frozen in liquid nitrogen after collection and maintained under anerobic conditions, followed by transfer to the laboratory on dry ice, and storage at − 80 °C until later use. All samples were collected based on the criteria needed to meet Nanopore or PacBio sequencing thresholds. Stool DNA extraction and metagenome sequencing were conducted using the same standardized protocols. All sample collection procedures were approved by the Institutional Animal Care and Use Committee of the Institute of Zoology, Chinese Academy of Sciences (Beijing, China).

In addition, 328 publicly available metagenomic datasets were collected from 55 giant pandas and combined for integrative analysis, including the 26 stool samples collected from QIN in our previous study [17] and data from five other metagenomic studies, including samples from the QIO (*n* = 7) [26] and XXL (*n* = 16) [25] wild cohorts, in addition to those from the CDB captive cohort (*n* = 57) [18], the China Conservation and Research Center for Giant Pandas [27], and from four individuals at the Macao Giant Panda Pavilion [28].

### DNA extraction and sequencing

To prepare samples for molecular analyses, cecal samples (1 g per sample) were homogenized and passed through a 100-μm filter membrane to remove bamboo fibres. Stool DNA was then extracted using the QIAamp Power Fecal DNA Kit (Qiagen, Germany) following the manufacturer’s instructions. DNA concentration and purity were assessed using a NanoDrop2000 spectrophotometer (Thermo Fisher Scientific Inc., USA), and the quality of the extracted DNA was evaluated with electrophoresis on an 0.8% agarose gel. High-quality DNA was then used to construct a metagenomic library with an insert size of 500 bp, followed by sequencing on the Illumina NovaSeq platform (150 bp paired-end reads) to generate at least 12 Gbp of raw data per sample. DNA extracted from each sample from one of nine groups was pooled for Nanopore sequencing (Fig. S1, Table S1). Another DNA pool from the leaf and shoot groups of the CDB samples were also subjected to PacBio Sequel sequencing.

### Metagenome assembly, genome binning, and quality assessment

To obtain high-quality metagenomic data, sequencing data were mapped to the giant panda, bamboo, and human genomes using bowtie2 v.2.4.0 [63] to remove potential DNA contamination. The bamboo genome used for this analysis was the sequence for the species closest to the panda’s food that was available in the NCBI database. Considering that de novo discovery of non-bacterial genomes is very challenging but should receive more attention in the future, the reads of eukaryotic microorganisms, archaea, and viruses were excluded from the following analysis. All the reads that were assigned to eukaryotic microorganisms, archaea, or viruses by kraken2 or minimap2, respectively, were removed. Following quality trimming of Illumina short reads using sickle v.1.33 (https://github.com/najoshi/sickle), the sequencing reads of each sample were individually assembled into contigs using Megahit v.1.1.3 [64] (parameters: –min-count 2 –k-min 27 –k-max 87 –kstep 10 –min-contig-len 500). High-quality reads within the same groups were also co-assembled to obtain co-assemblies using Megahit v.1.1.3 (Fig. S1).

The clean reads from Nanopore and PacBio sequencing were obtained after removing chimaeric and adaptor sequences using Porechop v.0.2.4 (https://github.com/rrwick/Porechop). The low-quality and short subreads were also filtered using Nanofilt v.2.6.0 [65] and Filtlong v.0.2.0 (https://github.com/rrwick/Filtlong). Long contigs were then generated using metaFlye v.2.7 [66], and the Racon v.1.4.10 [67] program was used to correct base errors within the long read sequences.

To further reduce errors generated in the assembly, all short- and long-reads were mapped back to the assembled contigs using bowtie2 v.2.4.0 [63] and Samtools v.1.10 [68] to correct single bases, insertions, and deletions [69]. Following short- and long-read correction, contigs generated with Megahit and metaFlye assemblers from the same group were merged together with quickmerge v.0.3 (https://github.com/mahulchak/quickmerge) and further polished five times with Pilon v.1.23 [70] and Nextpolish v.1.2.4 [71]. All the assembled contigs that were assigned to non-bacterial clades were removed.

Genome binning into metagenome-assembled-genomes (MAGs) was then conducted using MaxBin v.2.2.4 [72], MetaBAT2 v.2.11.1 [73], CONCOCT v.0.4.0 [74], and VAMB v.3.0.3 (https://github.com/RasmussenLab/vamb), resulting in 1047, 2335, 1344, and 1750 genome bins, respectively. The DAS Tool v.1.1.1 [75] program was then used to integrate bins generated from the different methods and ensure representativeness and diversity of all MAGs. The integrated bins were further decontaminated and merged by aligning all of them to the core genes of the corresponding genus or family using a greedy approach to filter bins. When removing contaminated contigs, bins that had completeness values decrease were then removed.

The completeness and contamination of the final bins were determined using CheckM v.1.0.11 [76]. Bins with estimated genome completeness > 90% and contamination < 5% were considered high-quality MAGs and bins with completeness ≥ 50% and contamination < 10% were considered medium-quality MAGs, as described elsewhere [21].

To evaluate circularity and precisely locate the genome wrap-around point in single-contig genomes, we determined whether redundant sequences were present at the wrap-around point of the genome contigs, as previously described [69]. We also collected reads that aligned to the termini of a candidate genome and assembled the spanning contigs for alignment. In addition, the Illumina, Nanopore, and PacBio reads were further mapped to the circular genomes to assess whether chimeric regions were present.

The circular genomes were taxonomically classified using GTDB-Tk v.0.3.2 [77] with a cut-off of ≥ 95% ANI and by using FastANI v.1.2 with the default options. The genomic similarity of each genome bin was calculated as the ratio of the shared length to the total length of the bin following alignment to representative genomes in the GenBank database (October, 2020) with Mash [78]. The Illumina sequencing reads of each sample that covered at least 50% of the genomic bin was used to identify the presence of the bin in that sample, and the percentage of the samples where a bin was identified was considered the occurrence ratio for the bin. A maximum-likelihood tree of the medium-quality MAGs was constructed using FastTree v. 2.1.9 [79] with at least 25 concatenated protein sequences [80].

### Construction of the gene catalog and comparison with previous datasets

ORFs were predicted using Prodigal v.2.6.3 [81] with the parameter “-p meta”. rRNAs were then annotated with RNAmmer v1.2 [82], and tRNAs were annotated with tRNAscan-SE v.2.0.9 [83]. ORFs < 100 bp in length were discarded, and the remaining ORFs were clustered using CD-HIT v.4.8.1 [84] by specifying the parameter settings of -n 10 -c 0.95 -G 0 -M 0 -aS 0.9.

To assess the representativeness of the gene catalog of the giant panda gut microbiome assembled here, the gene catalog was compared against all metagenomic datasets included in this study using 90% of protein sequence identity with CD-HIT v.4.8.1. The clean reads of each sample were also aligned to the gene catalog to calculate the coverage ratio and the numbers of mapped reads to the genes were determined using Samtools v.1.10. The proportion of mapped reads was calculated by computing the percentage of mapped reads to the total numbers of reads in each sample. Further, the annotation ratio of each sample was calculated as the percentage of annotated genes in the gene catalog of this study.

### Taxonomic annotation, functional annotation, and abundance analysis

All gene catalog members were subjected to taxonomic and functional assignment using DIAMOND v.0.9.22 [85] via comparison against the NCBI-NR (Oct 2020) and Kyoto Encyclopedia of Genes and Genomes (KEGG) databases using an *e* value cutoff of ≤ 1e − 5. Carbohydrate-active enzymes (CAZymes) were also annotated using the hmmscan function of HMMER v.3.2.1 [86] to identify protein sequences and their corresponding representatives in the CAZy database (v.7; http://www.cazy.org/) using an *e* value cutoff ≤ 1e − 5.

The relative abundances of taxa, KEGG orthologous groups (KOs) and CAZymes, were calculated based on the abundances of annotated genes. Gene abundances in each sample were estimated by mapping the quality-trimmed reads to the non-redundant gene catalog at the 95% nucleotide identity threshold using bowtie2 v.2.4.0 and Samtools v.1.10. The FeatureCounts v.2.0.1 [87] program was then used to quantify the numbers of successfully assigned reads. The abundances were normalized to fragments per kilobase of gene sequence per million reads mapped (FPKM) values, as previously described [88]. To establish taxonomic profiles, phylogenetic assignments of each annotated gene from the gene catalog were evaluated and the relative abundances of genes from the same taxon were summed to represent the abundance for the taxon. The profiles of each KO, KEGG pathway, CAZyme, and CAZyme family member were calculated using the same procedures. The limma and ComBat programs were used to minimize potential batch effects, as described previously [89].

Linear discriminant analysis (LDA) effect size (LEfSe) analysis was used to identify the key characteristics of KEGG pathways in the gut microbiomes of the three genetic populations. LDA scores > 2.0 and *p* < 0.05 were considered statistically significant.

The functional contributions of the most abundant bacteria in the QIN cohort between seasons, including *Clostridium*, *Escherichia*, and other genera, were assessed by mapping genes annotated in each of the metabolic pathways to the genomes of *Clostridium*, *Escherichia*, and other genera using DIAMOND [85].

### Gut microbiota diversity analysis

To analyze the alpha and beta diversity of the gut microbiota communities, the Shannon and Simpson indices were calculated using the vegan R package v.3.6.2, whereas Jensen–Shannon divergence (JSD) was estimated using the phyloseq R package. Further, PCoA based on Bray–Curtis distances between communities was performed using vegan. The expectation–maximization algorithm was used to estimate the subcomponent of a mixture Gaussian distribution for the first principal component of the beta-diversity analysis for wild samples. Two components were identified using the Bayesian information criterion.

### Cellulase-encoding genes in *Clostridium* and polymorphism analysis

All *Clostridium* contigs were aligned to sequences of known cellulase-encoding genes, including β-glucosidase (*bglX*), endo-β-1,4-glucanase (*celA*, *cel5A*), and xylan 1,4-β-xylosidase (*xynB*), with the thresholds of alignment length as > 100 bp, nucleotide identity > 70%, and sharing by at least 60% of hits. Strain-level haplotypes were generated using VCFtools v.0.1.17 (using the parameters: filter -O v -o -e 'QUAL < 30 || DP < 20'). SNP density was also determined by the ratio of filtered SNP bases to total mapped bases.

### Enterotype classification

Enterotype clustering was conducted at the genus level, as previously described [90, 91]. The genus with the highest relative abundance was considered the primary contributor to each enterotype. Samples were selected for enterotype clustering according to the following criteria. First, samples from overlapping sampling regions were avoided. If samples of the published studies were collected from a region that overlapped with the sampling region of this study (Table S1), the metagenome data for these samples were not included. Second, samples with incomplete host dietary information were excluded. A total of 132 representative samples remained and were clustered with the pam function of the cluster R package [90]. The optimal number of clusters was then selected according to the Calinski–Harabasz (CH) index.

### Resistome and ICE identification

ARGs were annotated against the CARD database using RGI v.5.1.1 [92]. The alignment parameters included a minimum sequence identity of 70%, a sequence length cutoff of 60%, and an *e* value < 1e − 6. UPGMA was used to construct a hierarchical cluster tree of the resistome, yielding six clusters using a cutoff of tree height = 0.0007. Variables that influenced enterotype classification were determined using Cramer’s V statistic. To assess the potential mechanisms of ARG mobility, ICE’s flanking resistance genes in the genomes of gut bacteria were investigated, as previously described [31]. Nucleotide sequences were aligned with ICEs available in the ICEberg database to identify potential ICEs using BLASTn (default parameters) using a length cutoff of > 5 kbp. The identified ICEs were then annotated against the CARD, ARG-ANNOT, and ResFinder databases. *tet*(M) was identified in five of the genomes and 15 kbp of the sequences flanking *tet*(M) were annotated using UniProt (https://www.UniProt.org/) and by specifying a minimum coverage threshold of 50%.

### Isolation and whole-genome sequencing of *Morganella morganii*

Fresh panda feces were collected from Beijing Zoo, Beijing, China, immediately after defecation. Fecal bacterial suspensions were prepared from the samples for cultivation using Columbia agar with 5% sheep’s blood (Qingdao Hope Bio-Technology Co., Ltd., Qingdao, China) and incubation at 37 °C for 16 h with a 5% CO_2_ atmosphere. Colony morphological analysis and whole-genome sequencing were performed for confirmation of the *M. morganii* identification. The complete genome sequence was then determined using the Illumina MiSeq paired-end (400 bp library) and Nanopore sequencing technology platforms.

### Statistical analyses

Comparison of the microbiomes of different genetic populations or from pandas with different foraging seasons was performed using Kruskal–Wallis tests (multiple-group comparison) or two-tailed Wilcoxon rank-sum tests (pairwise comparisons). A false discovery rate (FDR)-corrected *p* < 0.05 was considered statistically significant for comparing bacterial species, KEGG pathways, and MAGs. The results were visualized with boxplots or heatmaps plotted with the ggpubr and pheatmap packages in R v.3.6.2, respectively. Text processing, information extraction, and data statistics incorporated in the pipeline for construction of the pandaGUT database and MAGs were processed using R v.3.6.2.

### Supplementary Information

**Additional file 1: Fig S1.** Pipeline for the construction of the unified pandaGUT reference catalogue of the giant panda gut microbiome. Approximately 7 Tbp of long- and short-read sequencing data from diverse samples spanning different genetic backgrounds, dietary regimes, ages, sexes, and geographic distributions were integrated to construct the catalogue. pandaGUT contains 502 nonredundant MAGs and 2.37 million unique genes. **Fig S2.** Overview of sample and metagenome data integrated in the pandaGUT catalogue. (A) Metagenome statistics for *de novo* samples generated in this study and six other publicly available studies. The dark colours indicate the total numbers of the samples, and the light colours indicate the numbers of the samples contaminated by either > 1% human genome sequences, > 10% giant panda genome sequences, or > 1% bamboo genome sequences in the corresponding study. (B) Metagenome statistics for the metagenomic sequencing data *de novo* generated in this study and data from six other publicly available studies. The dashed square indicates Nanopore and PacBio sequencing data statistics. **Fig S3.** Genome quality of species representatives. (A) The completeness and contamination of all bins before (red colour) and after (green colour) refining by merging, splitting, and decontaminating. (B) GC content and MAG size statistics. (C) Synteny analysis (left) and statistics of genomic features (right) between the isolated and sequenced genome (ISG) and metagenome-assembled genome (MAG) of *Morganella morganii*. (D) Coverage of Nanopore and Illumina sequencing reads that were mapped to the *M. morganii *genome in addition to GC content distribution. **Fig S4.** Annotation information for the Clusters of Orthologous Genes (COG) and CAZyme genes within 174 high-quality metagenome-assembled genomes. AA, auxiliary activities. PL, polysaccharide lyase. GH, glycoside hydrolases. GT, glycosyl transferases. CBM, carbohydrate-binding module. CE, carbohydrate esterase. Distribution of six CAZyme classes as a proportion of the total number of predicted CAZyme, and COG functional classes as a proportion of the total number of predicted genes. * indicates unannotated category. **Fig S5.** Synteny and average nucleotide identity (ANI) comparisons between the *Basfia* metagenome-assembled genomes (MAGs) obtained from the giant panda gut microbiomes and the genome from the only described species of the *Basfia* genus from ruminants, *Basfia succiniciproducens*. Five circular *Basfia* genomes from giant panda microbiomes exhibited better synteny and higher ANI than compared to the *B. succiniciproducens* genome, suggesting the presence of a potentially new species of *Basfia*. **Fig S6.** Contig and gene characteristics of the pandaGUT database. (A) Distribution of assembled contig lengths. (B) Distribution of gene lengths from data assembled with short- and long-read sequencing data. The dark and light colours indicate complete and incomplete genes, respectively. (C) The percentages of genes annotated and reads mapped to the pandaGUT database obtained in this study exhibit a strong correlation. The empty circles indicate samples contaminated with > 1% human genome sequences, >10% giant panda genome sequences, or >1% bamboo genome sequences. **Fig S7.** Gut microbiome diversity from the three wild giant panda populations. (A) Shannon index of microbial diversity. (B) Bray–Curtis distances between pairwise comparisons of communities. **Fig S8.** Differentially abundant KEGG pathways in the microbiomes from the three wild giant panda populations. (A) Linear discriminant analysis (LDA) effect size (LEfSe) analysis used to identify key KEGG pathways that differentiated samples (LDA scores > 2 and *p* <0.05) in the gut microbiota among the three panda genetic populations. The numbers on the right indicate the numbers of corresponding pathways in Fig. 2E. (B) Reconstruction of nucleotide metabolic pathways. **Fig S9.** Strain-level analysis of single-nucleotide polymorphisms (SNPs) within the cellulose-degrading genes of six significantly different *Clostridium* species. (A) SNP abundances and read mapping rates in cellulose-degrading genes like cellulase (*cel*), beta-glucosidase (*bglX*), and xylan 1,4-beta-xylosidase (*xynB*). SNPs are highlighted in red and the mapping regions are indicated in blue. Background colours indicate samples collected during the leaf-eating (green) and shoot-eating (orange) seasons. (B) SNP densities of the genes that encode enzymes involved in cellulose degradation and that indicate the strain heterogeneity of *Clostridium*. SNP density (0–0.05) was determined as the ratio of filtered SNP bases to the total mapping bases. Darker colours indicate greater densities. **Fig S10.** Enterotypes and giant panda gut microbiome resistomes. (A) Calinski–Harabasz (CH) index values indicating the optimal number of enterotype clusters. (B) The microbial composition profiles of each enterotype revealing one of three dominant bacterial genera, including *Clostridium*,*Pseudomonas,* and *Escherichia*. (C) Sankey diagram showing the ARG composition and relative abundances of metagenome-assembled genomes of each enterotype. (D) UPGMA phylogenetic tree revealing six clusters of resistome profiles. (E) Cramer’s V statistics indicating the correlations among enterotype, captivity status, geographic distribution, and diet with resistome cluster. (F) Schematic showing the genetic organization of novel integrative and conjugative elements (ICEs) identified from the genomes of *Veillonella parvula* and *Streptococcus* sp. 002300045. Lines connecting blocks with identical colours indicate aligned regions and reveal synteny or gene rearrangements. The *tet*(M) gene is indicated in red. Essential modules of the ICE machinery are indicated in orange, blue, and pink for mobilization, recombination, and regulation genes, respectively.

**Additional file 2: Table S1.** Samples and corresponding information regarding collection location, captivity status, and sequencing data collected in this study. n/a, not available. **Table S2.** Assembly and annotation information for 820 metagenome-assembled genomes. TGS, single-molecular sequencing data. NGS, Illumina sequencing data.** Table S3.** Genome characteristics for the 40 circular metagenome-assembled genomes. **Table S4.** Annotation information of the CAZyme genes of 40 circular metagenome-assembled genomes. **Table S5.** COG annotations for all nonredundant microbial genes.

**Additional file 3.** Circos plot for the 40 complete metagenome-assembled genomes.

## Data Availability

Raw sequencing data and metagenome-assembled genomes generated in this study have been deposited to the National Genomics Data Center, China. Source data are provided in a supplementary file.
