# Supplementary material for: PandaGUT provides new insights into bacterial diversity, function, and resistome landscapes with implications for conservation
Source: Microbiome. 2023 Oct 7;11:221. doi: 10.1186/s40168-023-01657-0 (PMC10559513; doi:10.1186/s40168-023-01657-0)
Supplement: Supplementary file 2 — Additional file 1: Fig S1. Pipeline for the construction of the unified pandaGUT reference catalogue of the giant panda gut microbiome. Approximately 7 Tbp of long- and short-read sequencing data from diverse samples spanning different genetic backgrounds, dietary regimes, ages, sexes, and geographic distributions were integrated to construct the catalogue. pandaGUT contains 502 nonredundant MAGs and 2.37 million unique genes. Fig S2. Overview of sample and metagenome data integrated in the pandaGUT catalogue. (A) Metagenome statistics for de novo samples generated in this study and six other publicly available studies. The dark colours indicate the total numbers of the samples, and the light colours indicate the numbers of the samples contaminated by either > 1% human genome sequences, > 10% giant panda genome sequences, or > 1% bamboo genome sequences in the corresponding study. (B) Metagenome statistics for the metagenomic sequencing data de novo generated in this study and data from six other publicly available studies. The dashed square indicates Nanopore and PacBio sequencing data statistics. Fig S3. Genome quality of species representatives. (A) The completeness and contamination of all bins before (red colour) and after (green colour) refining by merging, splitting, and decontaminating. (B) GC content and MAG size statistics. (C) Synteny analysis (left) and statistics of genomic features (right) between the isolated and sequenced genome (ISG) and metagenome-assembled genome (MAG) of Morganella morganii. (D) Coverage of Nanopore and Illumina sequencing reads that were mapped to the M. morganii genome in addition to GC content distribution. Fig S4. Annotation information for the Clusters of Orthologous Genes (COG) and CAZyme genes within 174 high-quality metagenome-assembled genomes. AA, auxiliary activities. PL, polysaccharide lyase. GH, glycoside hydrolases. GT, glycosyl transferases. CBM, carbohydrate-binding module. CE, carbohydrate esterase. Distribution [file 40168_2023_1657_MOESM1_ESM.docx]

**Supporting Information for**

**PandaGUT provides new insights into bacterial diversity, function and resistome landscapes with implications for conservation**

Guangping Huang^1^†, Wenyu Shi^2^†, Le Wang^1^, Qingyue Qu^1^, Zhenqiang Zuo^3^, Jinfeng Wang^3^, Fangqing Zhao^3,5^*, Fuwen Wei^1,4,5^*

^1^CAS Key Laboratory of Animal Ecology and Conservation Biology, Institute of Zoology, Chinese Academy of Sciences, Beijing 100101, China;

^2^Microbial Resource and Big Data Center, Institute of Microbiology, Chinese Academy of Sciences, Beijing 100101, China;

^3^Laboratory for Computational Genomics, Beijing Institutes of Life Science, Chinese Academy of Sciences, Beijing 100101, China;

^4^College of Forestry, Jiangxi Agricultural University, Nanchang 330045, China;

^5^University of Chinese Academy of Sciences, Beijing 100049, China

†These authors contributed equally to this work.

*Correspondence: weifw@ioz.ac.cn (F.W.), zhfq@biols.ac.cn (F.Z.).

# **Supplementary Figures and Figure Legends**

**
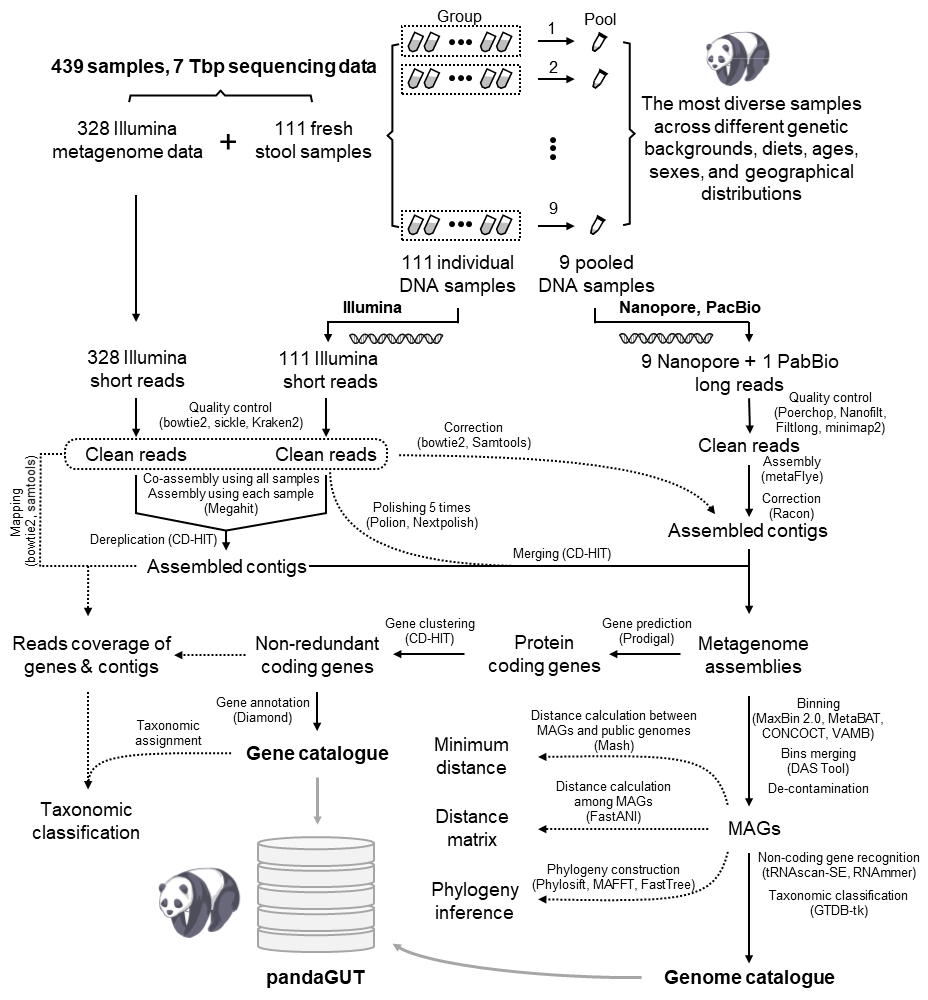
Fig S1. Pipeline for the construction of the unified pandaGUT reference catalogue of the giant panda gut microbiome.** Approximately 7 Tbp of long- and short-read sequencing data from diverse samples spanning different genetic backgrounds, dietary regimes, ages, sexes, and geographic distributions were integrated to construct the catalogue. pandaGUT contains 502 nonredundant MAGs and 2.37 million unique genes.


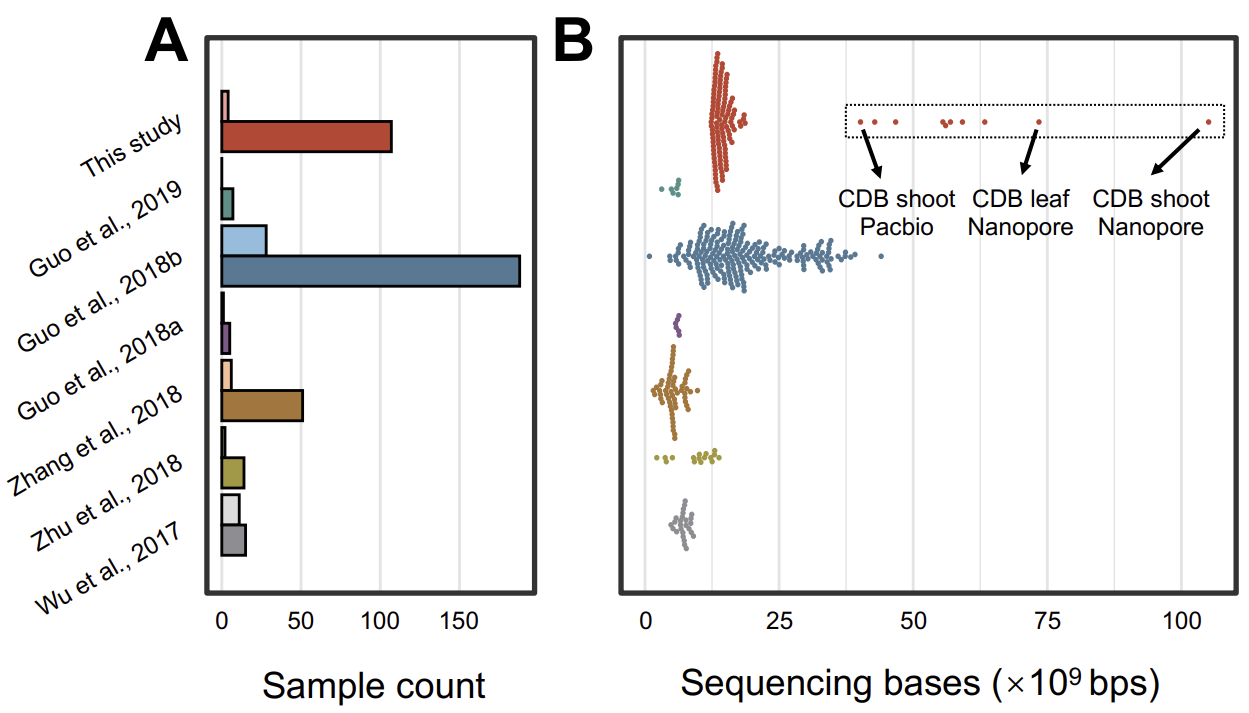


**Fig S2. Overview of sample and metagenome data integrated in the pandaGUT catalogue.** **(A)** Metagenome statistics for *de novo* samples generated in this study and six other publicly available studies. The dark colours indicate the total numbers of the samples, and the light colours indicate the numbers of the samples contaminated by either > 1% human genome sequences, > 10% giant panda genome sequences, or > 1% bamboo genome sequences in the corresponding study. **(B)** Metagenome statistics for the metagenomic sequencing data *de novo* generated in this study and data from six other publicly available studies. The dashed square indicates Nanopore and PacBio sequencing data statistics.


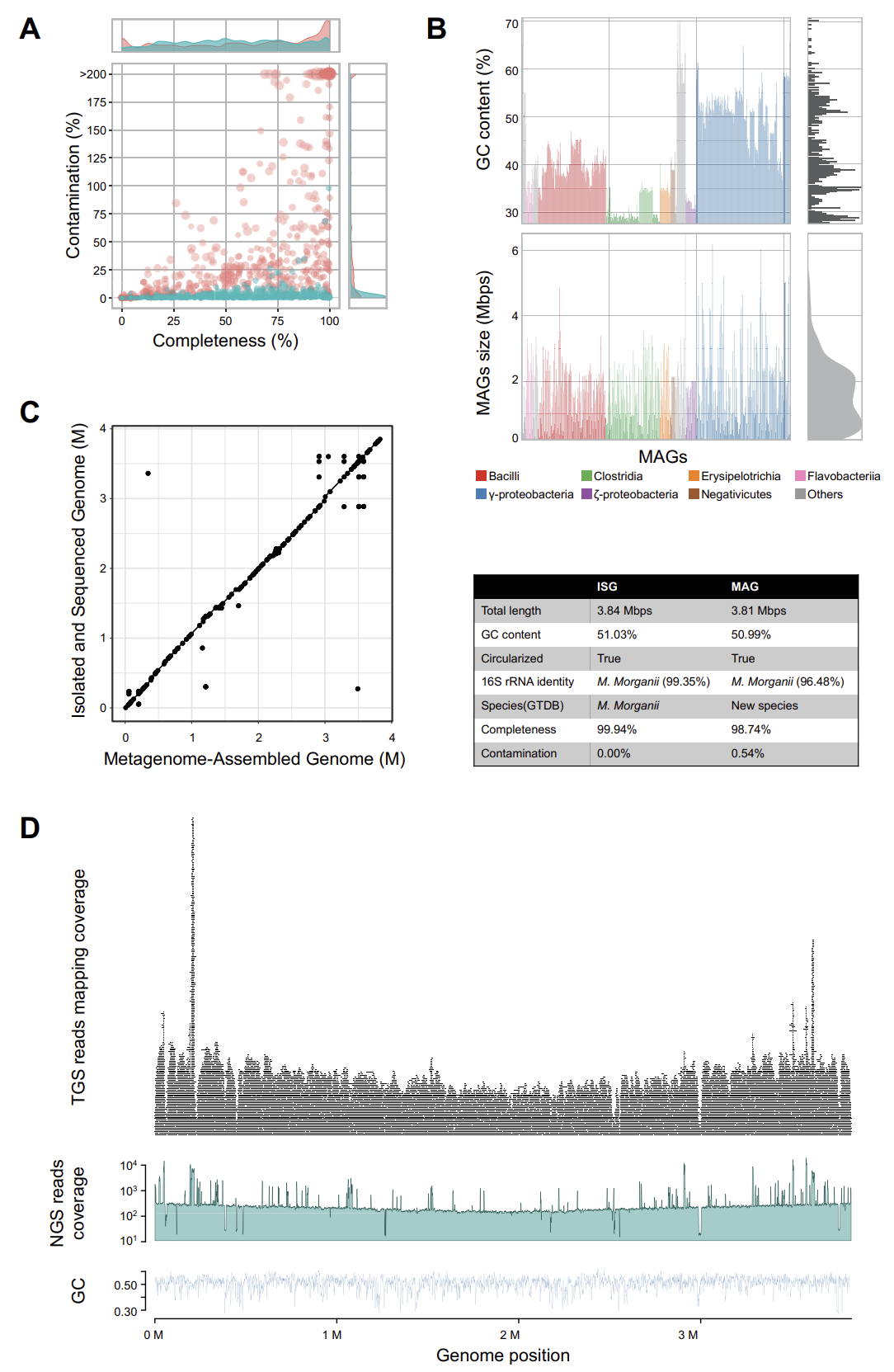


**Fig S3. Genome quality of species representatives.** **(A)** The completeness and contamination of all bins before (red colour) and after (green colour) refining by merging, splitting, and decontaminating. **(B)** GC content and MAG size statistics. **(C)** Synteny analysis (left) and statistics of genomic features (right) between the isolated and sequenced genome (ISG) and metagenome-assembled genome (MAG) of *Morganella morganii*. **(D)** Coverage of Nanopore and Illumina sequencing reads that were mapped to the *M. morganii* genome in addition to GC content distribution.


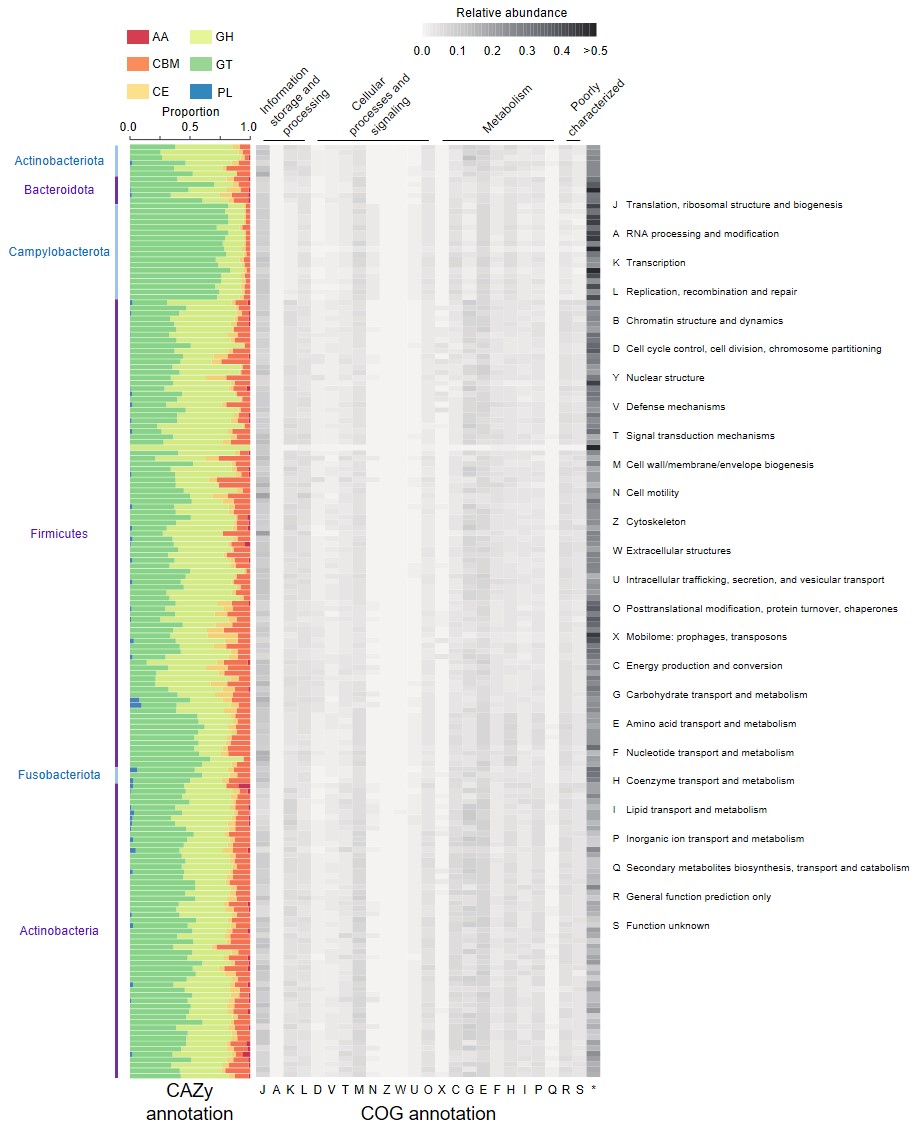
 **Fig S4.** **Annotation information for the Clusters of Orthologous Genes (COG) and CAZyme genes within 174 high-quality metagenome-assembled genomes.** AA, auxiliary activities. PL, polysaccharide lyase. GH, glycoside hydrolases. GT, glycosyl transferases. CBM, carbohydrate-binding module. CE, carbohydrate esterase. Distribution of six CAZyme classes as a proportion of the total number of predicted CAZyme, and COG functional classes as a proportion of the total number of predicted genes. * indicates unannotated category.


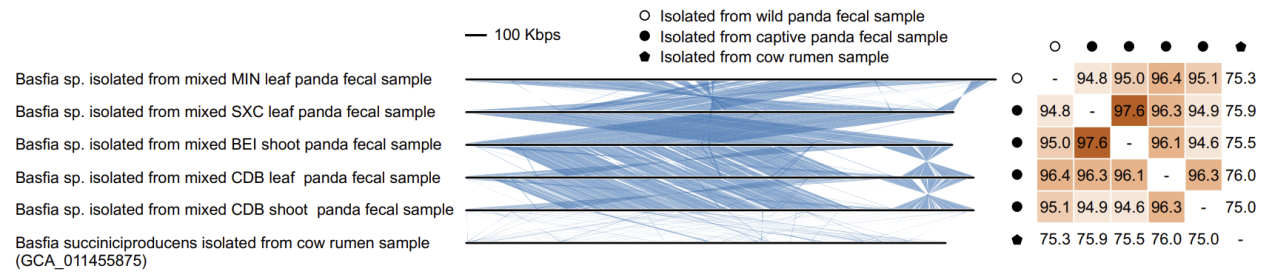


**Fig S5. Synteny and average nucleotide identity (ANI) comparisons between the *Basfia* metagenome-assembled genomes (MAGs) obtained from the giant panda gut microbiomes and the genome from the only described species of the *Basfia* genus from ruminants, *Basfia succiniciproducens*.** Five circular *Basfia* genomes from giant panda microbiomes exhibited better synteny and higher ANI than compared to the *B. succiniciproducens* genome, suggesting the presence of a potentially new species of *Basfia*.


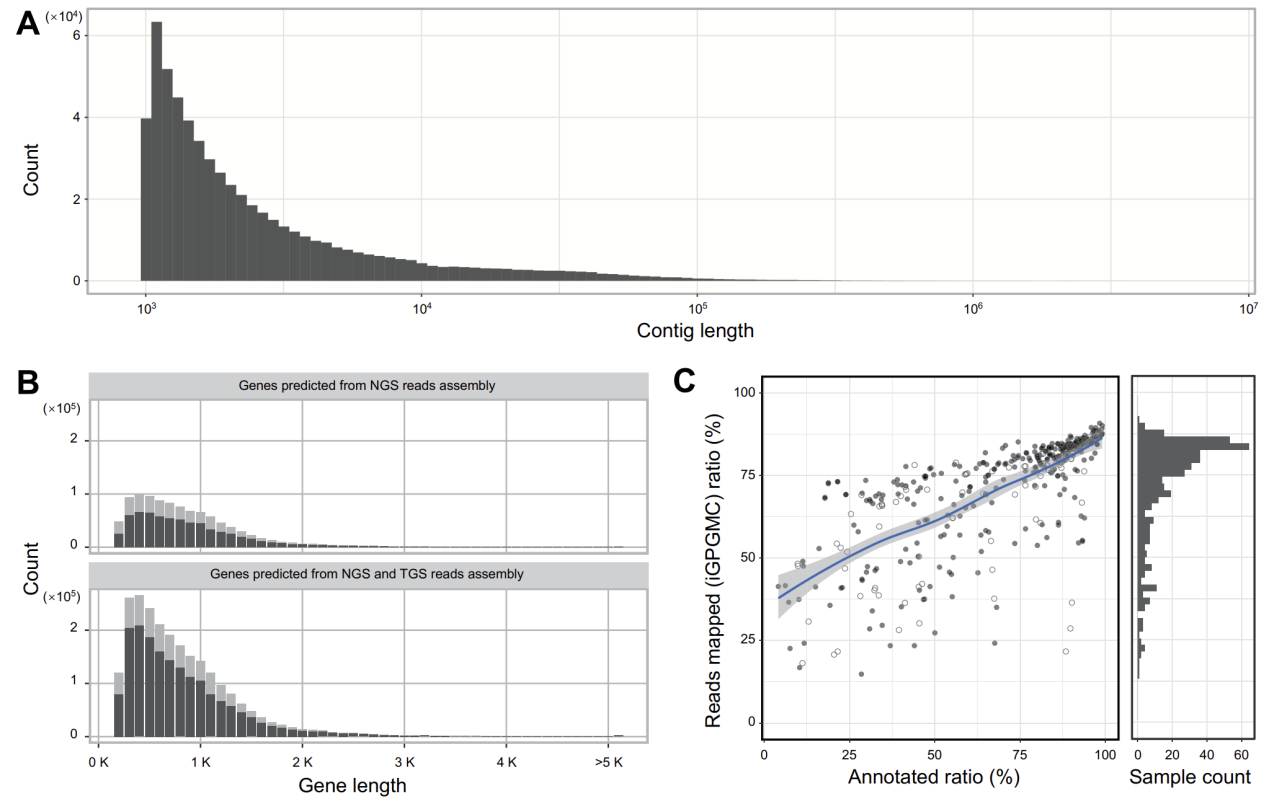


**Fig S6. Contig and gene characteristics of the pandaGUT database.** **(A)** Distribution of assembled contig lengths. **(B)** Distribution of gene lengths from data assembled with short- and long-read sequencing data. The dark and light colours indicate complete and incomplete genes, respectively. **(C)** The percentages of genes annotated and reads mapped to the pandaGUT database obtained in this study exhibit a strong correlation. The empty circles indicate samples contaminated with > 1% human genome sequences, >10% giant panda genome sequences, or >1% bamboo genome sequences.


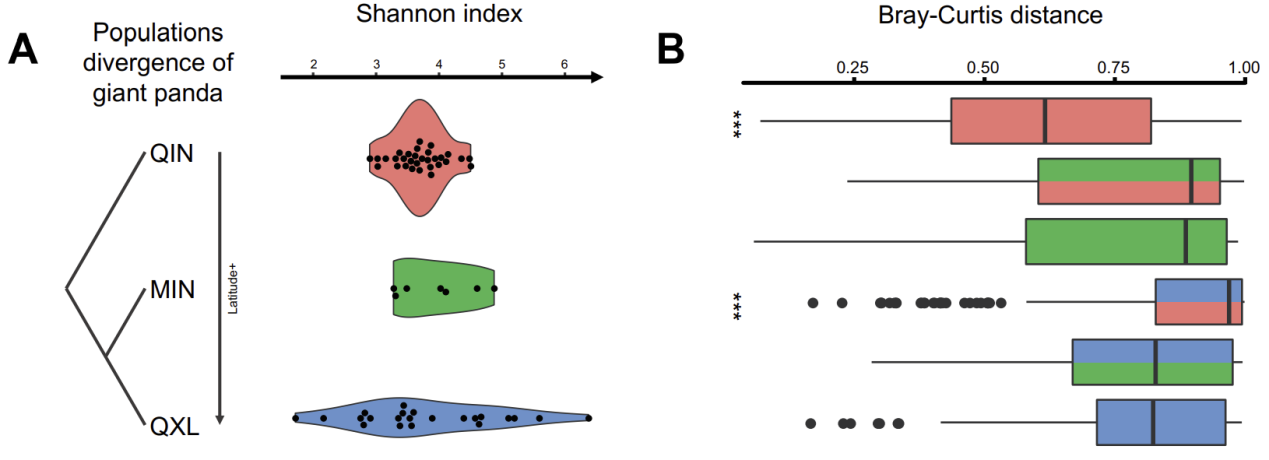


**Fig S7. Gut microbiome diversity from the three wild giant panda populations.** **(A)** Shannon index of microbial diversity. **(B)** Bray–Curtis distances between pairwise comparisons of communities.


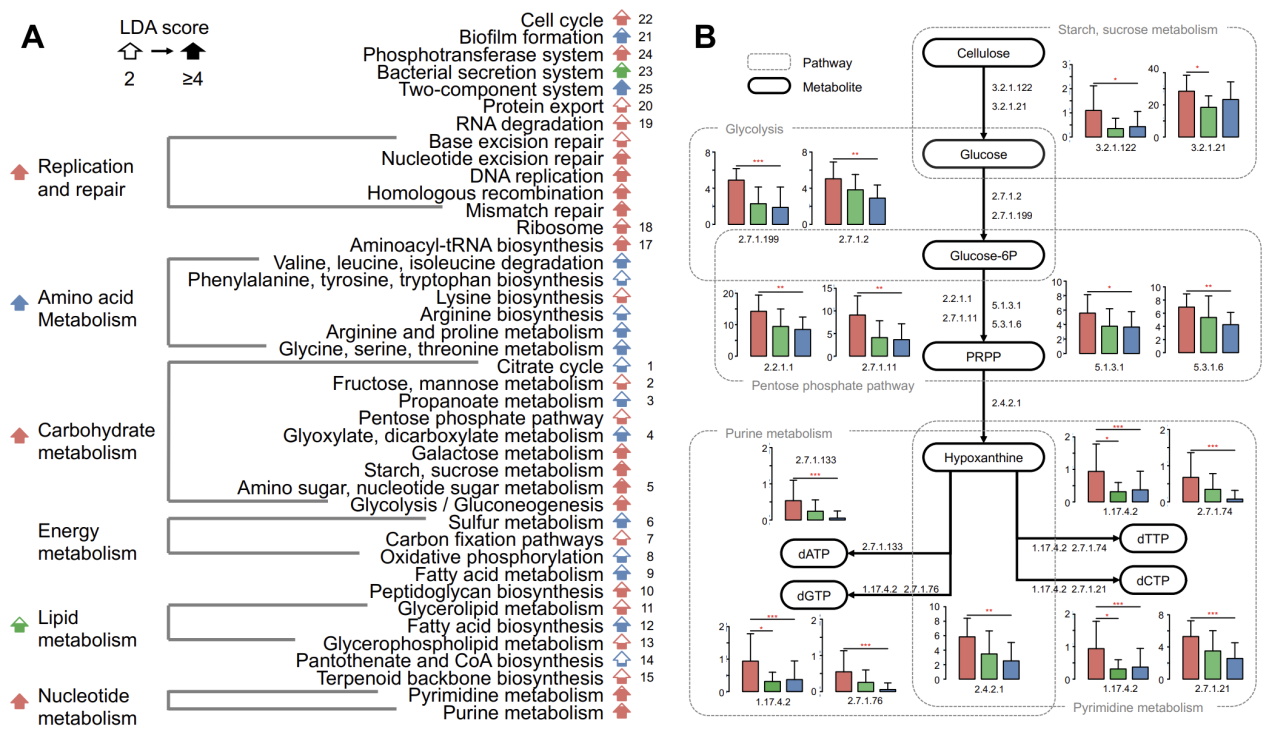


**Fig S8. Differentially abundant KEGG pathways in the microbiomes from the three wild giant panda populations. (A)** Linear discriminant analysis (LDA) effect size (LEfSe) analysis used to identify key KEGG pathways that differentiated samples (LDA scores > 2 and *p* <0.05) in the gut microbiota among the three panda genetic populations. The numbers on the right indicate the numbers of corresponding pathways in Fig. 2E. **(B)** Reconstruction of nucleotide metabolic pathways.


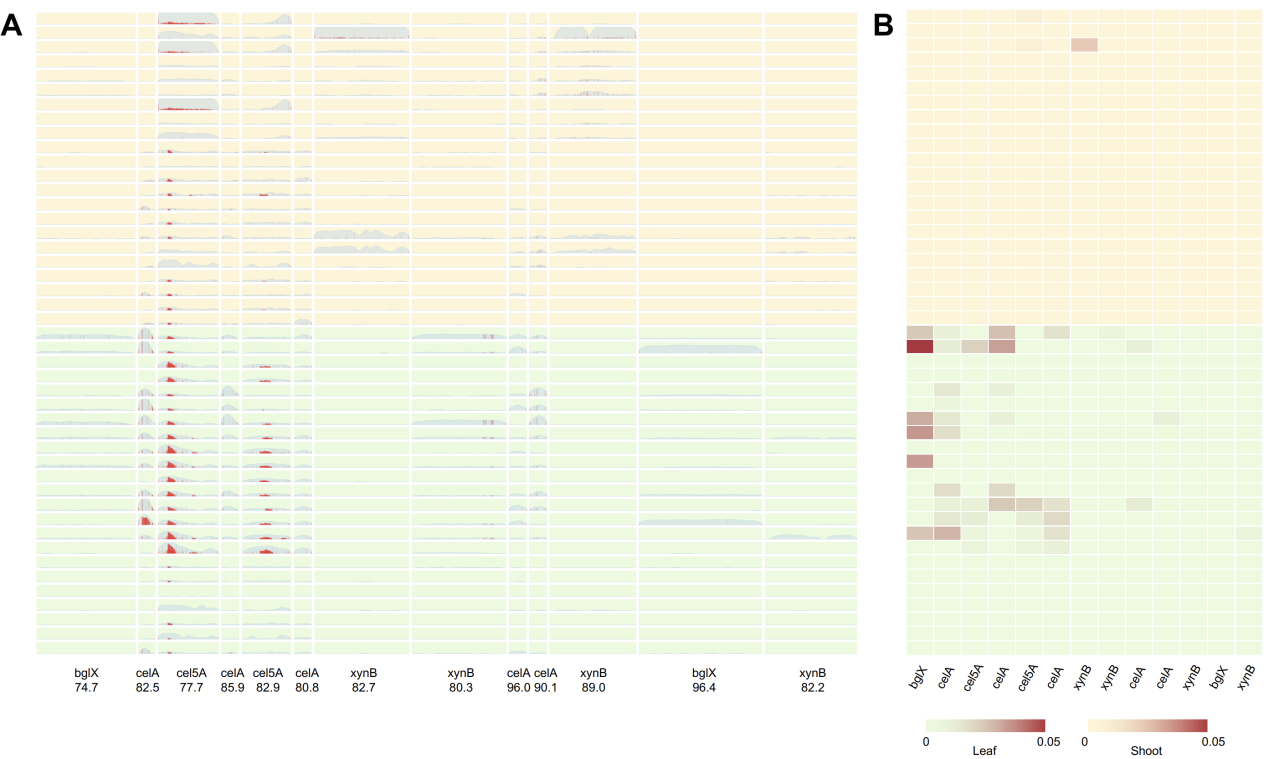


**Fig S9. Strain-level analysis of single-nucleotide polymorphisms (SNPs) within the cellulose-degrading genes of six significantly different *Clostridium* species.** **(A)** SNP abundances and read mapping rates in cellulose-degrading genes like cellulase (*cel*), beta-glucosidase (*bglX*), and xylan 1,4-beta-xylosidase (*xynB*). SNPs are highlighted in red and the mapping regions are indicated in blue. Background colours indicate samples collected during the leaf-eating (green) and shoot-eating (orange) seasons. **(B)** SNP densities of the genes that encode enzymes involved in cellulose degradation and that indicate the strain heterogeneity of *Clostridium*. SNP density (0–0.05) was determined as the ratio of filtered SNP bases to the total mapping bases. Darker colours indicate greater densities.


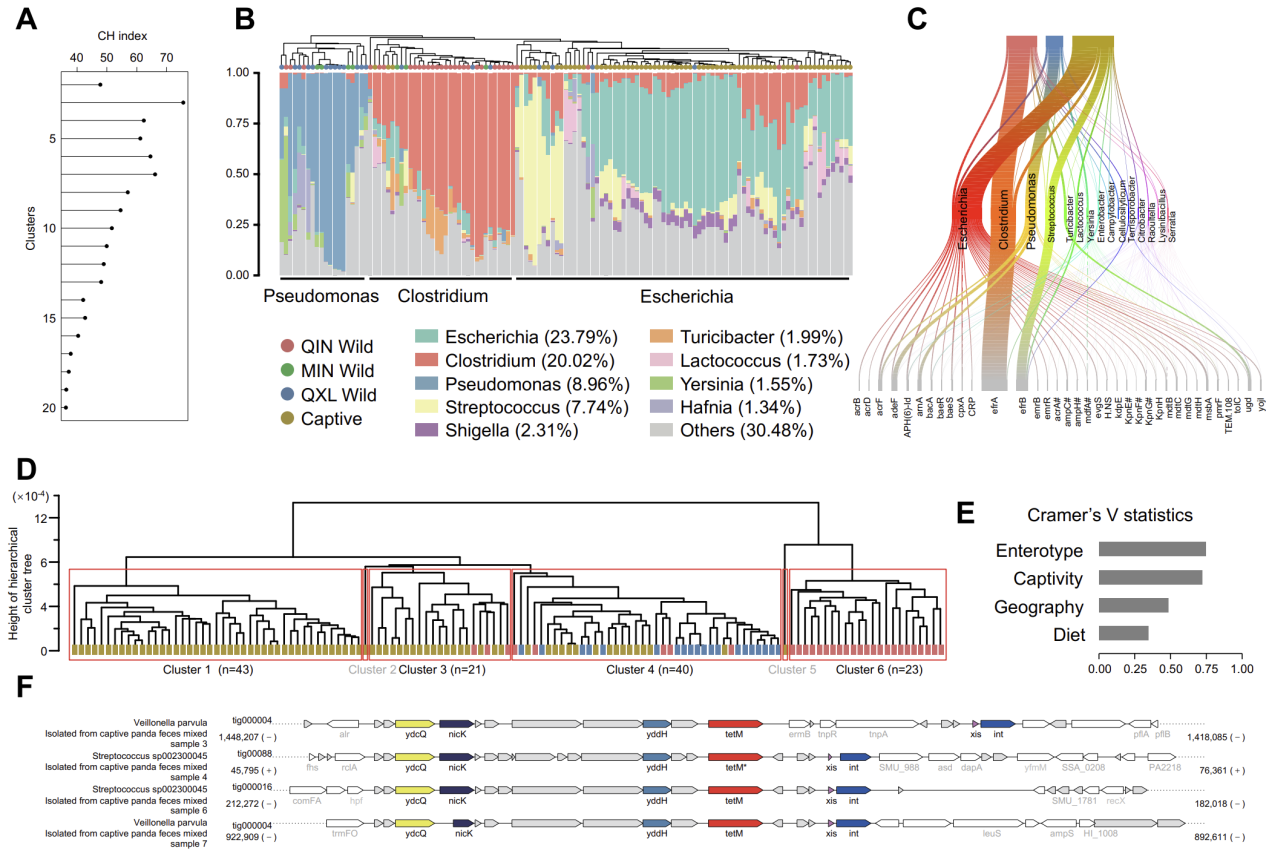


**Fig S10. Enterotypes and giant panda gut microbiome resistomes.** **(A)** Calinski–Harabasz (CH) index values indicating the optimal number of enterotype clusters. **(B)** The microbial composition profiles of each enterotype revealing one of three dominant bacterial genera, including *Clostridium*, *Pseudomonas,* and *Escherichia*. **(C)** Sankey diagram showing the ARG composition and relative abundances of metagenome-assembled genomes of each enterotype. **(D)** UPGMA phylogenetic tree revealing six clusters of resistome profiles. **(E)** Cramer’s V statistics indicating the correlations among enterotype, captivity status, geographic distribution, and diet with resistome cluster. **(F)** Schematic showing the genetic organization of novel integrative and conjugative elements (ICEs) identified from the genomes of *Veillonella parvula* and *Streptococcus* sp. 002300045. Lines connecting blocks with identical colours indicate aligned regions and reveal synteny or gene rearrangements. The *tet*(M) gene is indicated in red. Essential modules of the ICE machinery are indicated in orange, blue, and pink for mobilization, recombination, and regulation genes, respectively.

# Supplementary Tables

Table S1. Samples and corresponding information regarding collection location, captivity status, and sequencing data collected in this study. n/a, not available.

Table S2. Assembly and annotation information for 820 metagenome-assembled genomes. TGS, single-molecular sequencing data. NGS, Illumina sequencing data.

Table S3. Genome characteristics for the 40 circular metagenome-assembled genomes.

Table S4. Annotation information of the CAZyme genes of 40 circular metagenome-assembled genomes.

Table S5. COG annotations for all nonredundant microbial genes.

# Supplementary Data 1

Circos plot for the 40 complete metagenome-assembled genomes.
