# Supplementary material for: PandaGUT provides new insights into bacterial diversity, function, and resistome landscapes with implications for conservation
Source: Microbiome. 2023 Oct 7;11:221. doi: 10.1186/s40168-023-01657-0 (PMC10559513; doi:10.1186/s40168-023-01657-0)
Supplement: Supplementary file 4 — Additional file 3. Circos plot for the 40 complete metagenome-assembled genomes. [file 40168_2023_1657_MOESM3_ESM.pdf]

Supplementary data file

Circos plot of 40 complete metagenome-assembled genomes. From the inner circle to the outer circle, GC skew, GC content and gene profiles were indicated successively. GC skew, blue for positive and purple for negative. GC content, black for higher and gray for lower than average. In the outer layers, the triangles represent coding genes. Orange for genetic information storage and processing genes, green for cellular processing and signalling genes, brown for metabolic genes, grey for poorly characterized genes. The rectangles represent rRNA and tRNA.

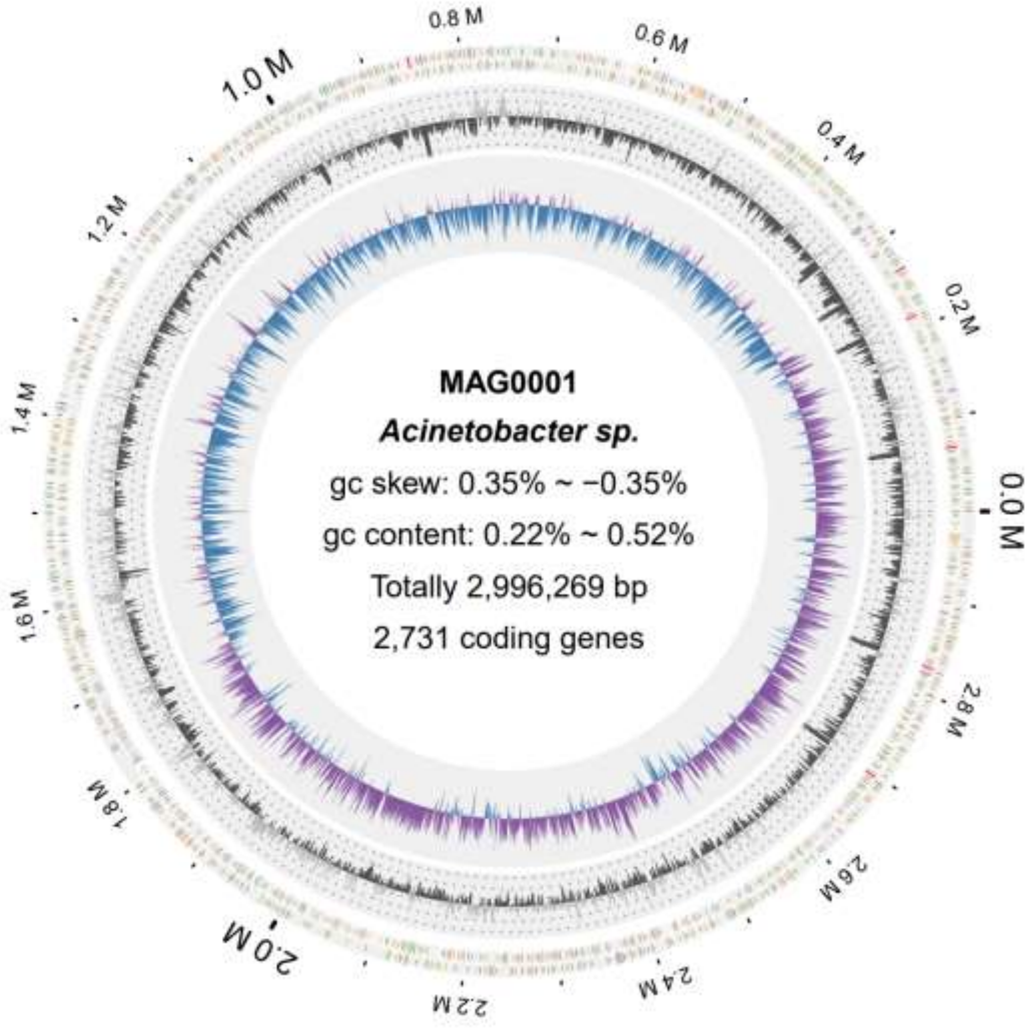

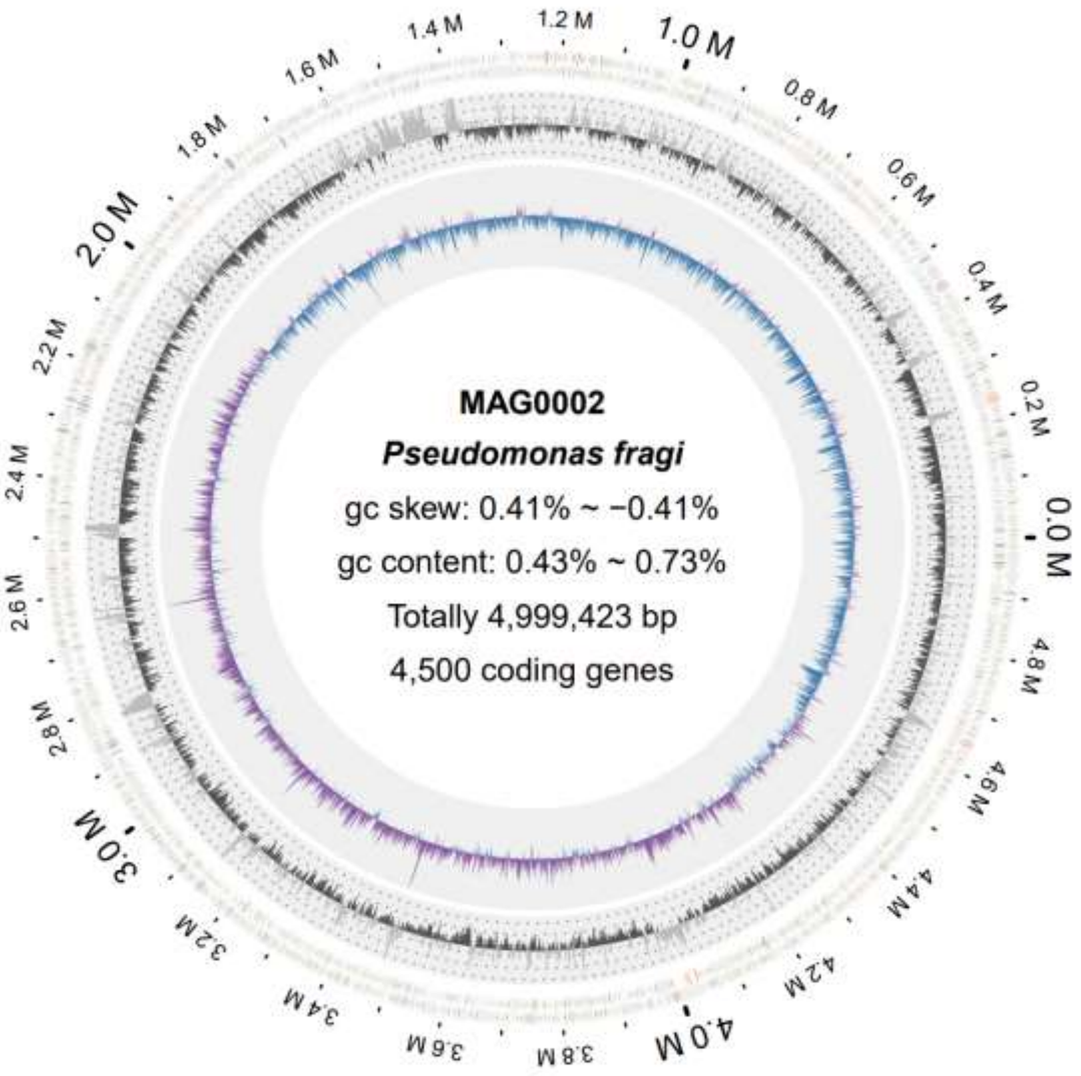
MAG0002
Pseudomonas fragi
gc skew: 0.41% ~ −0.41%
gc content: 0.43% ~ 0.73%
Totally 4,999,423 bp
4,500 coding genes
0.0 M
0.2 M
0.4 M
0.6 M
0.8 M
1.0 M
1.2 M
1.4 M
1.6 M
1.8 M
2.0 M
2.2 M
2.4 M
2.6 M
2.8 M
3.0 M
3.2 M
3.4 M
3.6 M
3.8 M
4.0 M
4.2 M
4.4 M
4.6 M
4.8 M

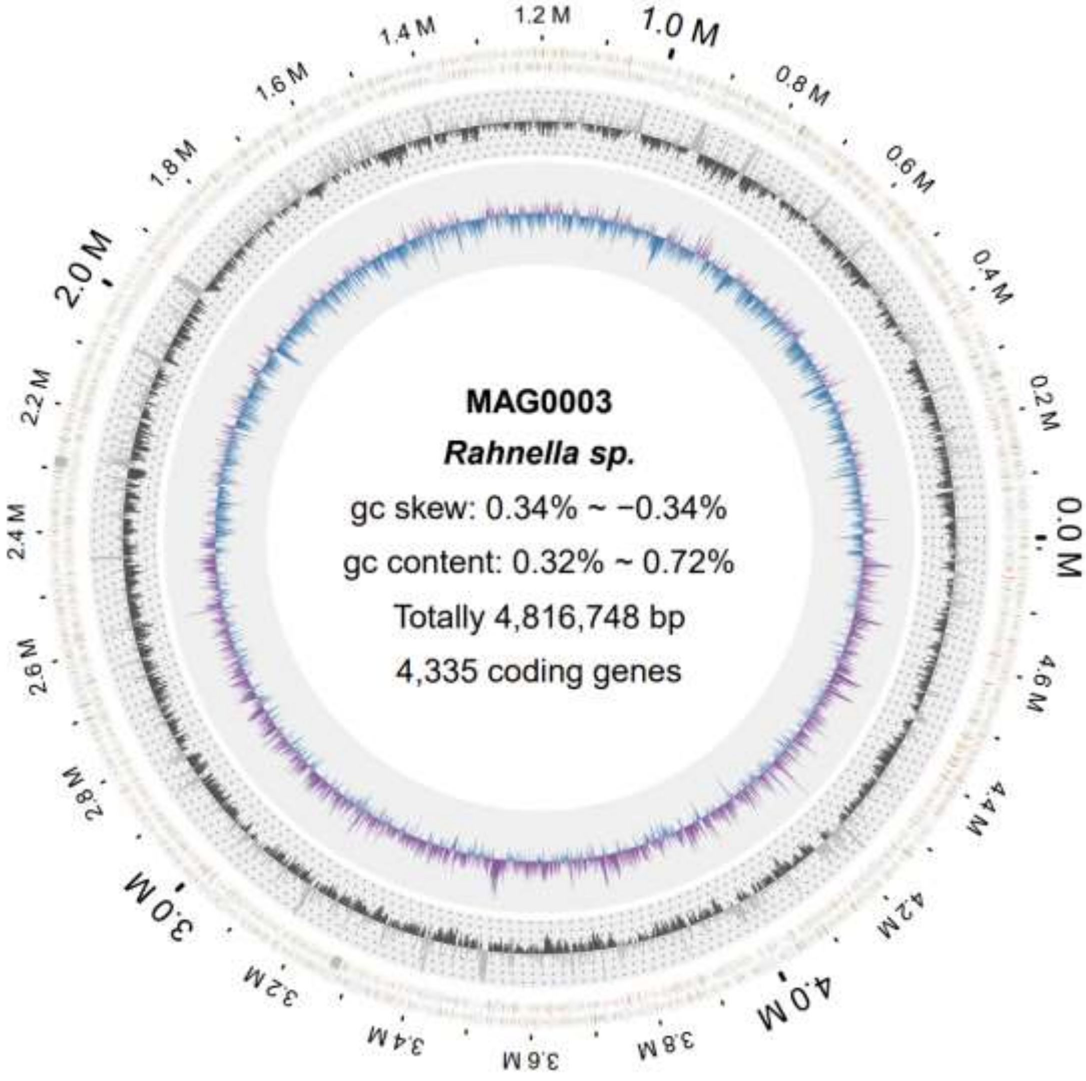
MAG0003
Rahnella sp.
gc skew: 0.34% ~ −0.34%
gc content: 0.32% ~ 0.72%
Totally 4,816,748 bp
4,335 coding genes
0.0 M
0.2 M
0.4 M
0.6 M
0.8 M
1.0 M
1.2 M
1.4 M
1.6 M
1.8 M
2.0 M
2.2 M
2.4 M
2.6 M
2.8 M
3.0 M
3.2 M
3.4 M
3.6 M
3.8 M
4.0 M
4.2 M
4.4 M
4.6 M

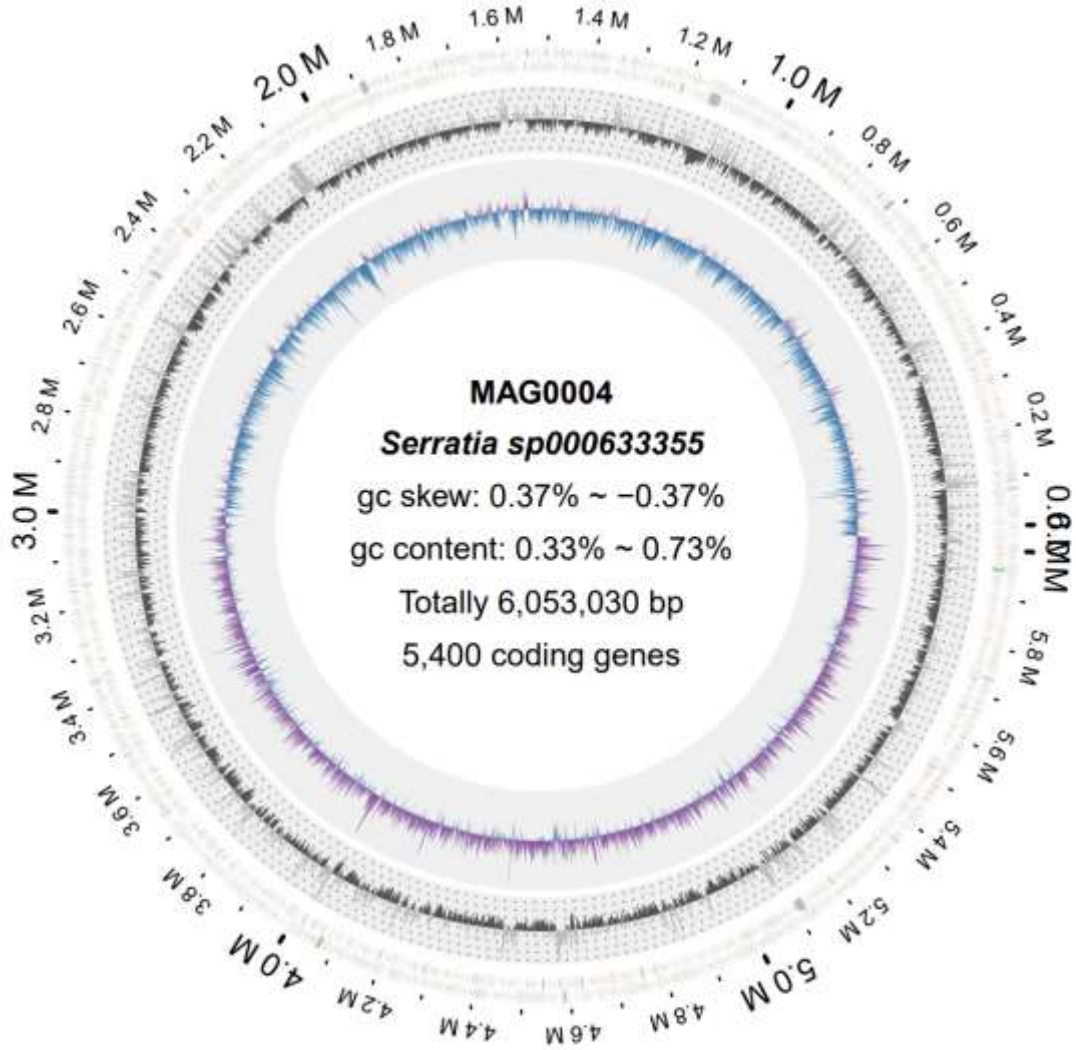
MAG0004
Serratia sp000633355
gc skew: 0.37% ~ −0.37%
gc content: 0.33% ~ 0.73%
Totally 6,053,030 bp
5,400 coding genes
0.2 M
0.4 M
0.6 M
0.8 M
1.0 M
1.2 M
1.4 M
1.6 M
1.8 M
2.0 M
2.2 M
2.4 M
2.6 M
2.8 M
3.0 M
3.2 M
3.4 M
3.6 M
3.8 M
4.0 M
4.2 M
4.4 M
4.6 M
4.8 M
5.0 M
5.2 M
5.4 M
5.6 M
5.8 M

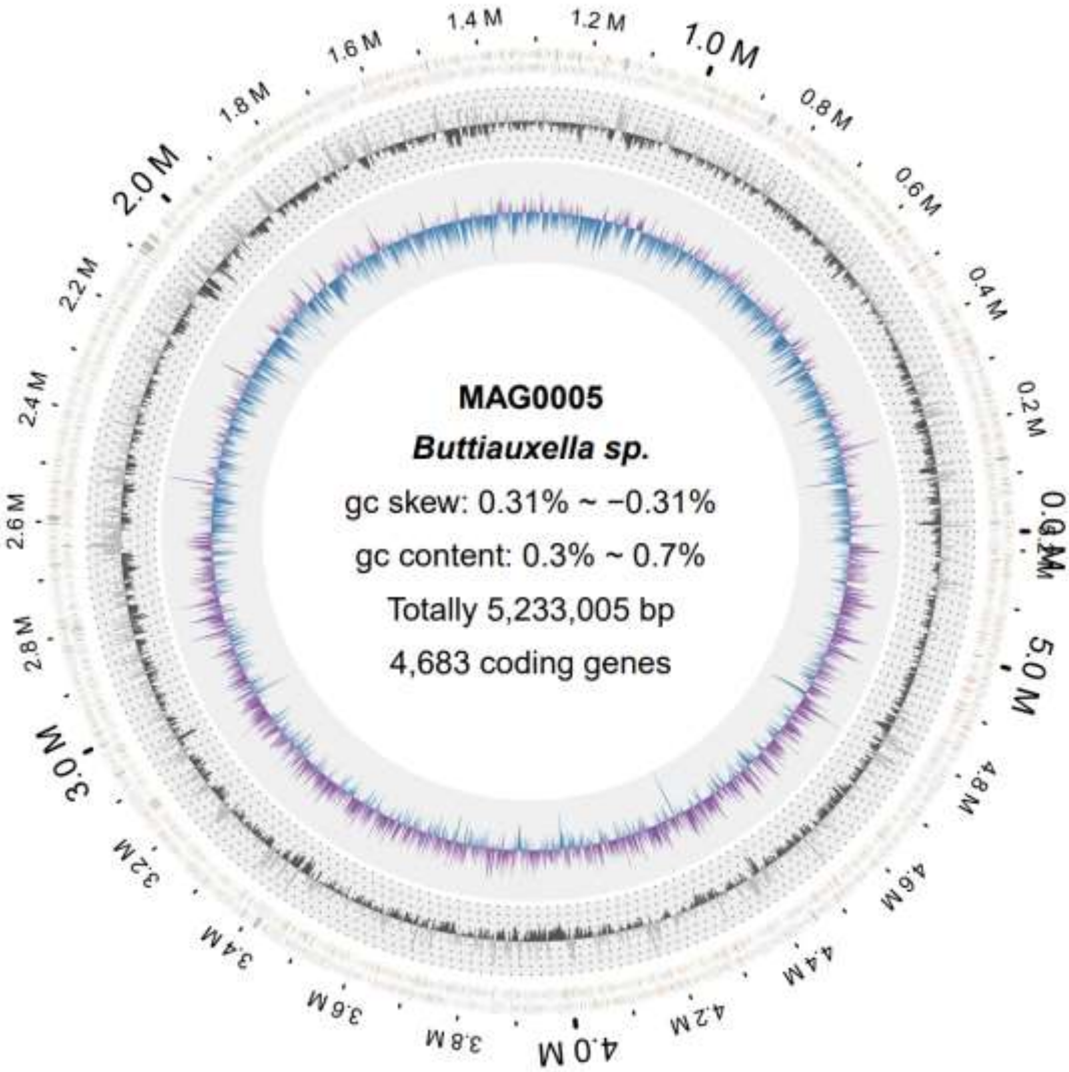

MAG0005
Buttiauxella sp.
gc skew: 0.31% ~ −0.31%
gc content: 0.3% ~ 0.7%
Totally 5,233,005 bp
4,683 coding genes
0.0 M
0.2 M
0.4 M
0.6 M
0.8 M
1.0 M
1.2 M
1.4 M
1.6 M
1.8 M
2.0 M
2.2 M
2.4 M
2.6 M
2.8 M
3.0 M
3.2 M
3.4 M
3.6 M
3.8 M
4.0 M
4.2 M
4.4 M
4.6 M
4.8 M
5.0 M

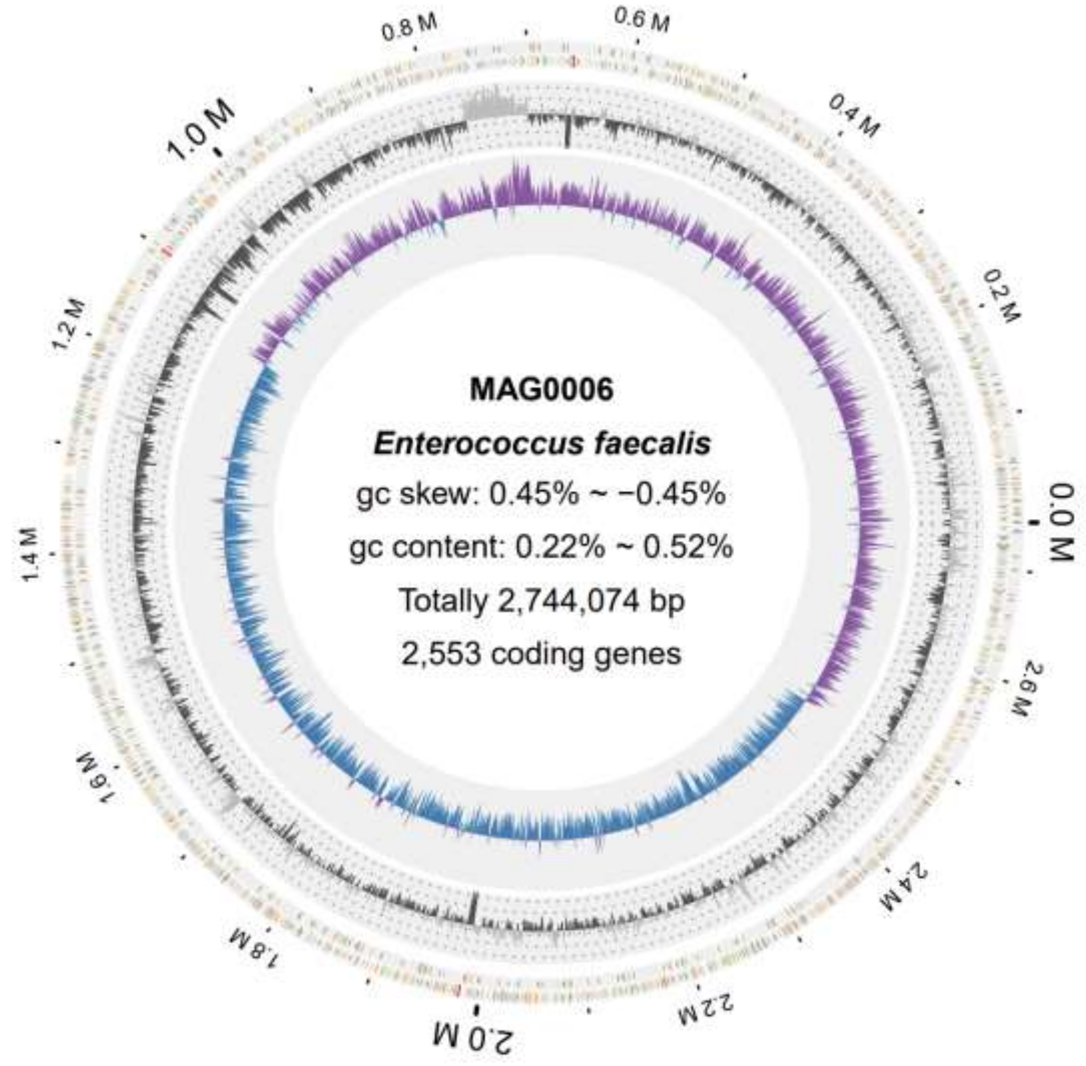

MAG0006
Enterococcus faecalis
gc skew: 0.45% ~ −0.45%
gc content: 0.22% ~ 0.52%
Totally 2,744,074 bp
2,553 coding genes
0.0 M
0.2 M
0.4 M
0.6 M
0.8 M
1.0 M
1.2 M
1.4 M
1.6 M
1.8 M
2.0 M
2.2 M
2.4 M
2.6 M

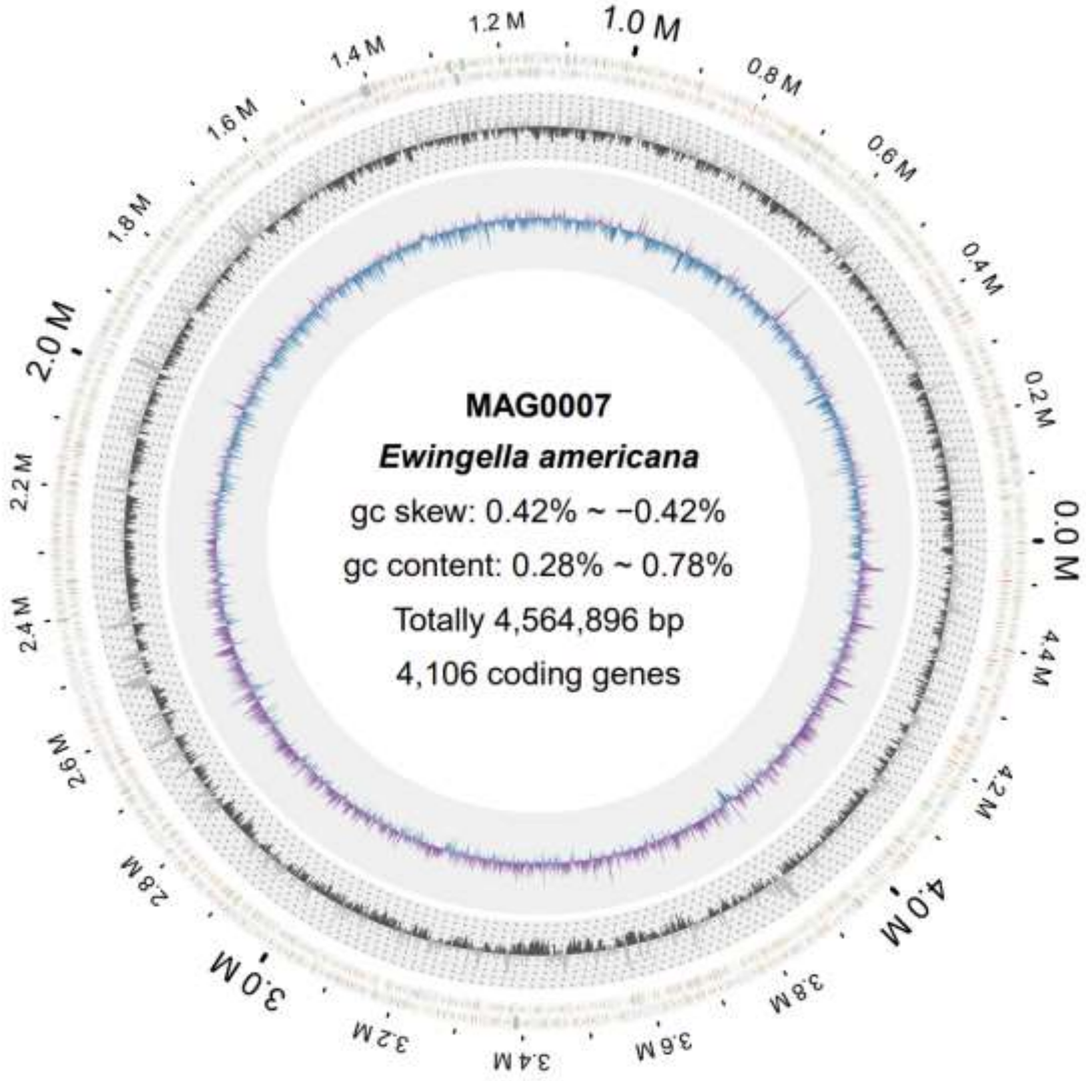
MAG0007
Ewingella americana
gc skew: 0.42% ~ −0.42%
gc content: 0.28% ~ 0.78%
Totally 4,564,896 bp
4,106 coding genes
0.0 M
0.2 M
0.4 M
0.6 M
0.8 M
1.0 M
1.2 M
1.4 M
1.6 M
1.8 M
2.0 M
2.2 M
2.4 M
2.6 M
2.8 M
3.0 M
3.2 M
3.4 M
3.6 M
3.8 M
4.0 M
4.2 M
4.4 M

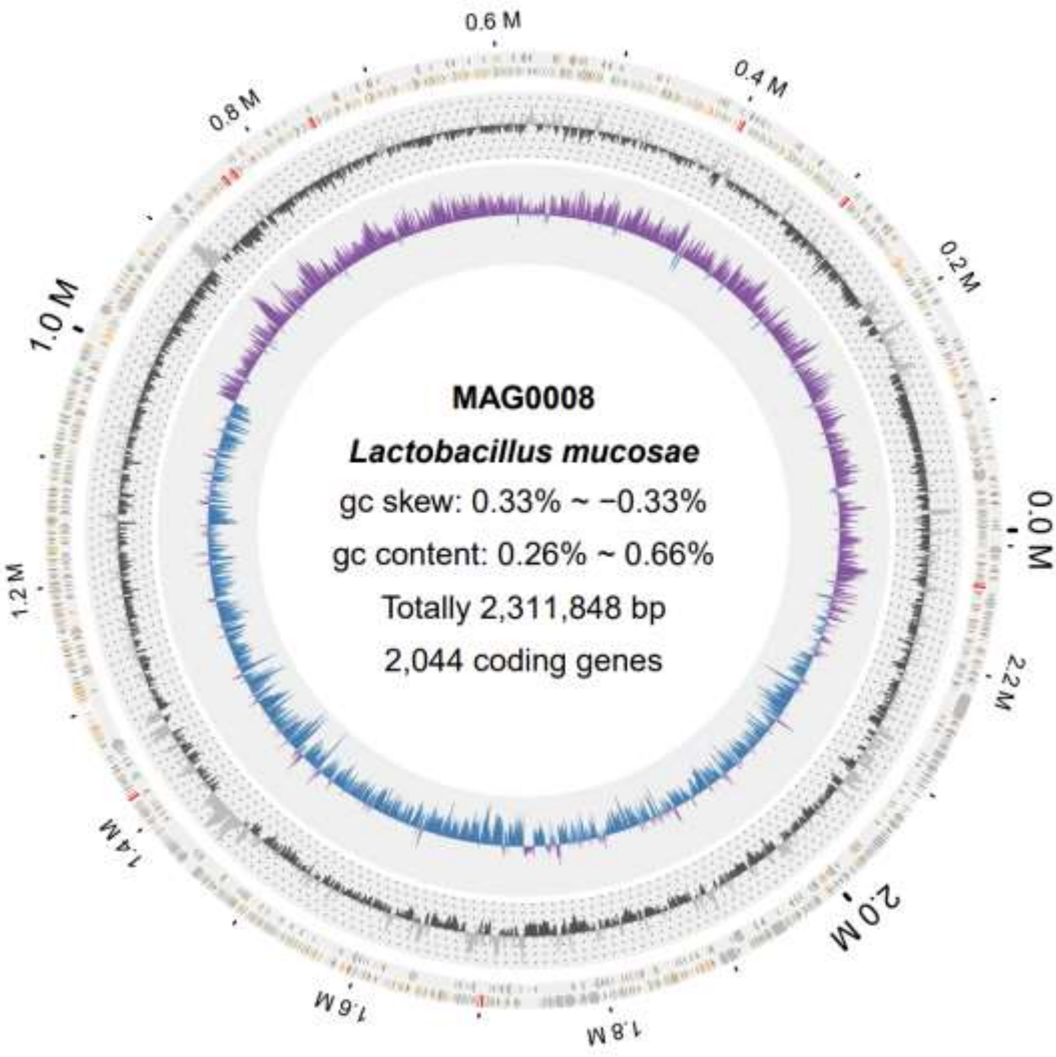

0.6 M
0.4 M
0.8 M
0.2 M
1.0 M
MAG0008
Lactobacillus mucosae
gc skew: 0.33% ~ −0.33%
gc content: 0.26% ~ 0.66%
Totally 2,311,848 bp
2,044 coding genes
0.0 M
1.2 M
2.2 M
1.4 M
2.0 M
1.6 M
1.8 M

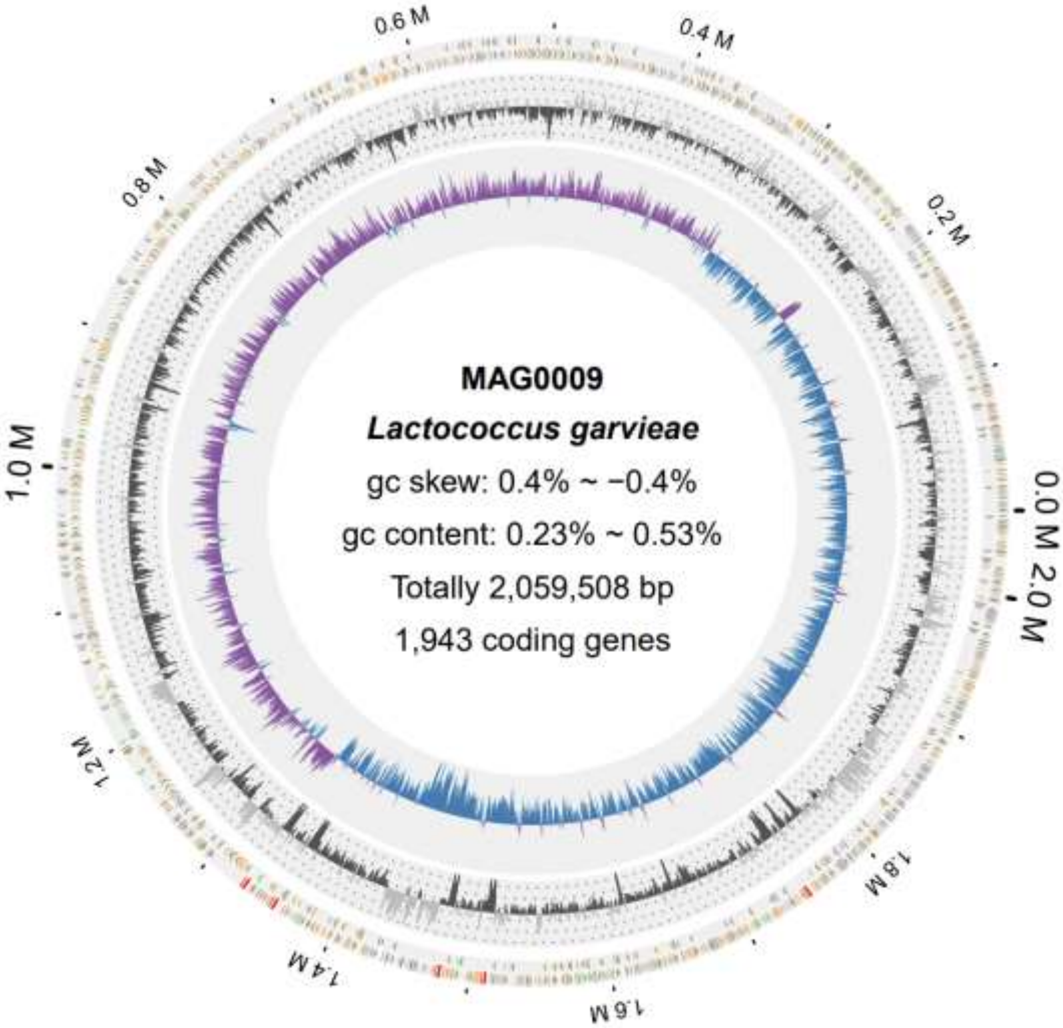
MAG0009
Lactococcus garvieae
gc skew: 0.4% ~ −0.4%
gc content: 0.23% ~ 0.53%
Totally 2,059,508 bp
1,943 coding genes
0.0 M
0.2 M
0.4 M
0.6 M
0.8 M
1.0 M
1.2 M
1.4 M
1.6 M
1.8 M
2.0 M

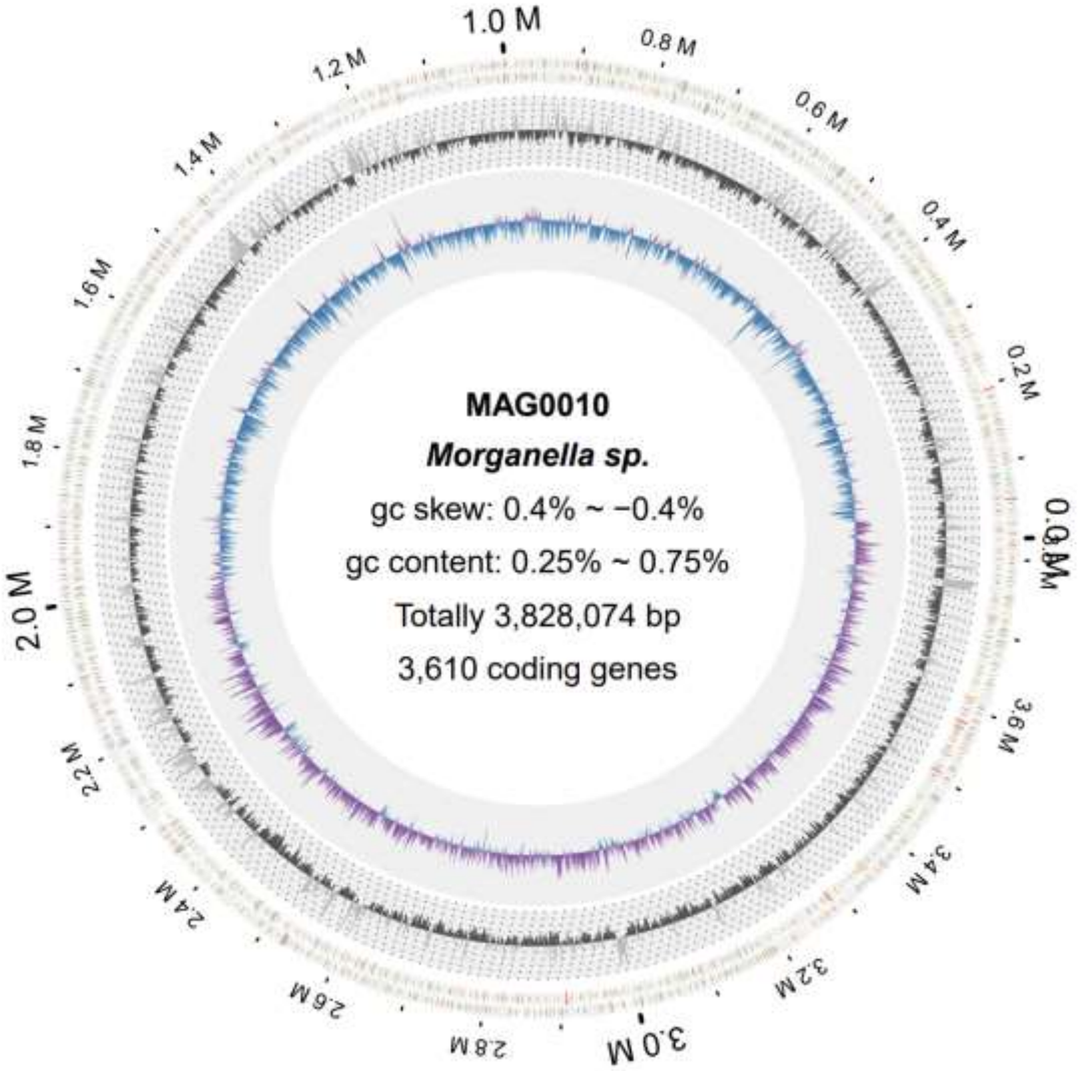

MAG0010
Morganella sp.
gc skew: 0.4% ~ −0.4%
gc content: 0.25% ~ 0.75%
Totally 3,828,074 bp
3,610 coding genes
1.0 M
0.8 M
0.6 M
0.4 M
0.2 M
1.2 M
1.4 M
1.6 M
1.8 M
2.0 M
2.2 M
2.4 M
2.6 M
2.8 M
3.0 M
3.2 M
3.4 M
3.6 M

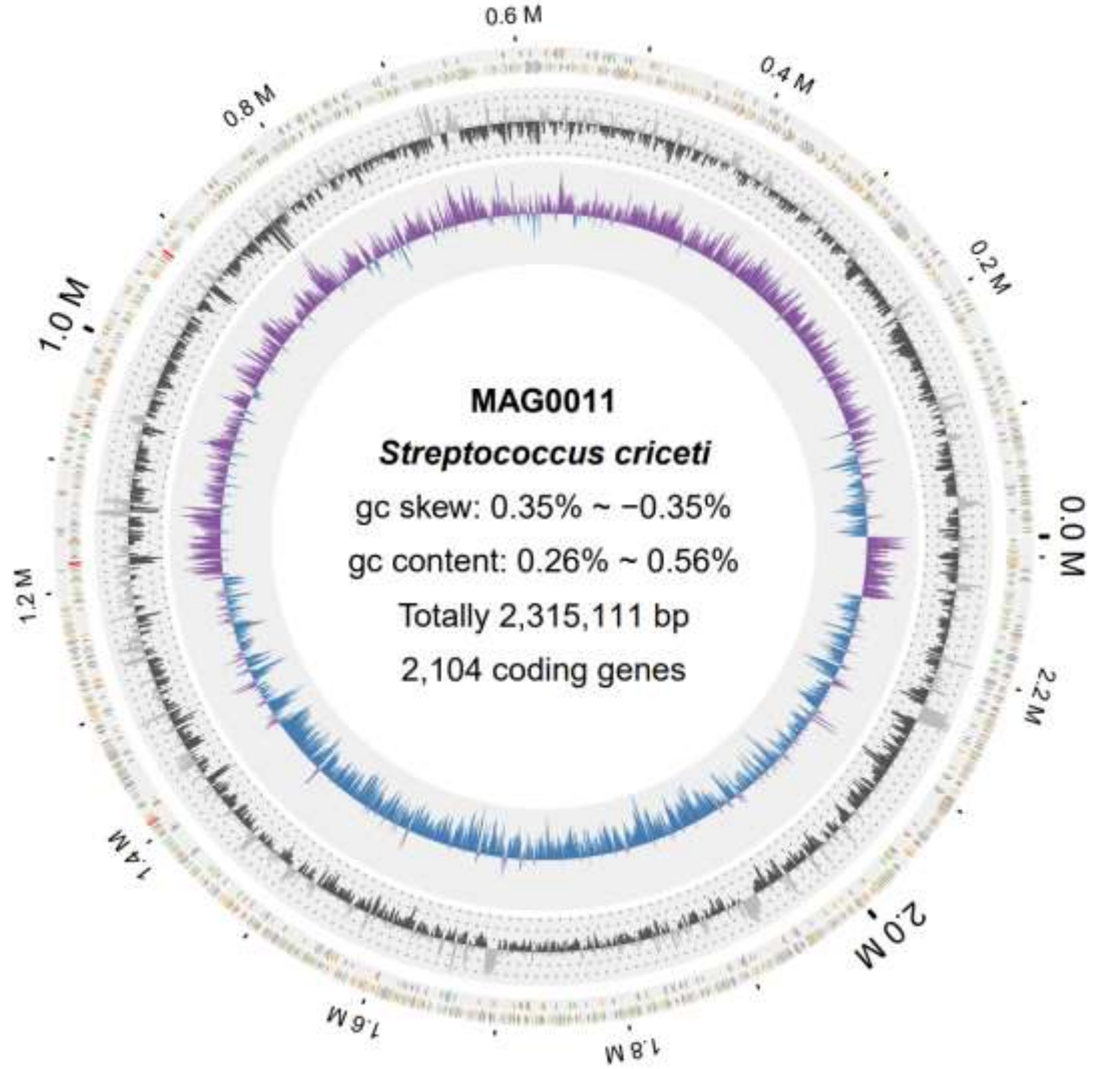
0.6 M
0.4 M
0.8 M
0.2 M
1.0 M
MAG0011
Streptococcus criceti
gc skew: 0.35% ~ −0.35%
gc content: 0.26% ~ 0.56%
Totally 2,315,111 bp
2,104 coding genes
0.0 M
1.2 M
2.2 M
1.4 M
2.0 M
1.6 M
1.8 M

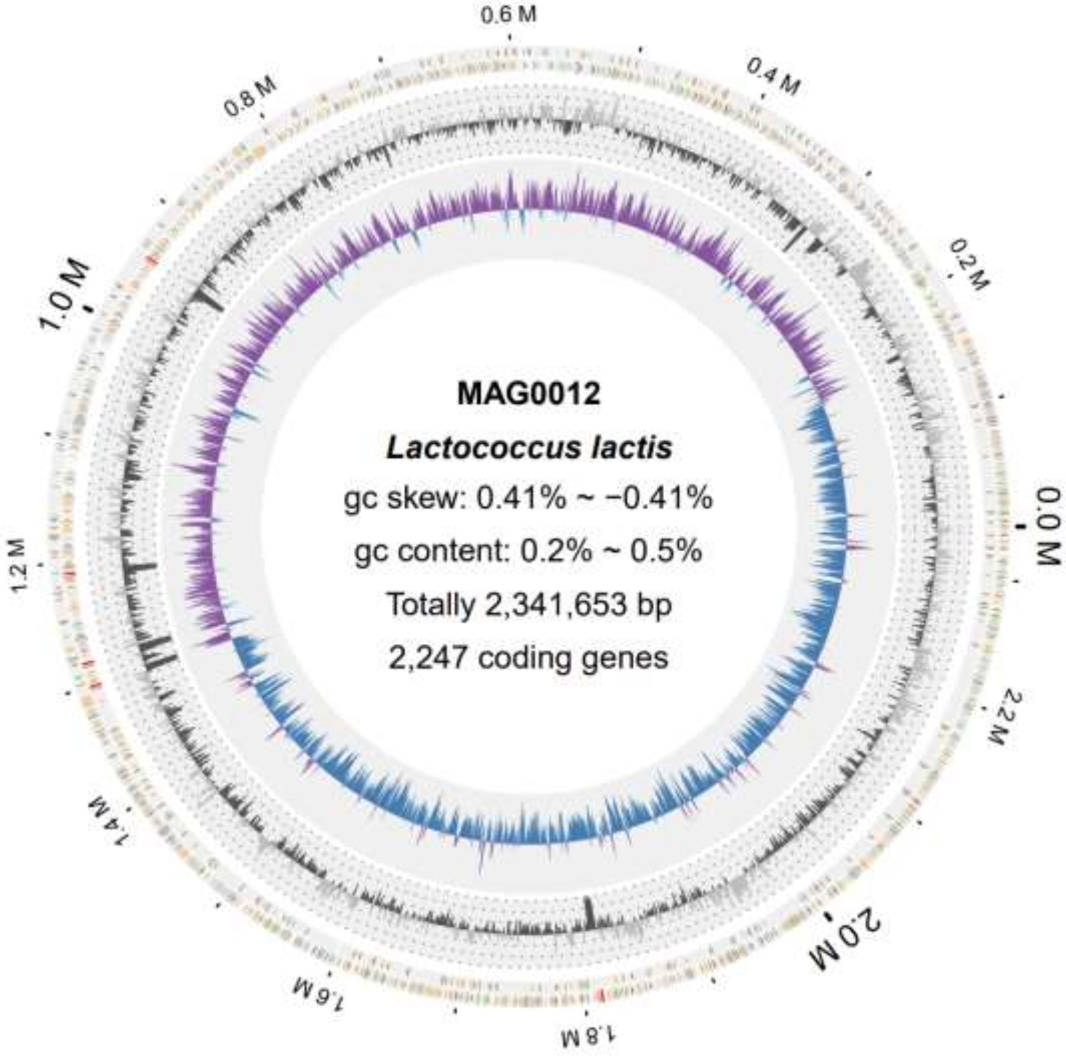

MAG0012
Lactococcus lactis
gc skew: 0.41% ~ −0.41%
gc content: 0.2% ~ 0.5%
Totally 2,341,653 bp
2,247 coding genes
0.0 M
0.2 M
0.4 M
0.6 M
0.8 M
1.0 M
1.2 M
1.4 M
1.6 M
1.8 M
2.0 M
2.2 M

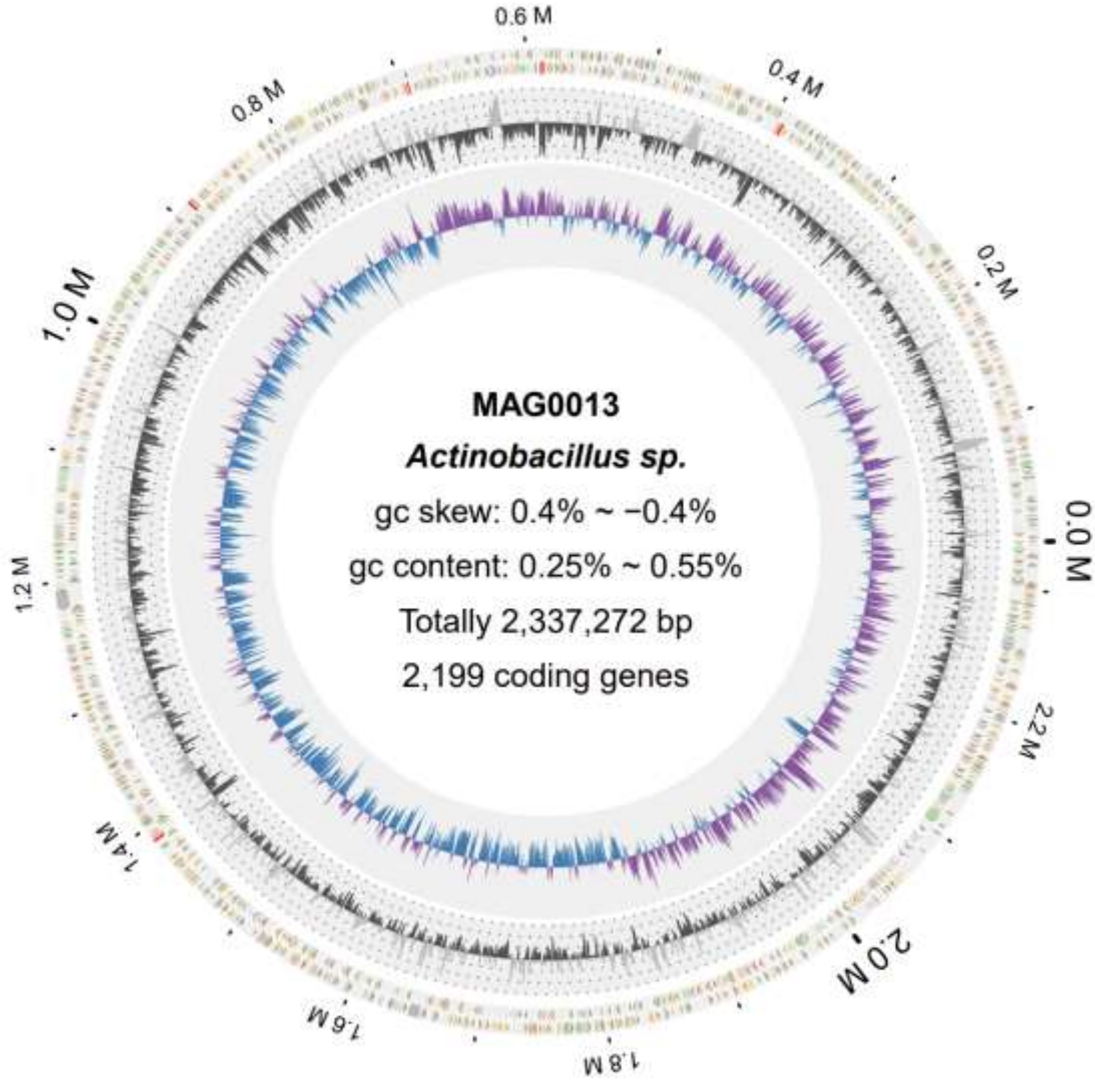

0.6 M
0.4 M
0.2 M
0.0 M
2.2 M
2.0 M
1.8 M
1.6 M
1.4 M
1.2 M
1.0 M
0.8 M
MAG0013
Actinobacillus sp.
gc skew: 0.4% ~ −0.4%
gc content: 0.25% ~ 0.55%
Totally 2,337,272 bp
2,199 coding genes

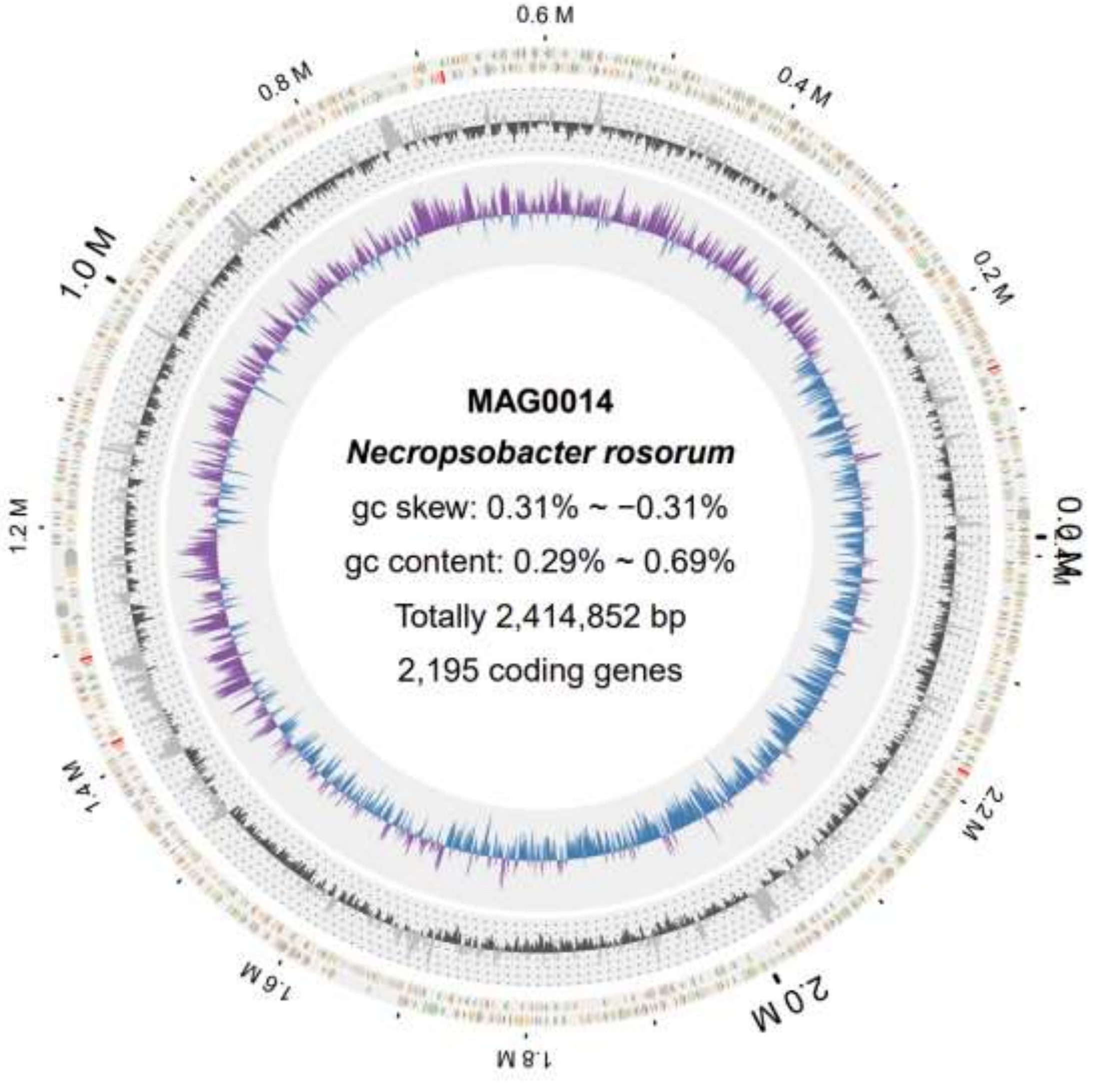
MAG0014
Necropsobacter rosorum
gc skew: 0.31% ~ −0.31%
gc content: 0.29% ~ 0.69%
Totally 2,414,852 bp
2,195 coding genes
0.0 M
0.2 M
0.4 M
0.6 M
0.8 M
1.0 M
1.2 M
1.4 M
1.6 M
1.8 M
2.0 M
2.2 M

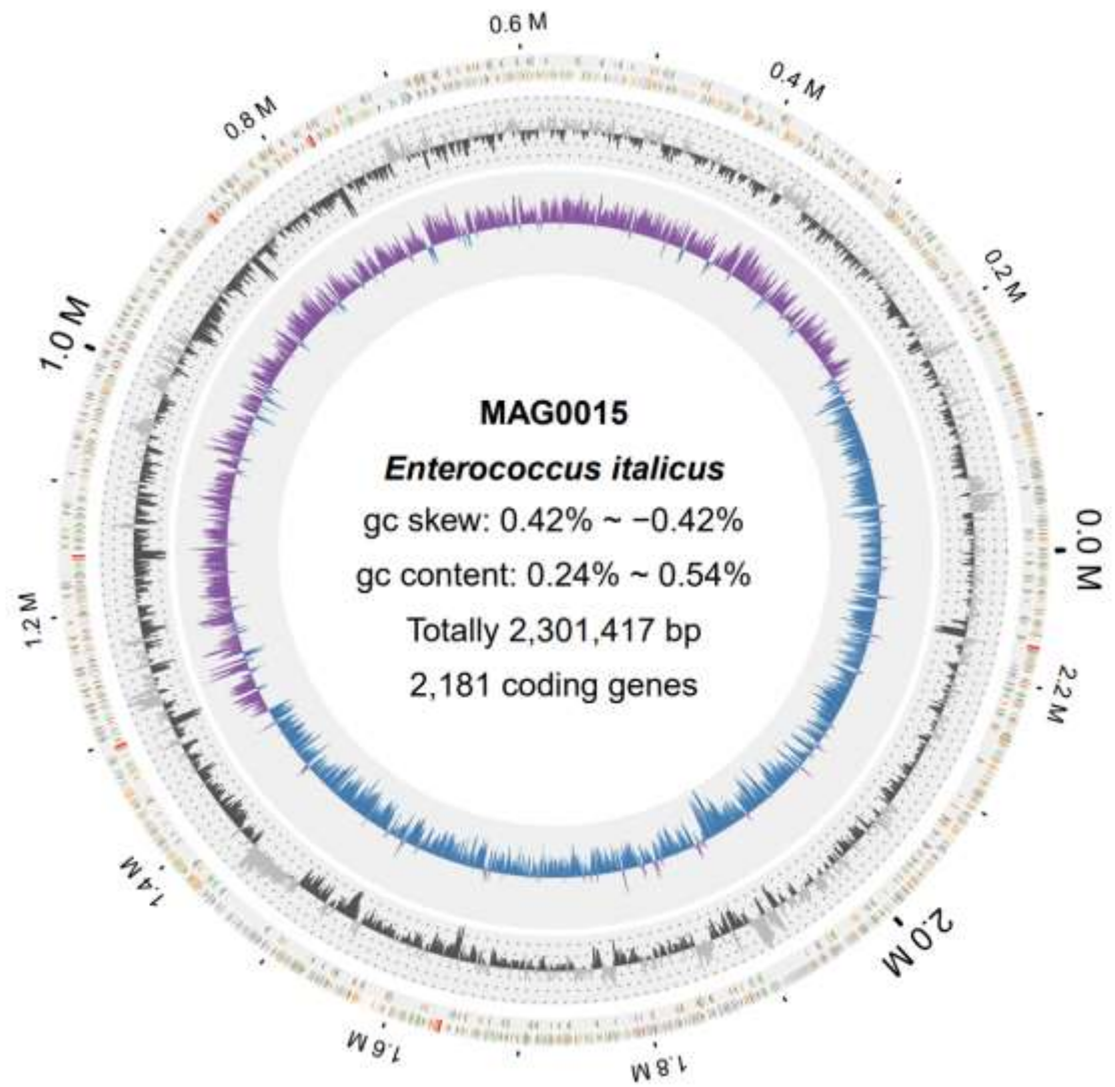
MAG0015
Enterococcus italicus
gc skew: 0.42% ~ −0.42%
gc content: 0.24% ~ 0.54%
Totally 2,301,417 bp
2,181 coding genes
0.0 M
0.2 M
0.4 M
0.6 M
0.8 M
1.0 M
1.2 M
1.4 M
1.6 M
1.8 M
2.0 M
2.2 M

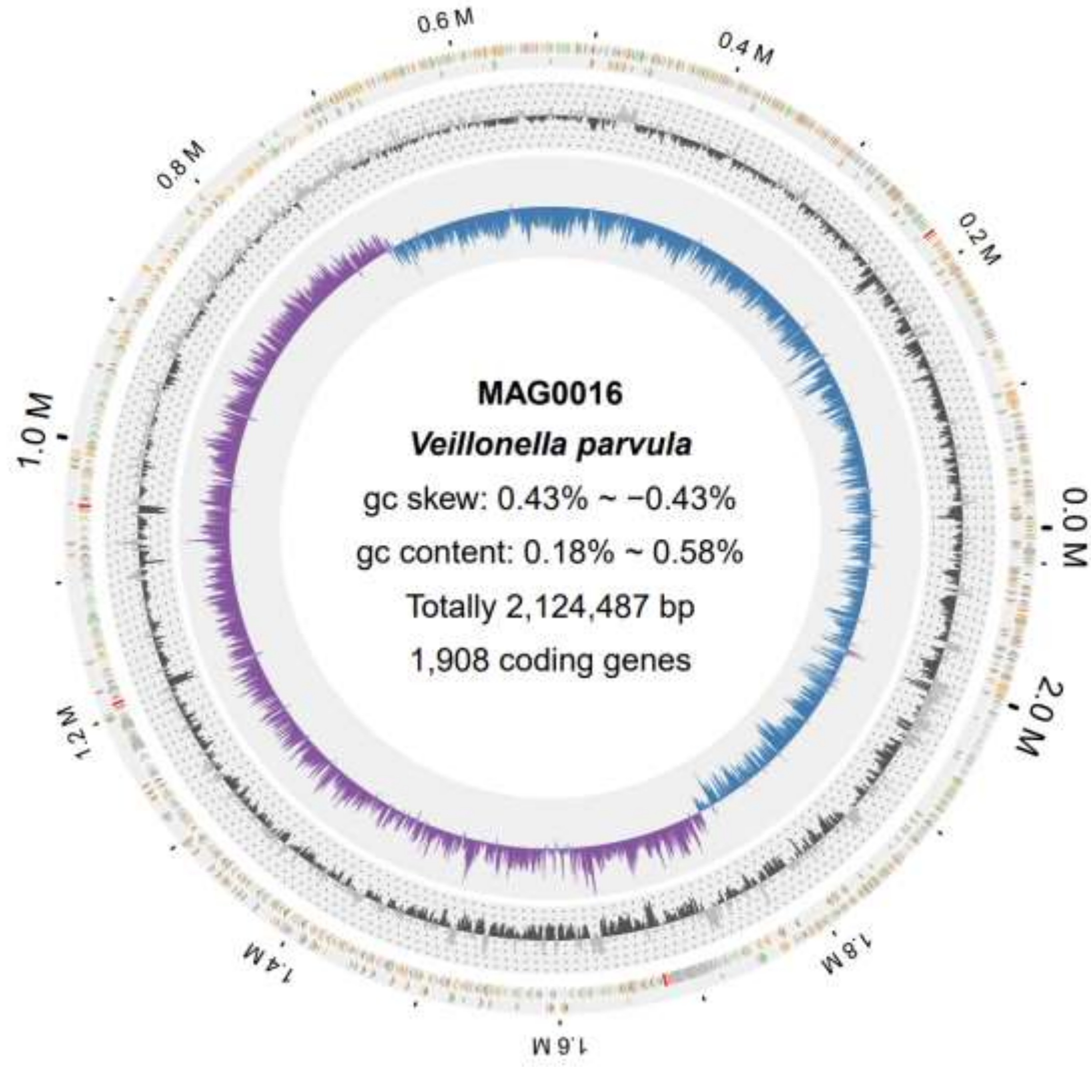

MAG0016
Veillonella parvula
gc skew: 0.43% ~ −0.43%
gc content: 0.18% ~ 0.58%
Totally 2,124,487 bp
1,908 coding genes
0.0 M
0.2 M
0.4 M
0.6 M
0.8 M
1.0 M
1.2 M
1.4 M
1.6 M
1.8 M
2.0 M

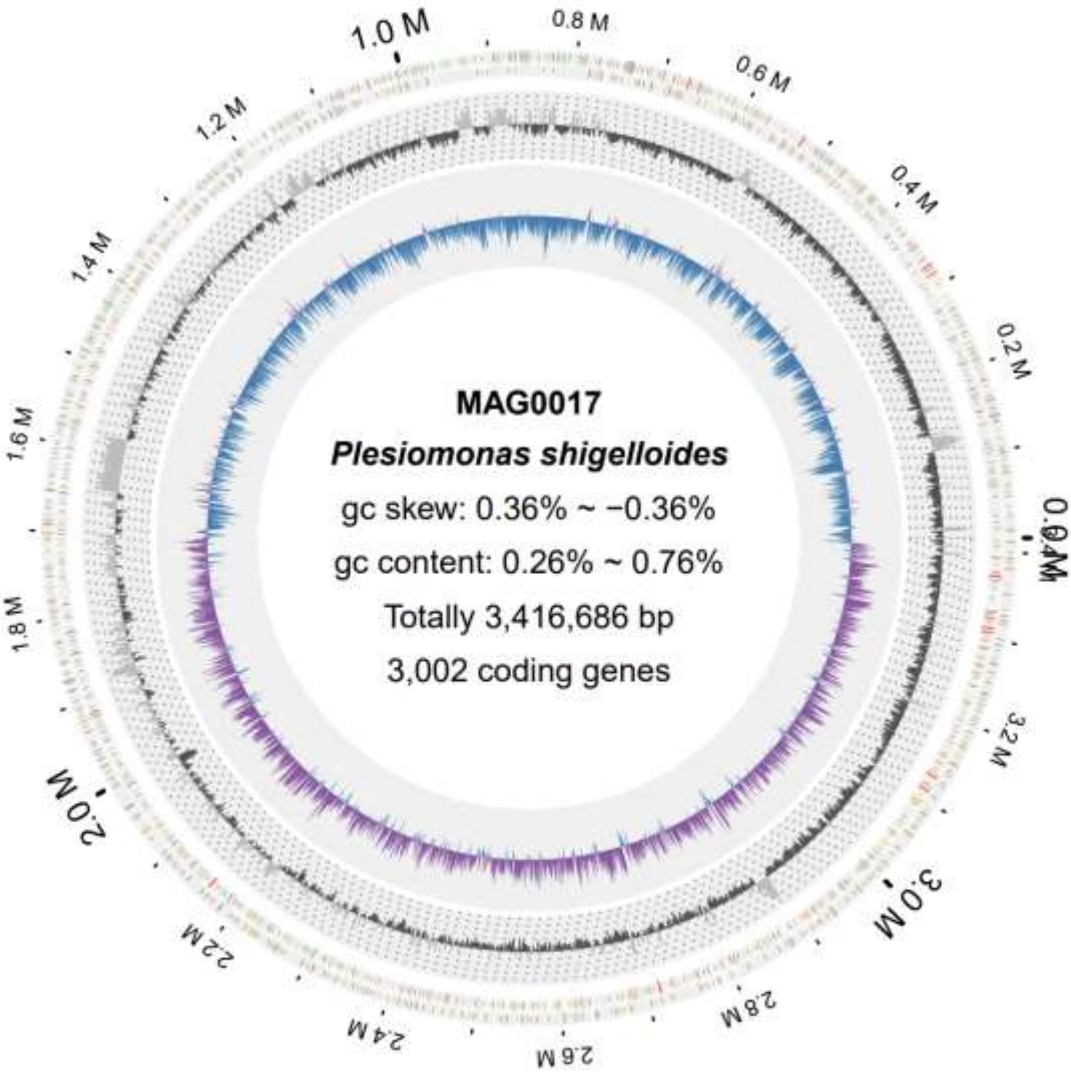

MAG0017
Plesiomonas shigelloides
gc skew: 0.36% ~ −0.36%
gc content: 0.26% ~ 0.76%
Totally 3,416,686 bp
3,002 coding genes
1.0 M
0.8 M
0.6 M
0.4 M
0.2 M
1.2 M
1.4 M
1.6 M
1.8 M
2.0 M
2.2 M
2.4 M
2.6 M
2.8 M
3.0 M
3.2 M

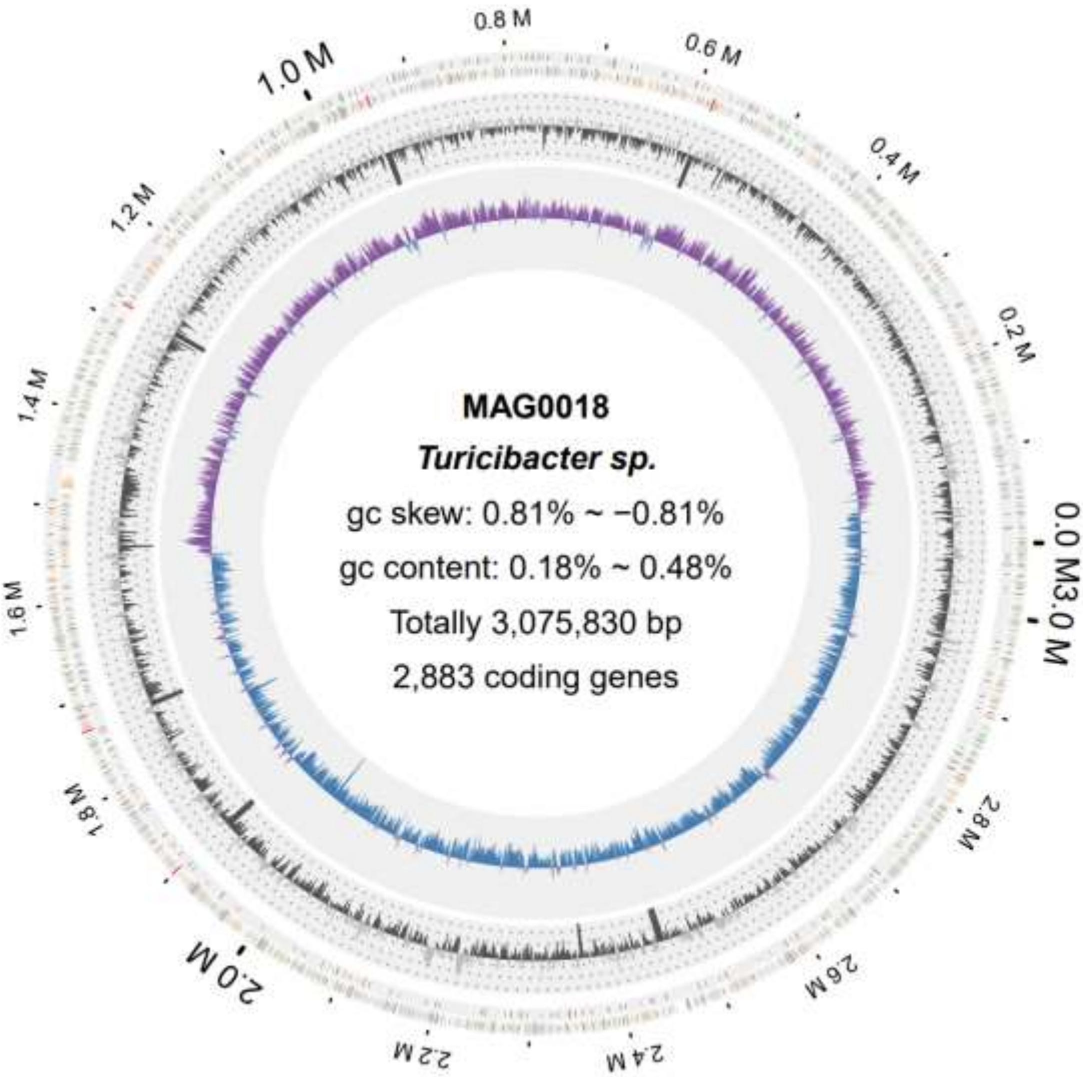

MAG0018
Turicibacter sp.
gc skew: 0.81% ~ −0.81%
gc content: 0.18% ~ 0.48%
Totally 3,075,830 bp
2,883 coding genes
0.0 M
0.2 M
0.4 M
0.6 M
0.8 M
1.0 M
1.2 M
1.4 M
1.6 M
1.8 M
2.0 M
2.2 M
2.4 M
2.6 M
2.8 M
3.0 M

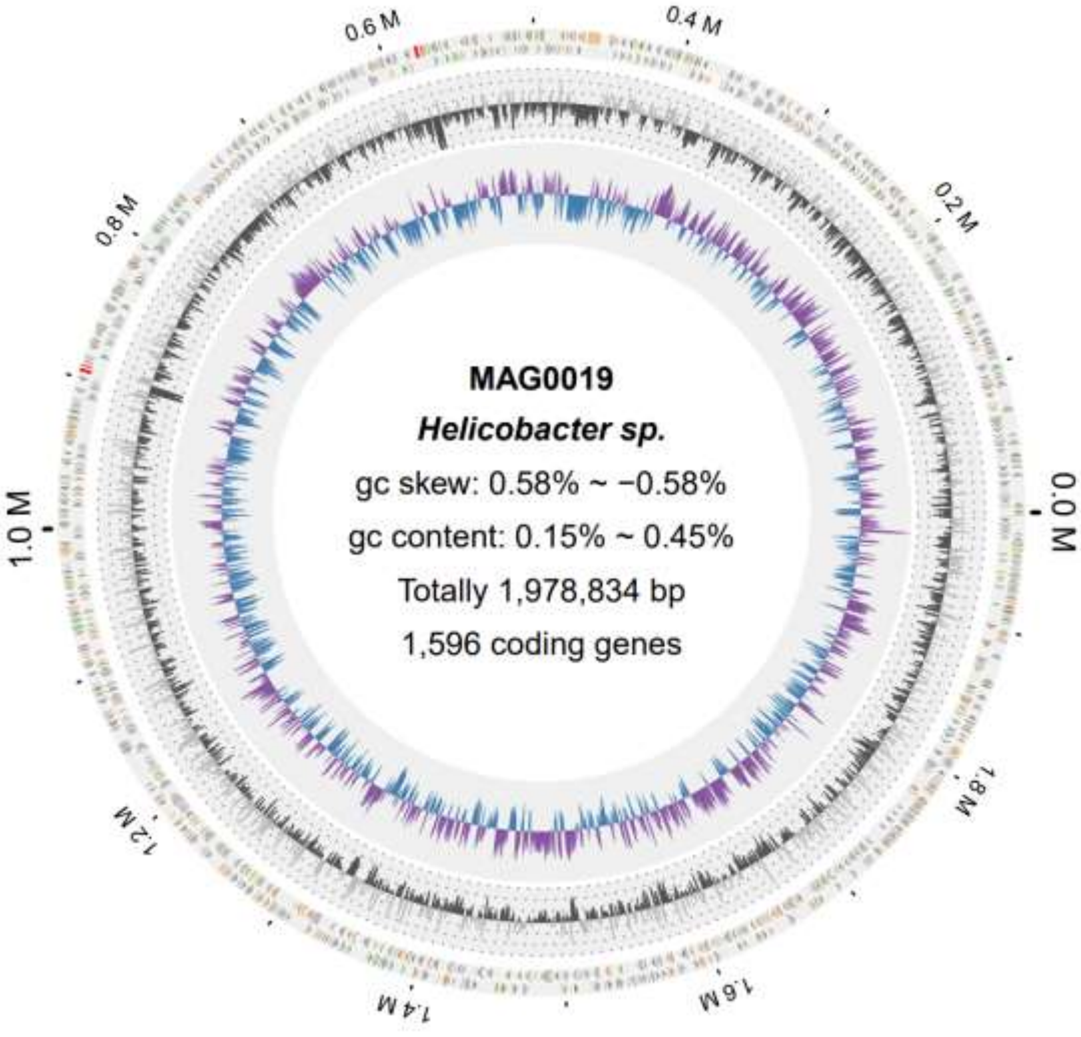
0.6 M
0.4 M
0.2 M
0.8 M
MAG0019
Helicobacter sp.
gc skew: 0.58% ~ −0.58%
gc content: 0.15% ~ 0.45%
Totally 1,978,834 bp
1,596 coding genes
1.0 M
0.0 M
1.2 M
1.8 M
1.4 M
1.6 M

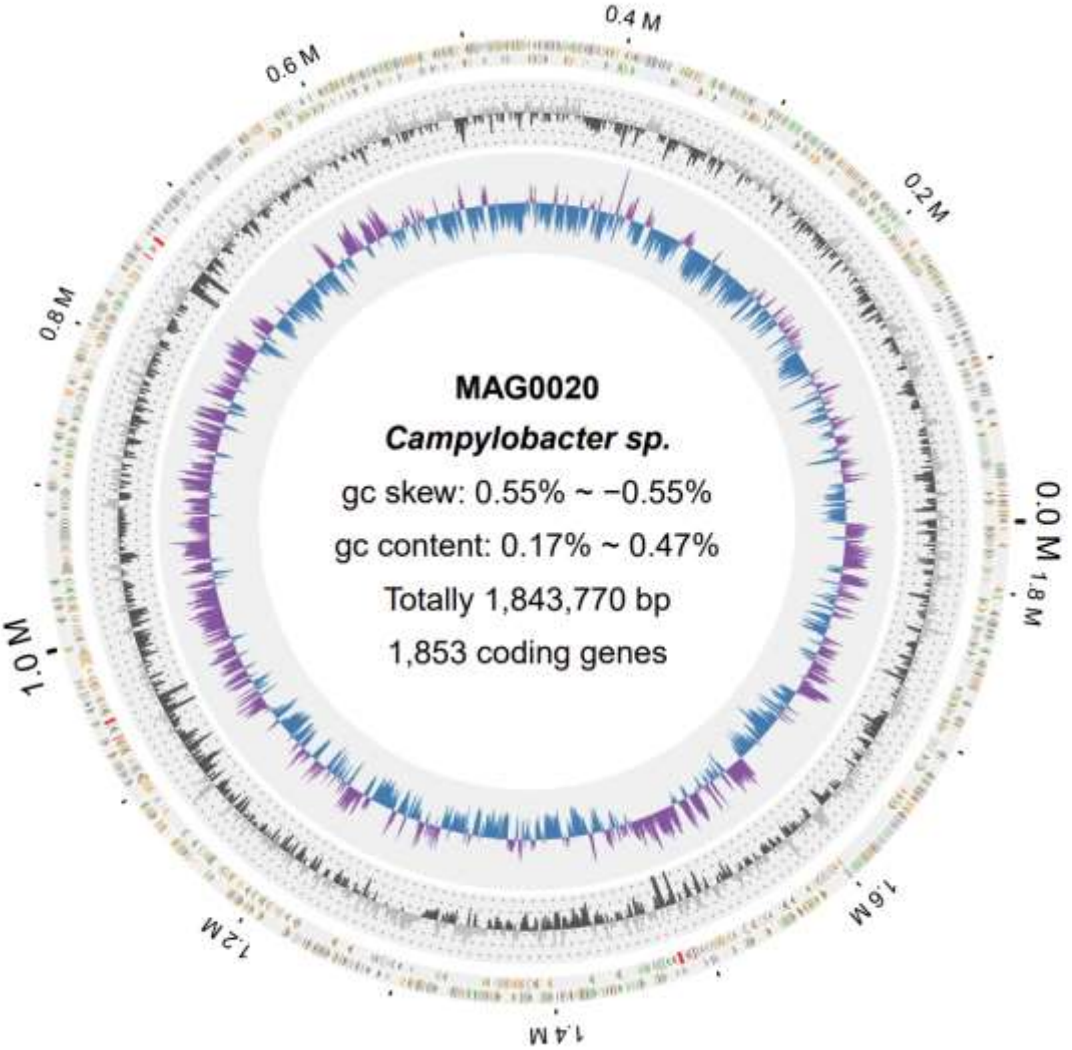
0.4 M
0.2 M
0.6 M
0.8 M
0.0 M
1.8 M
1.0 M
1.2 M
1.4 M
1.6 M
MAG0020
Campylobacter sp.
gc skew: 0.55% ~ −0.55%
gc content: 0.17% ~ 0.47%
Totally 1,843,770 bp
1,853 coding genes

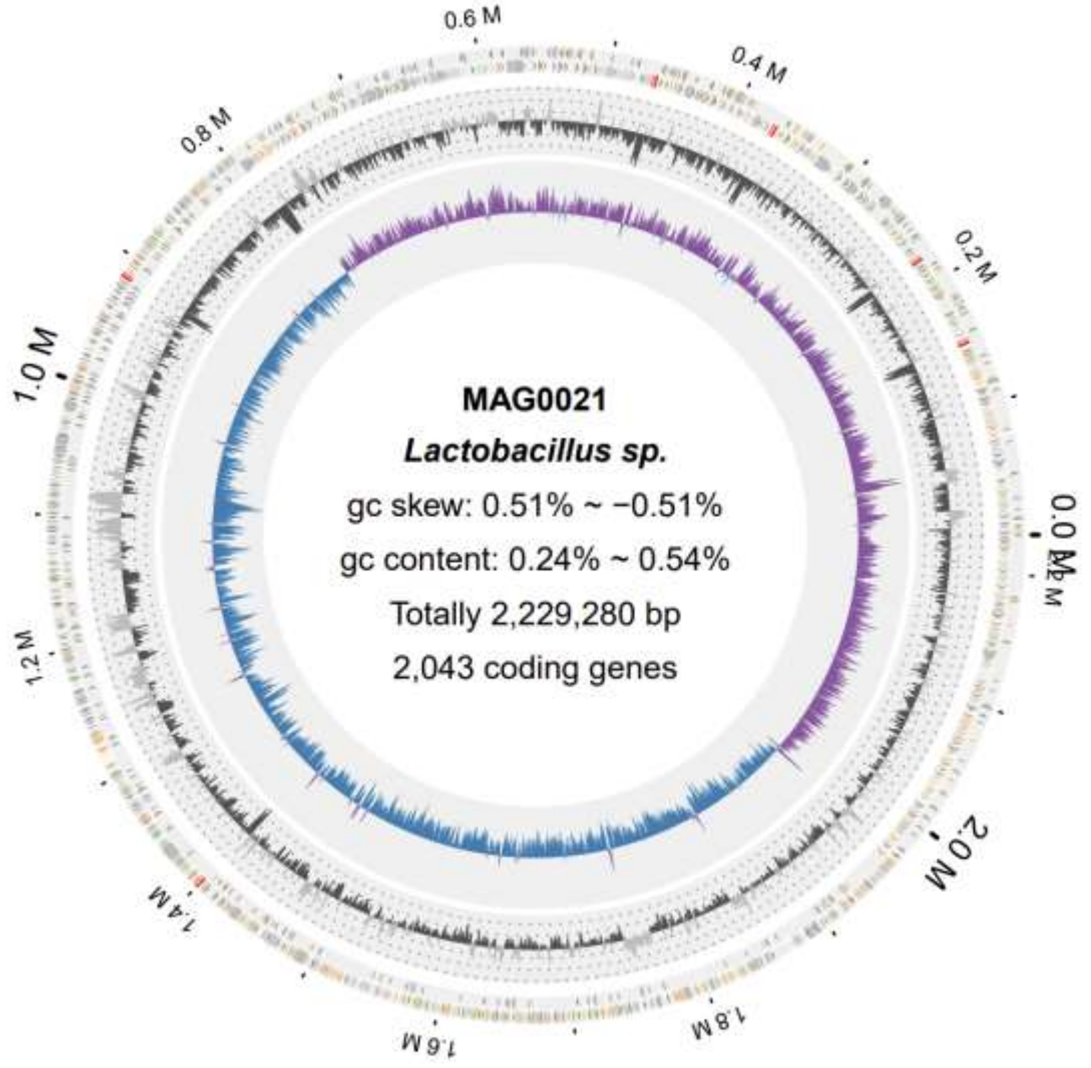

MAG0021
Lactobacillus sp.
gc skew: 0.51% ~ −0.51%
gc content: 0.24% ~ 0.54%
Totally 2,229,280 bp
2,043 coding genes
0.0 M
0.2 M
0.4 M
0.6 M
0.8 M
1.0 M
1.2 M
1.4 M
1.6 M
1.8 M
2.0 M

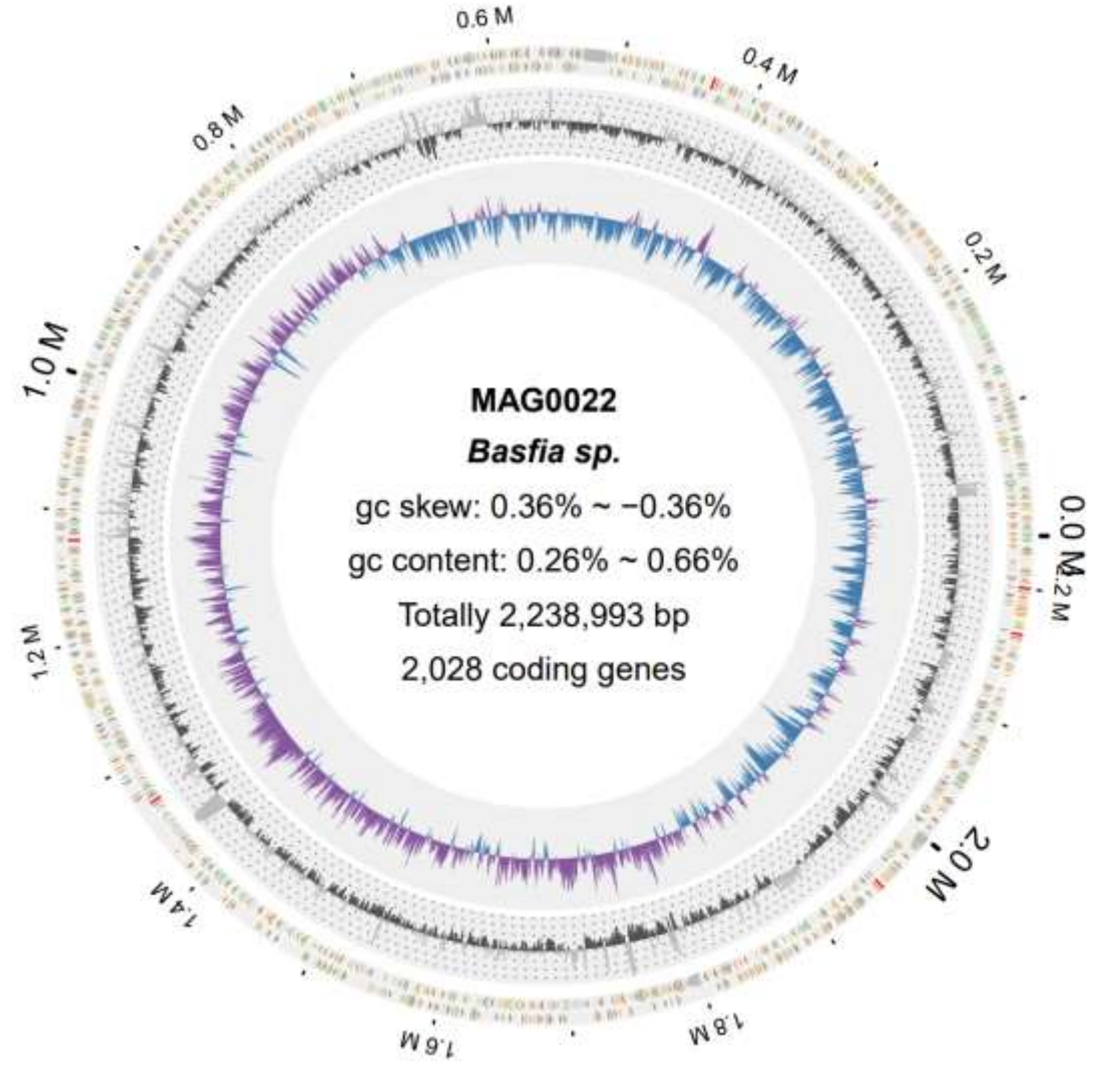

MAG0022
Basfia sp.
gc skew: 0.36% ~ −0.36%
gc content: 0.26% ~ 0.66%
Totally 2,238,993 bp
2,028 coding genes
0.0 M
0.2 M
0.4 M
0.6 M
0.8 M
1.0 M
1.2 M
1.4 M
1.6 M
1.8 M
2.0 M

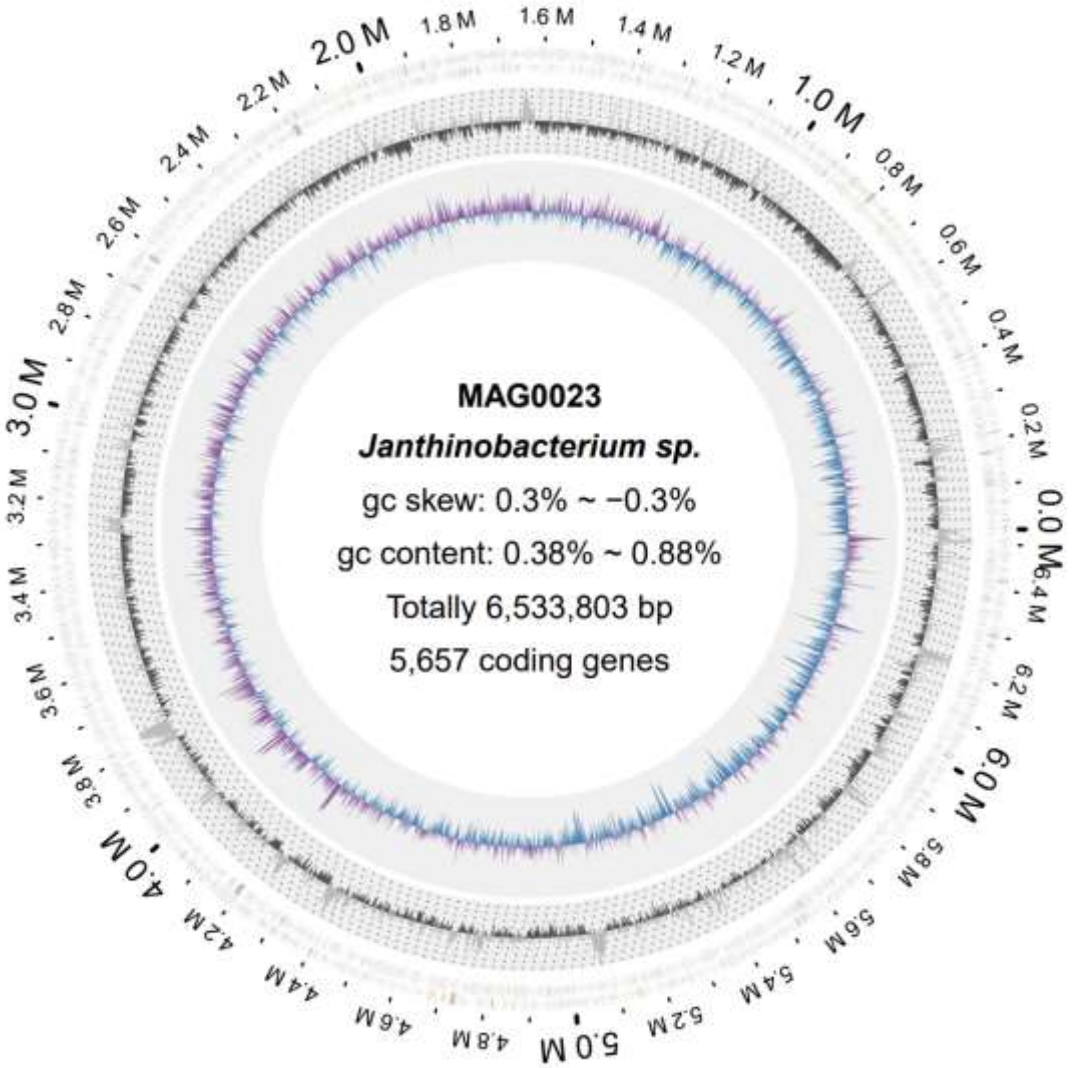
MAG0023
Janthinobacterium sp.
gc skew: 0.3% ~ −0.3%
gc content: 0.38% ~ 0.88%
Totally 6,533,803 bp
5,657 coding genes

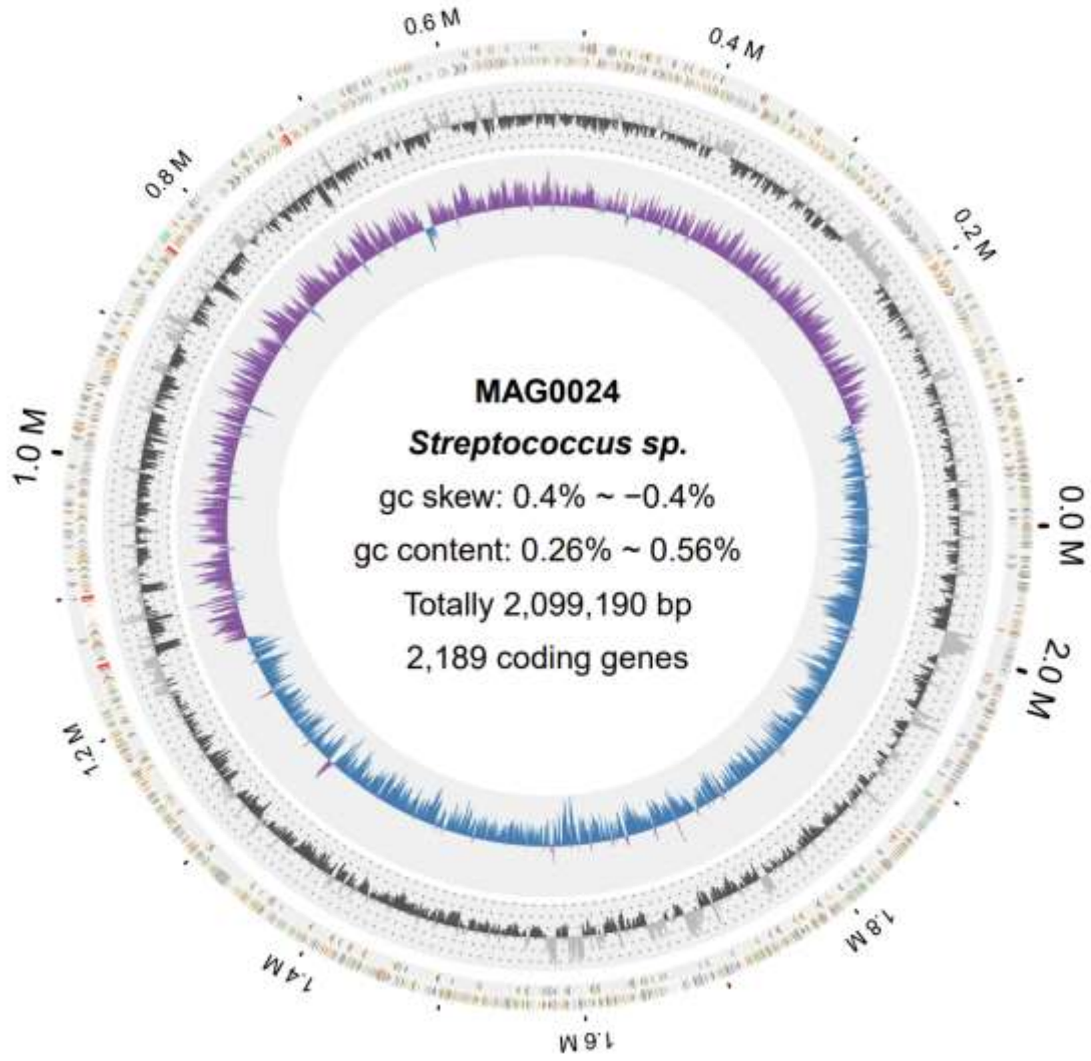
MAG0024
Streptococcus sp.
gc skew: 0.4% ~ −0.4%
gc content: 0.26% ~ 0.56%
Totally 2,099,190 bp
2,189 coding genes
0.0 M
0.2 M
0.4 M
0.6 M
0.8 M
1.0 M
1.2 M
1.4 M
1.6 M
1.8 M
2.0 M

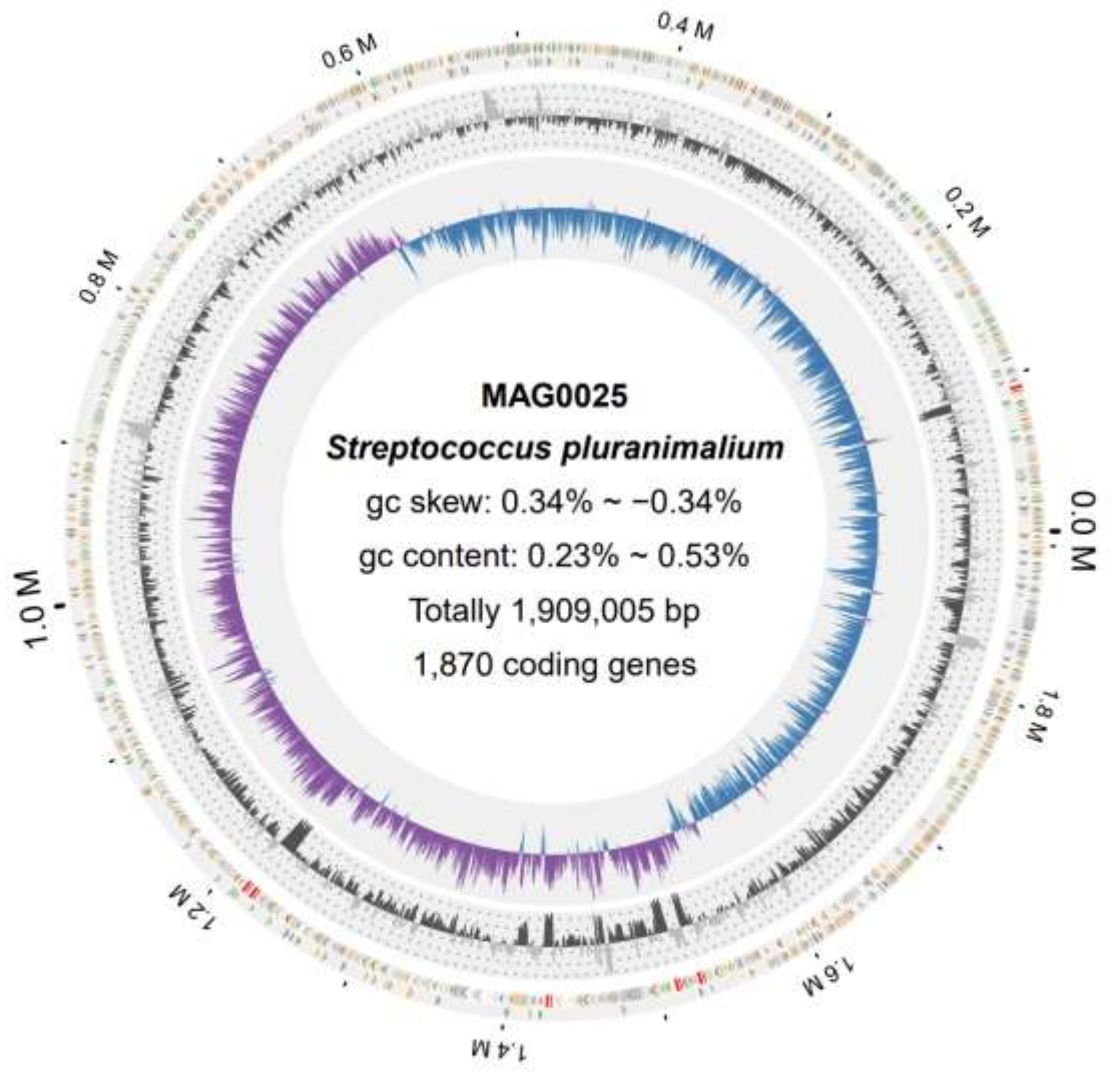

MAG0025
Streptococcus pluranimalium
gc skew: 0.34% ~ −0.34%
gc content: 0.23% ~ 0.53%
Totally 1,909,005 bp
1,870 coding genes
0.0 M
0.2 M
0.4 M
0.6 M
0.8 M
1.0 M
1.2 M
1.4 M
1.6 M
1.8 M

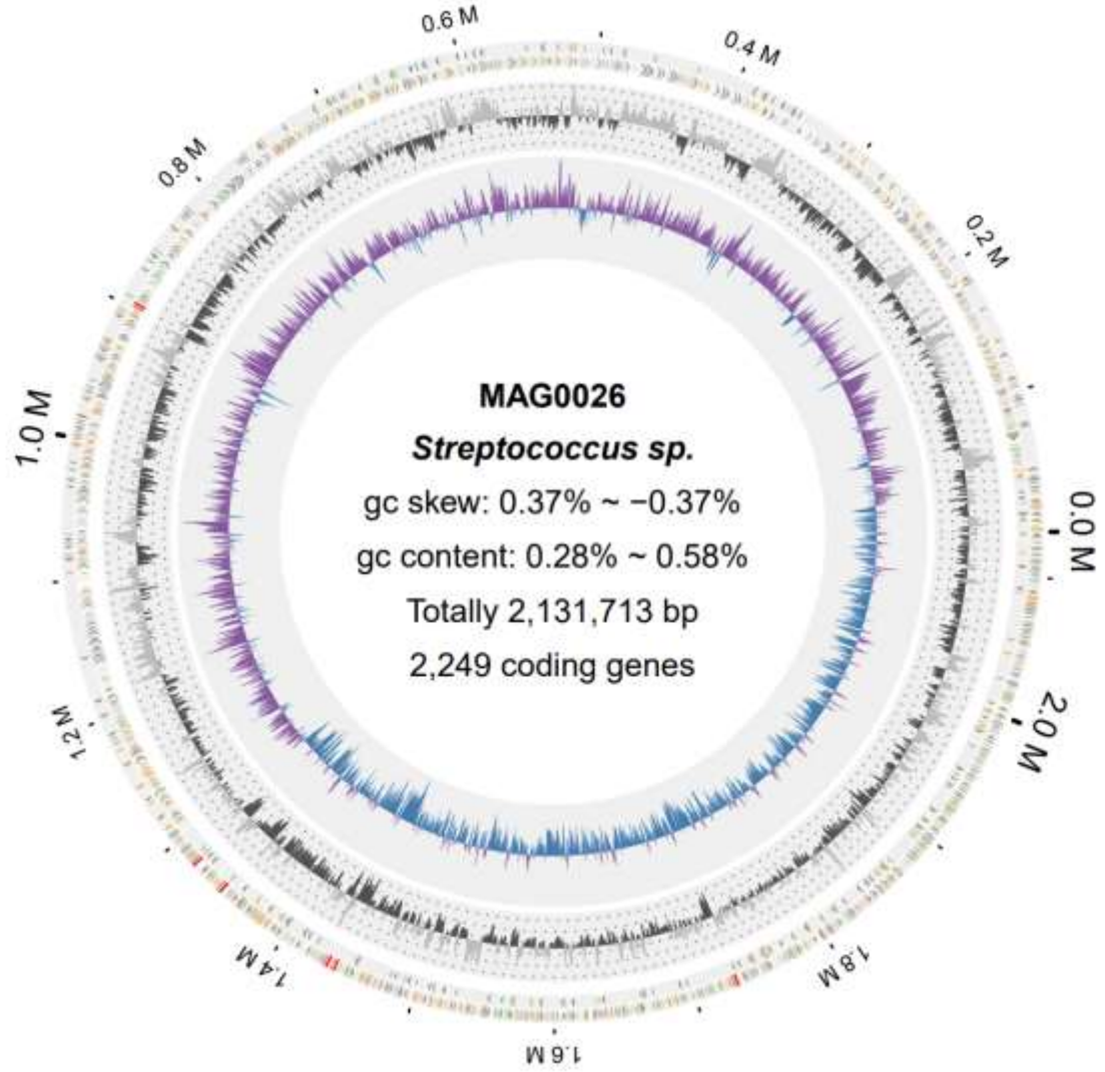
MAG0026
Streptococcus sp.
gc skew: 0.37% ~ −0.37%
gc content: 0.28% ~ 0.58%
Totally 2,131,713 bp
2,249 coding genes
0.0 M
0.2 M
0.4 M
0.6 M
0.8 M
1.0 M
1.2 M
1.4 M
1.6 M
1.8 M
2.0 M

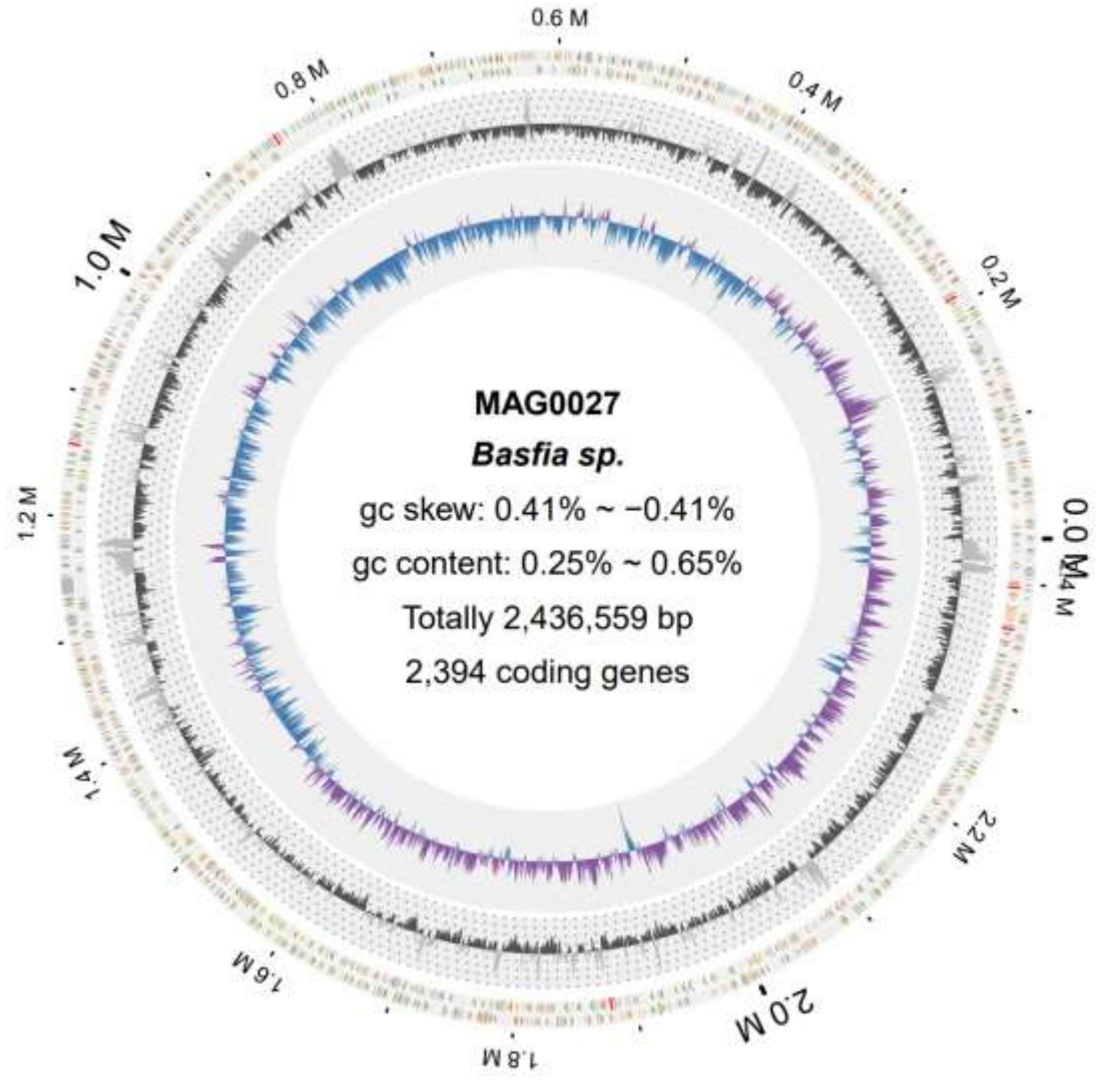
MAG0027
Basfia sp.
gc skew: 0.41% ~ −0.41%
gc content: 0.25% ~ 0.65%
Totally 2,436,559 bp
2,394 coding genes
0.0 M
0.2 M
0.4 M
0.6 M
0.8 M
1.0 M
1.2 M
1.4 M
1.6 M
1.8 M
2.0 M
2.2 M
2.4 M

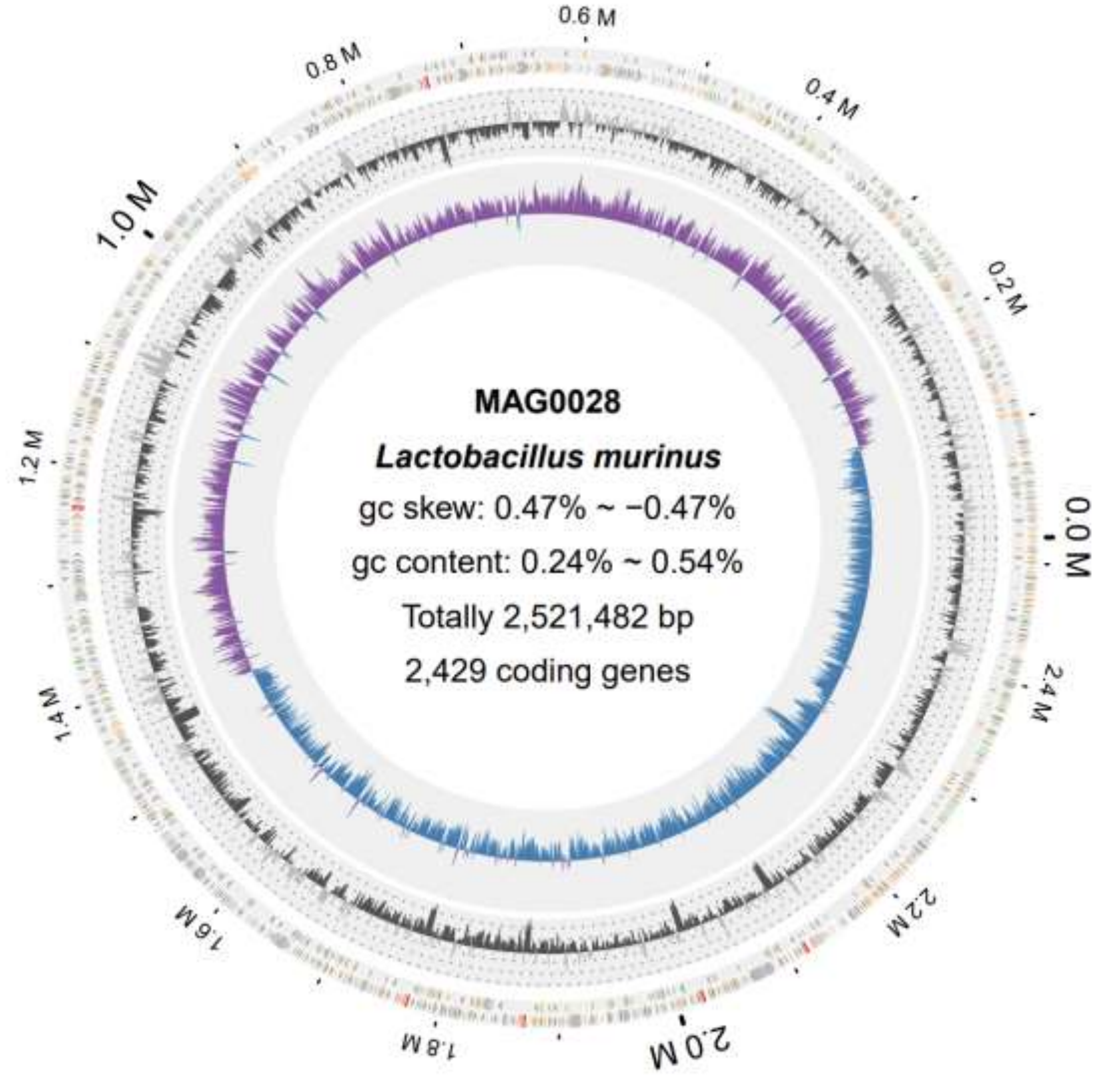
0.6 M
0.8 M
0.4 M
1.0 M
0.2 M
MAG0028
Lactobacillus murinus
gc skew: 0.47% ~ −0.47%
gc content: 0.24% ~ 0.54%
Totally 2,521,482 bp
2,429 coding genes
1.2 M
0.0 M
1.4 M
2.4 M
1.6 M
2.2 M
1.8 M
2.0 M

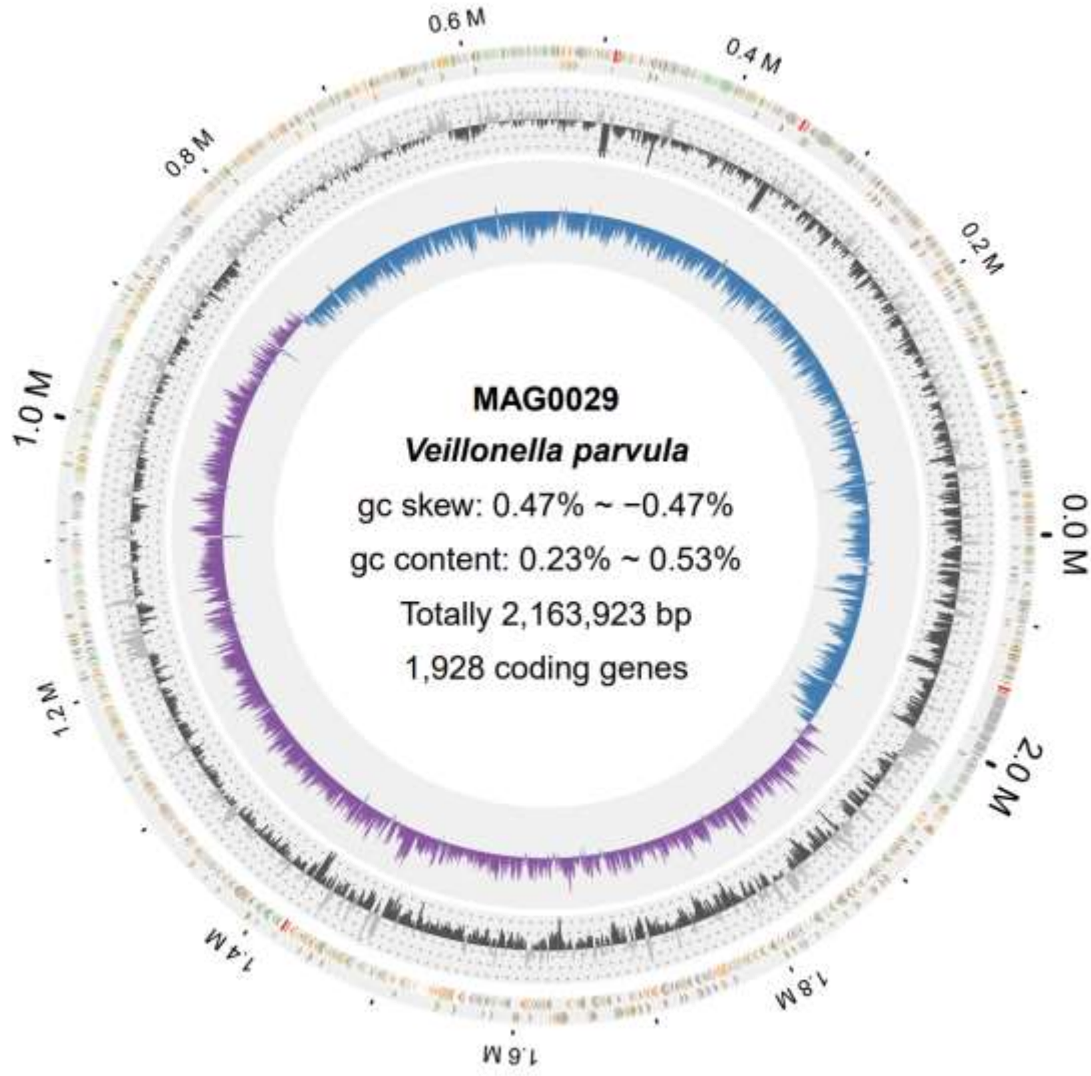
MAG0029
Veillonella parvula
gc skew: 0.47% ~ −0.47%
gc content: 0.23% ~ 0.53%
Totally 2,163,923 bp
1,928 coding genes
0.0 M
0.2 M
0.4 M
0.6 M
0.8 M
1.0 M
1.2 M
1.4 M
1.6 M
1.8 M
2.0 M

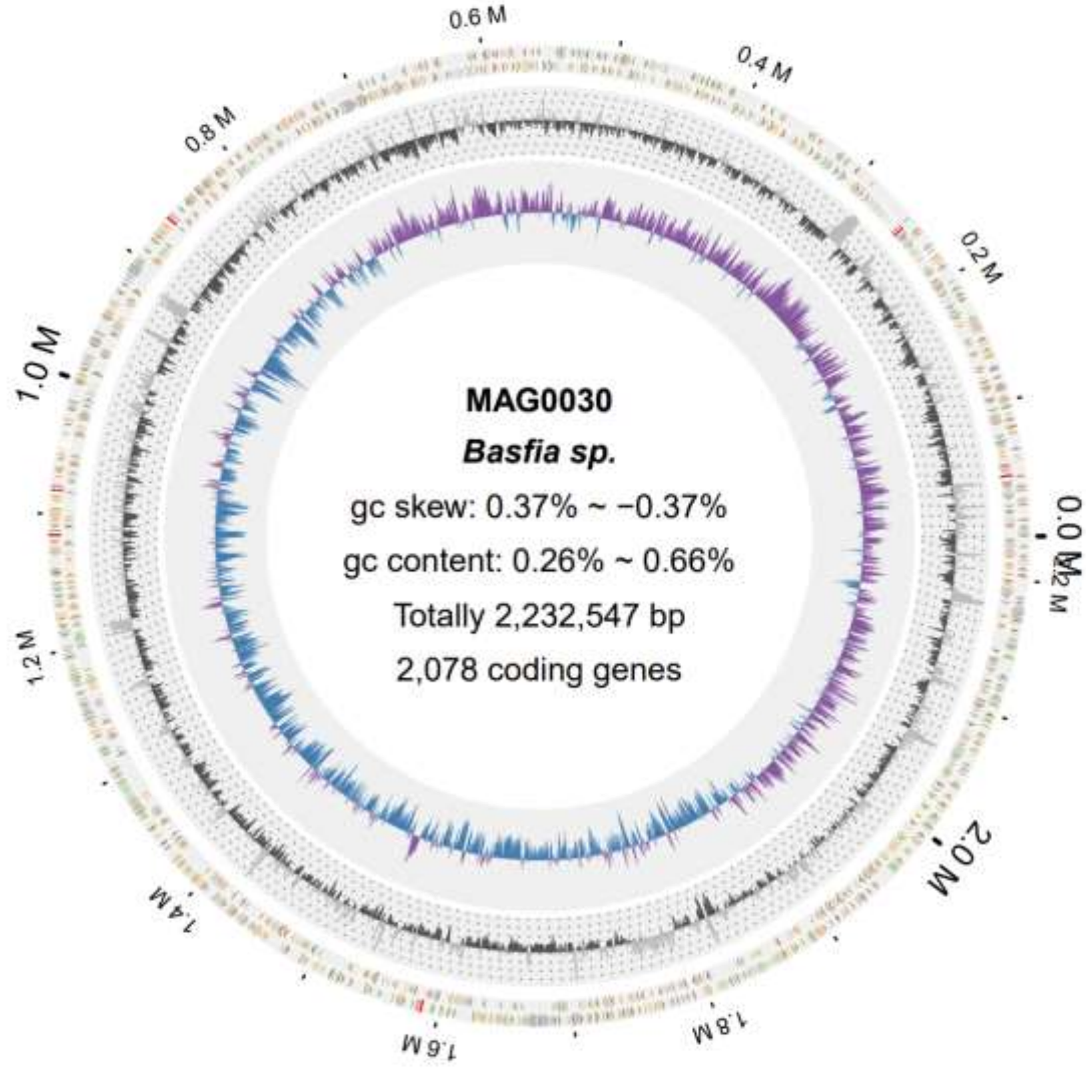

0.6 M
0.4 M
0.8 M
0.2 M
1.0 M
0.0 M
2.2 M
1.2 M
2.0 M
1.4 M
1.8 M
1.6 M
MAG0030
Basfia sp.
gc skew: 0.37% ~ −0.37%
gc content: 0.26% ~ 0.66%
Totally 2,232,547 bp
2,078 coding genes

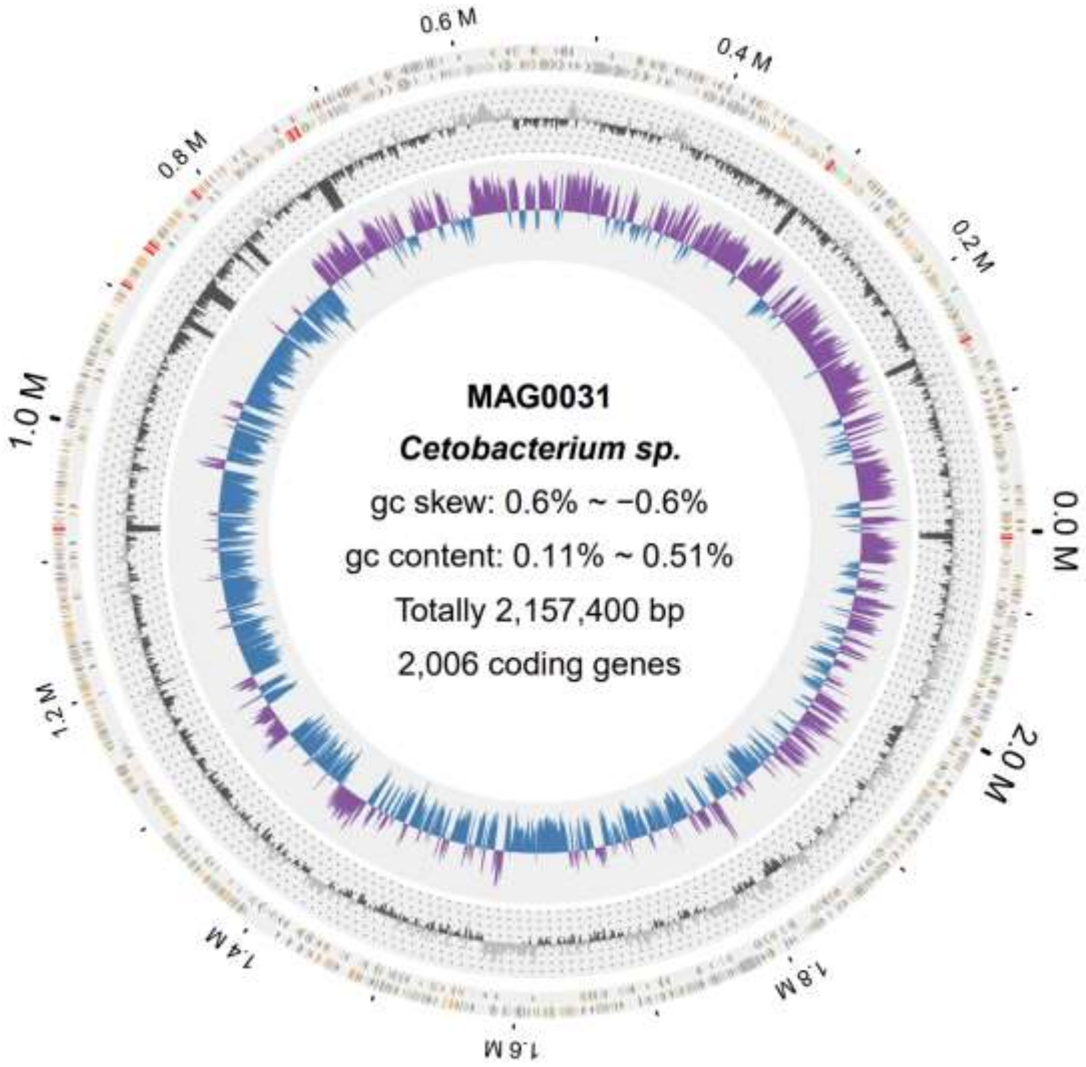
MAG0031
Cetobacterium sp.
gc skew: 0.6% ~ −0.6%
gc content: 0.11% ~ 0.51%
Totally 2,157,400 bp
2,006 coding genes
0.0 M
0.2 M
0.4 M
0.6 M
0.8 M
1.0 M
1.2 M
1.4 M
1.6 M
1.8 M
2.0 M

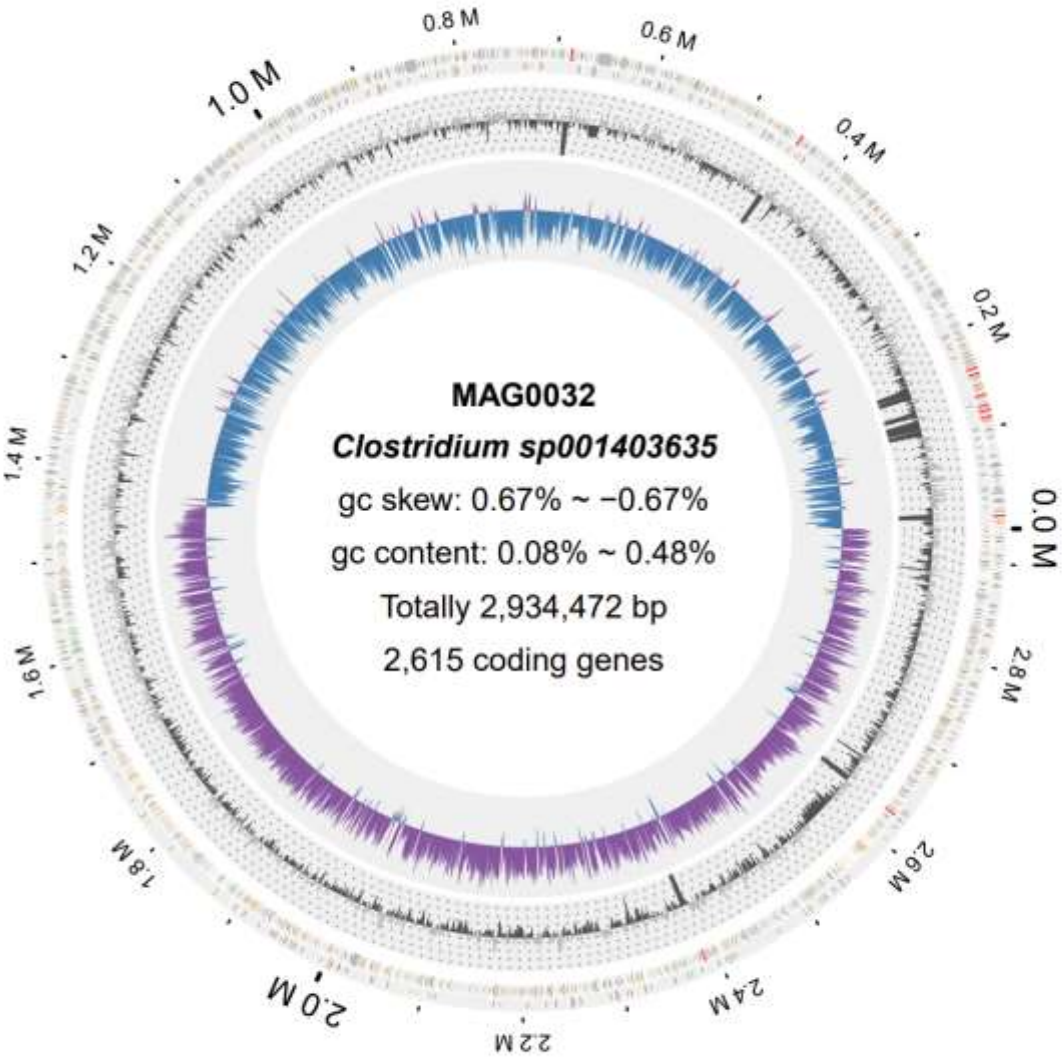
MAG0032
Clostridium sp001403635
gc skew: 0.67% ~ −0.67%
gc content: 0.08% ~ 0.48%
Totally 2,934,472 bp
2,615 coding genes
0.0 M
0.2 M
0.4 M
0.6 M
0.8 M
1.0 M
1.2 M
1.4 M
1.6 M
1.8 M
2.0 M
2.2 M
2.4 M
2.6 M
2.8 M

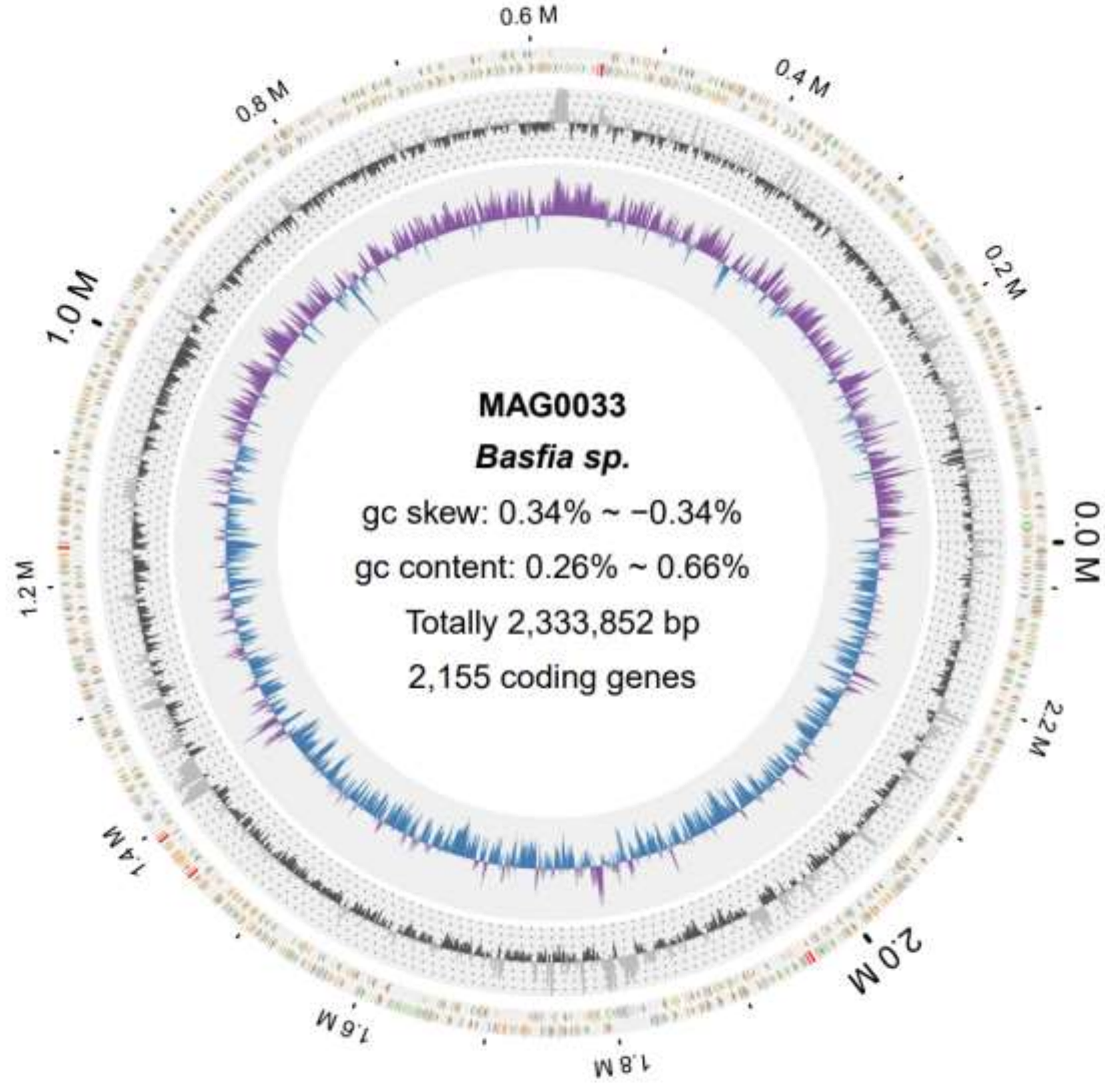
0.6 M
0.4 M
0.2 M
0.0 M
2.2 M
2.0 M
1.8 M
1.6 M
1.4 M
1.2 M
1.0 M
0.8 M
MAG0033
Basfia sp.
gc skew: 0.34% ~ −0.34%
gc content: 0.26% ~ 0.66%
Totally 2,333,852 bp
2,155 coding genes

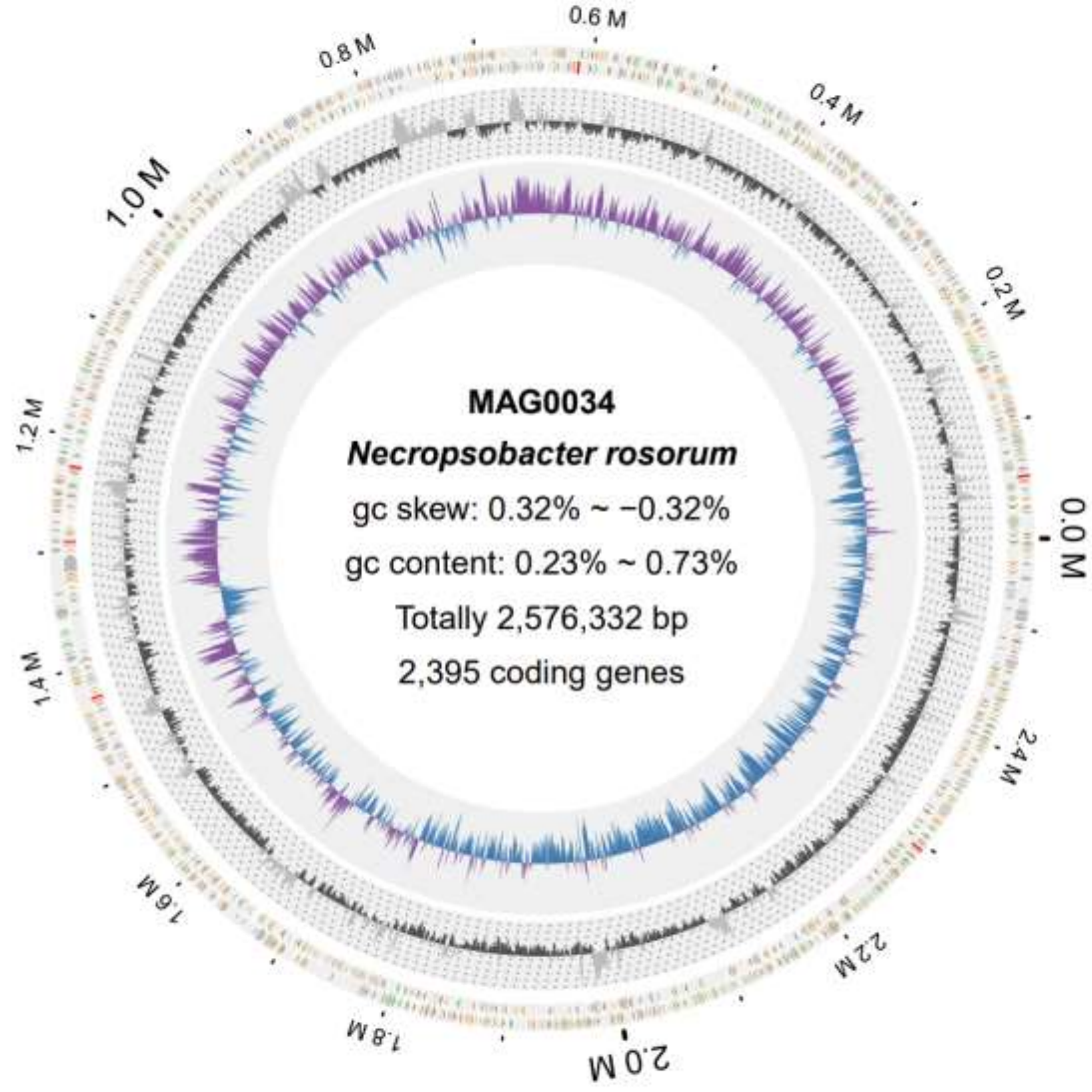
0.6 M
0.4 M
0.2 M
0.0 M
2.4 M
2.2 M
2.0 M
1.8 M
1.6 M
1.4 M
1.2 M
1.0 M
0.8 M
MAG0034
Necropsobacter rosorum
gc skew: 0.32% ~ −0.32%
gc content: 0.23% ~ 0.73%
Totally 2,576,332 bp
2,395 coding genes

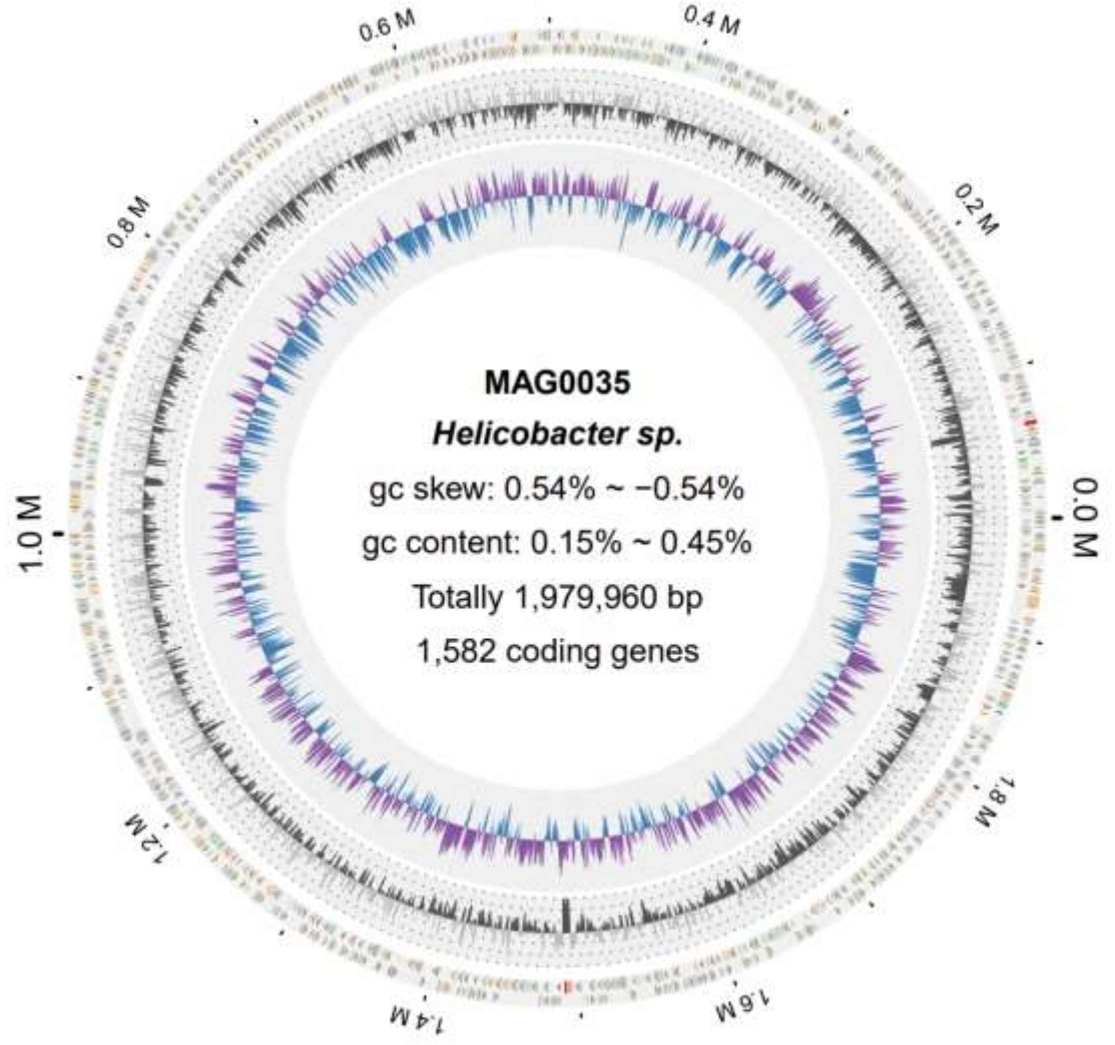

MAG0035
Helicobacter sp.
gc skew: 0.54% ~ −0.54%
gc content: 0.15% ~ 0.45%
Totally 1,979,960 bp
1,582 coding genes
0.0 M
0.2 M
0.4 M
0.6 M
0.8 M
1.0 M
1.2 M
1.4 M
1.6 M
1.8 M

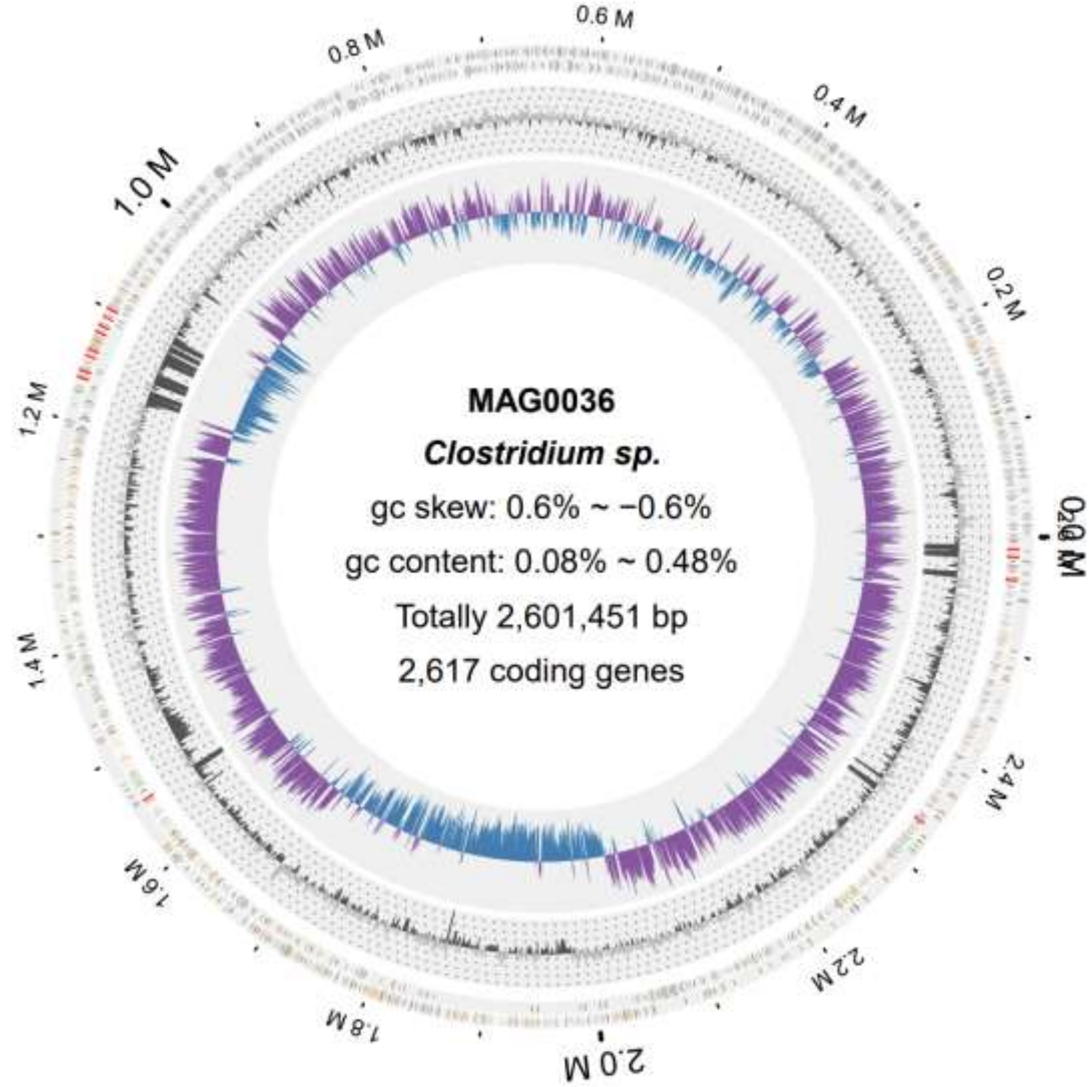
MAG0036
Clostridium sp.
gc skew: 0.6% ~ −0.6%
gc content: 0.08% ~ 0.48%
Totally 2,601,451 bp
2,617 coding genes
0.0 M
0.2 M
0.4 M
0.6 M
0.8 M
1.0 M
1.2 M
1.4 M
1.6 M
1.8 M
2.0 M
2.2 M
2.4 M

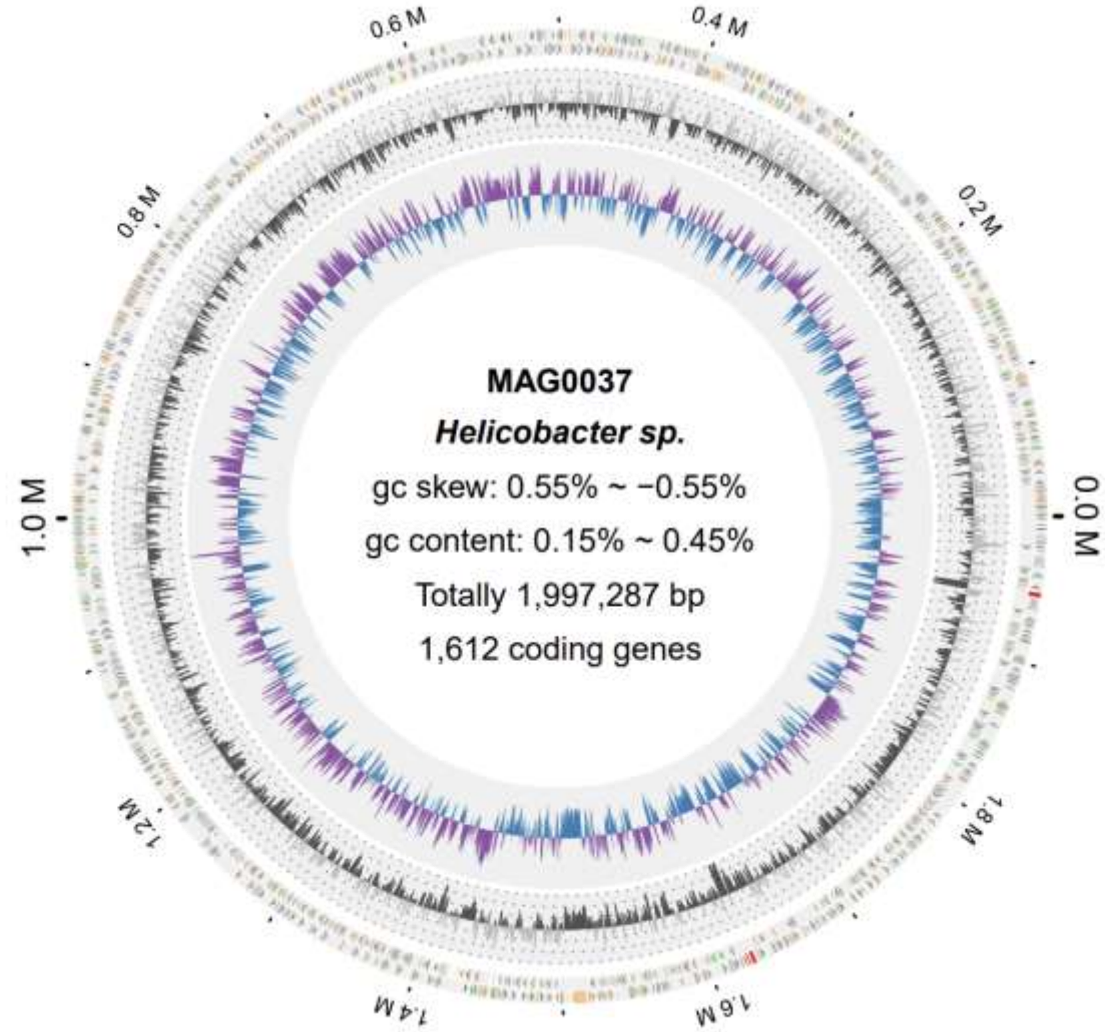

MAG0037
Helicobacter sp.
gc skew: 0.55% ~ −0.55%
gc content: 0.15% ~ 0.45%
Totally 1,997,287 bp
1,612 coding genes
0.0 M
0.2 M
0.4 M
0.6 M
0.8 M
1.0 M
1.2 M
1.4 M
1.6 M
1.8 M

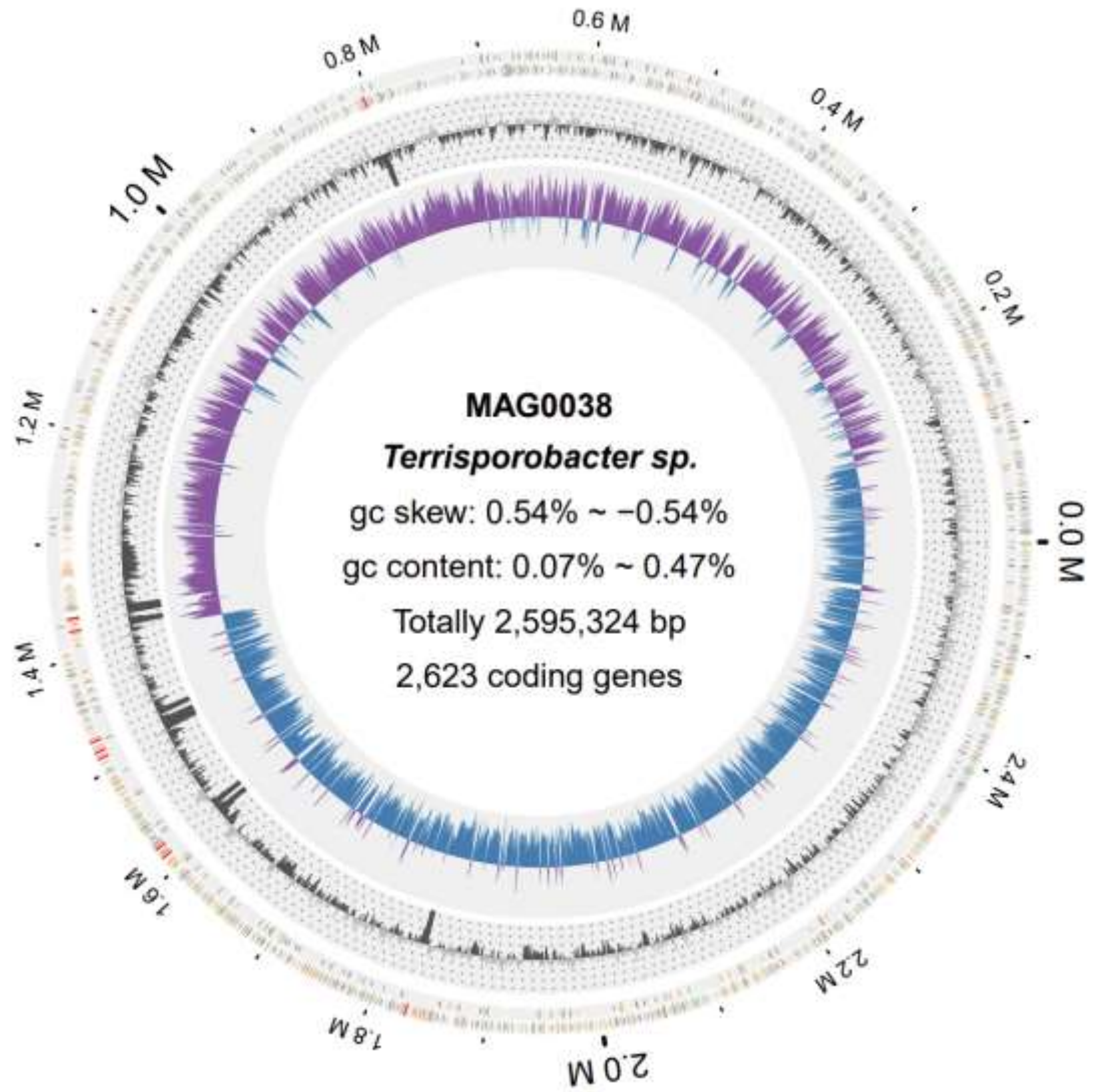
MAG0038
Terrisporobacter sp.
gc skew: 0.54% ~ −0.54%
gc content: 0.07% ~ 0.47%
Totally 2,595,324 bp
2,623 coding genes
0.0 M
0.2 M
0.4 M
0.6 M
0.8 M
1.0 M
1.2 M
1.4 M
1.6 M
1.8 M
2.0 M
2.2 M
2.4 M

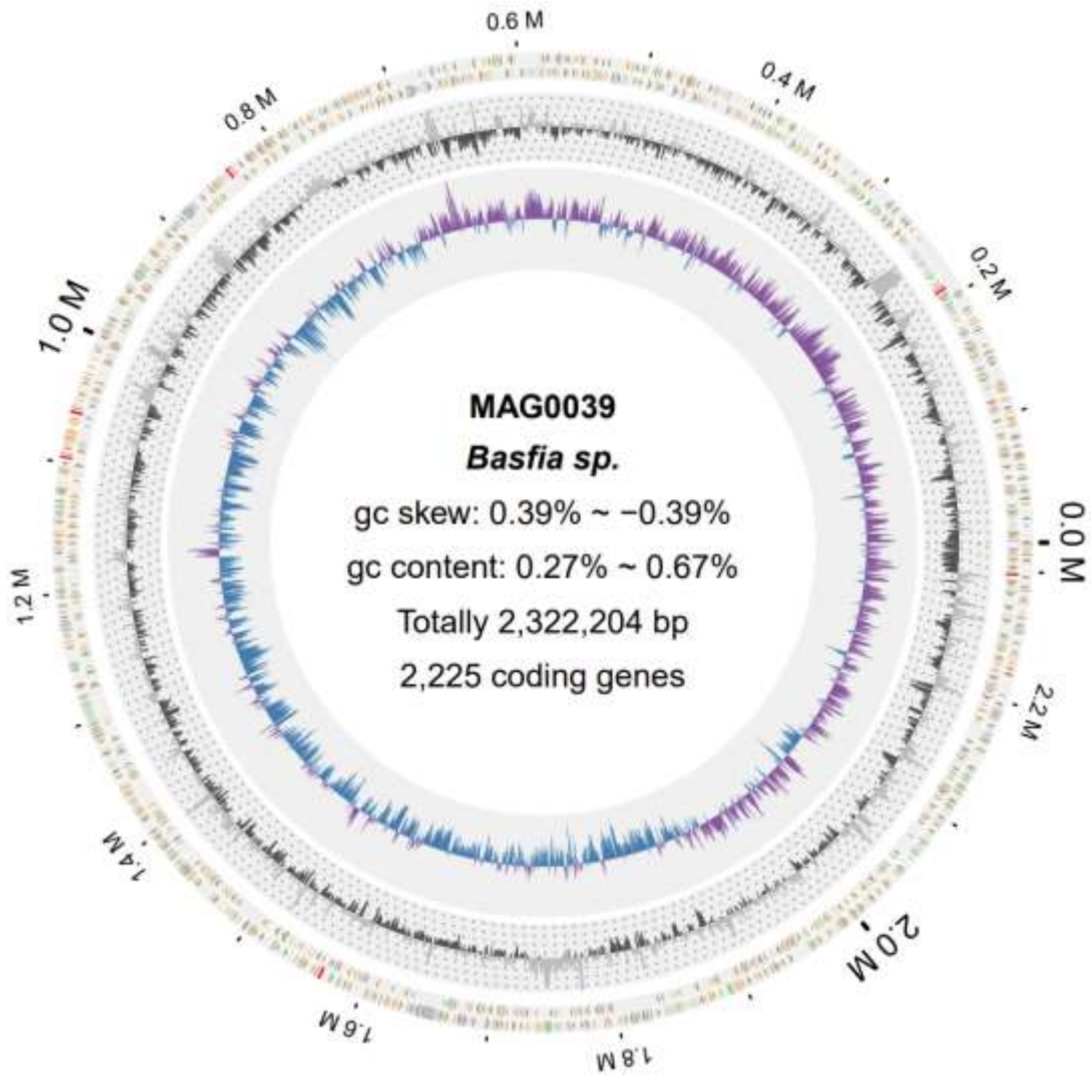

MAG0039
Basfia sp.
gc skew: 0.39% ~ −0.39%
gc content: 0.27% ~ 0.67%
Totally 2,322,204 bp
2,225 coding genes
0.0 M
0.2 M
0.4 M
0.6 M
0.8 M
1.0 M
1.2 M
1.4 M
1.6 M
1.8 M
2.0 M
2.2 M

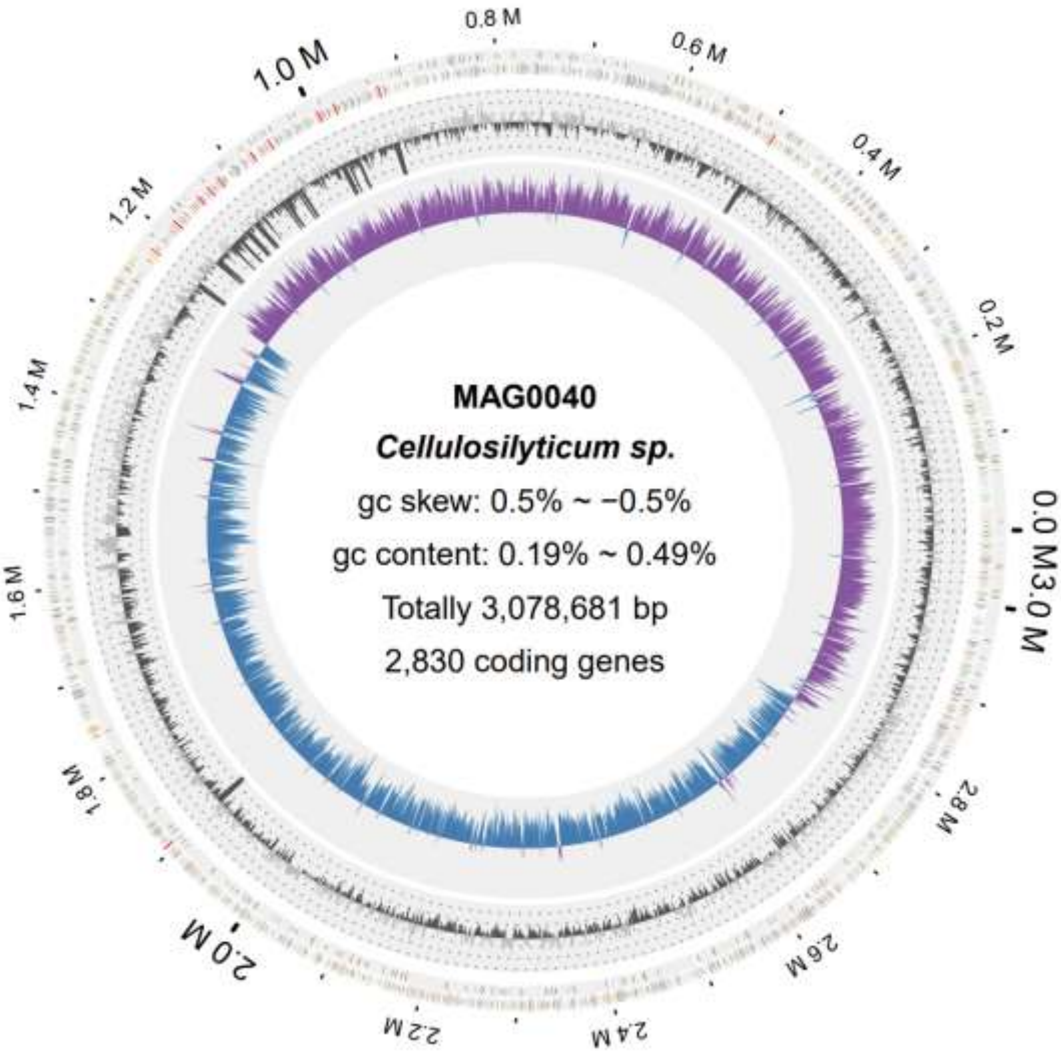
MAG0040
Cellulosilyticum sp.
gc skew: 0.5% ~ −0.5%
gc content: 0.19% ~ 0.49%
Totally 3,078,681 bp
2,830 coding genes
0.0 M
0.2 M
0.4 M
0.6 M
0.8 M
1.0 M
1.2 M
1.4 M
1.6 M
1.8 M
2.0 M
2.2 M
2.4 M
2.6 M
2.8 M
3.0 M
